# Supplementary material for: Telomere-to-telomere genome assemblies and population resequencing of diploid and allotetraploid peanut varieties
Source: Nat Genet. 2026 Apr 24;58(5):1151–63. doi: 10.1038/s41588-026-02577-z (PMC13175896; doi:10.1038/s41588-026-02577-z)
Supplement: Supplementary file 1 — Supplementary Figs. 1–27 and Notes. [file 41588_2026_2577_MOESM1_ESM.pdf]

nature genetics

Supplementary information

https://doi.org/10.1038/s41588-026-02577-z

Telomere-to-telomere genome assemblies and population resequencing of diploid and allotetraploid peanut varieties

In the format provided by the authors and unedited

Supplementary Figures

Supplementary Fig. 1 Hi-C chromatin interaction map of the six assembly peanut genomes.

Supplementary Fig. 2 Merqury copy number spectrum plots for the six assembled peanut genomes.

Supplementary Fig. 3 Genome synteny comparison between this study and published accessions.

Supplementary Fig. 4 Different insertion events of *Gypsy*-type LTRs in diploid and tetraploid genomes.

Supplementary Fig. 5 Different insertion times of Gypsy and *Copia*-type LTRs in different chromosomes.

Supplementary Fig. 6 Structural Variations Among Peanut Genomes.

Supplementary Fig. 7 Transposable elements (TEs) and their association with structural variations (SVs) based on the peanut genome.

Supplementary Fig. 8 PCR validations for translocations, inversions, and duplications identified among different genomes.

Supplementary Fig. 9 Collinearity analysis among peanut assemblies.

Supplementary Fig. 10 Syntenic analyses between Ad vs Bd, At vs Ad and Bt vs Bd.

Supplementary Fig. 11 Structural variation (SV) among different botanical varieties.

Supplementary Fig. 12 The expression pattern of structural variation (SV) genes present on Chromosome 14 in the S83 accession across different tissues.

Supplementary Fig. 13 Phylogenetic tree of 521 peanut accessions.

Supplementary Fig. 14 Population structure and demographic history of peanut populations.

Supplementary Fig. 15 LTR insertion on selected sweep regions.

Supplementary Fig. 16 Selected sweep and introgression among different populations.

Supplementary Fig. 17 SNP Variation Types in Selected and Introgressed Regions.

Supplementary Fig. 18 Percentage of share and specific variation between diploids and tetraploids.

Supplementary Fig. 19 Enrichment analyses of significantly selected and introgression among different populations.

Supplementary Fig. 20 Tissue-specific expression of *AhWRI1* gene.

Supplementary Fig. 21 The expression levels of *AhFUS3*, *AhABI3*, *AhLECs*, and their target gene *AhWRI1* in high and low oil content peanut accessions.

Supplementary Fig. 22 The oil content identification of overexpressing *AhWRI1* in *Glycine max* plant.

Supplementary Fig. 23 Tissue-specific expression of *AhGSA1* gene.

Supplementary Fig. 24 WGCNA analysis and network construction.

Supplementary Fig. 25 Evolutionary tree of transcription factors and enzymes in the peanut genome.

Supplementary Fig. 26 The flowchart of genome assembly.

Supplementary Fig. 27 GWAS for 33 traits.

Supplementary Notes

a

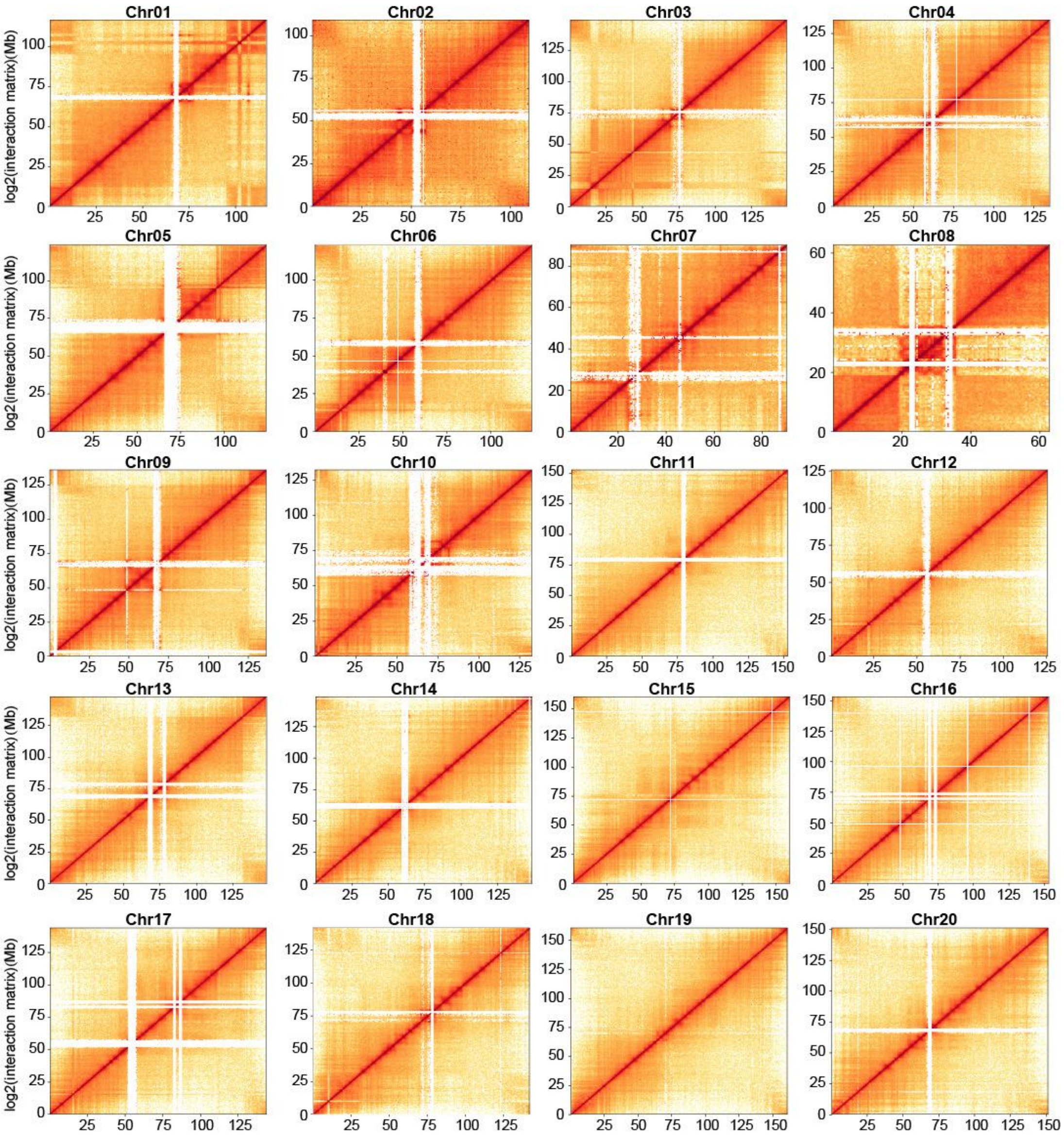
Chr01
Chr02
Chr03
Chr04
Chr05
Chr06
Chr07
Chr08
Chr09
Chr10
Chr11
Chr12
Chr13
Chr14
Chr15
Chr16
Chr17
Chr18
Chr19
Chr20
log2(interaction matrix)(Mb)

b

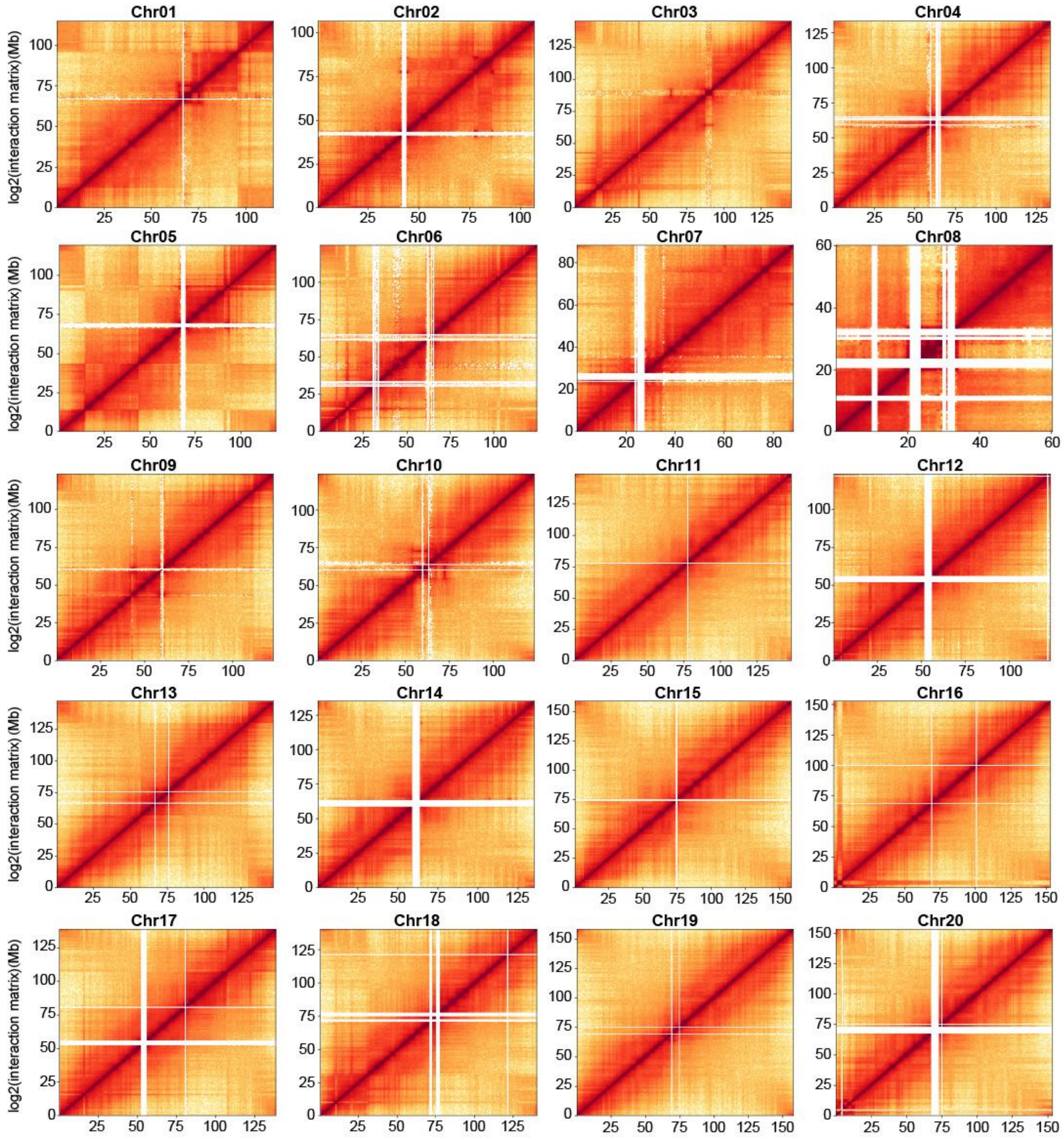

Chr01
Chr02
Chr03
Chr04
Chr05
Chr06
Chr07
Chr08
Chr09
Chr10
Chr11
Chr12
Chr13
Chr14
Chr15
Chr16
Chr17
Chr18
Chr19
Chr20
log2(interaction matrix)(Mb)

c

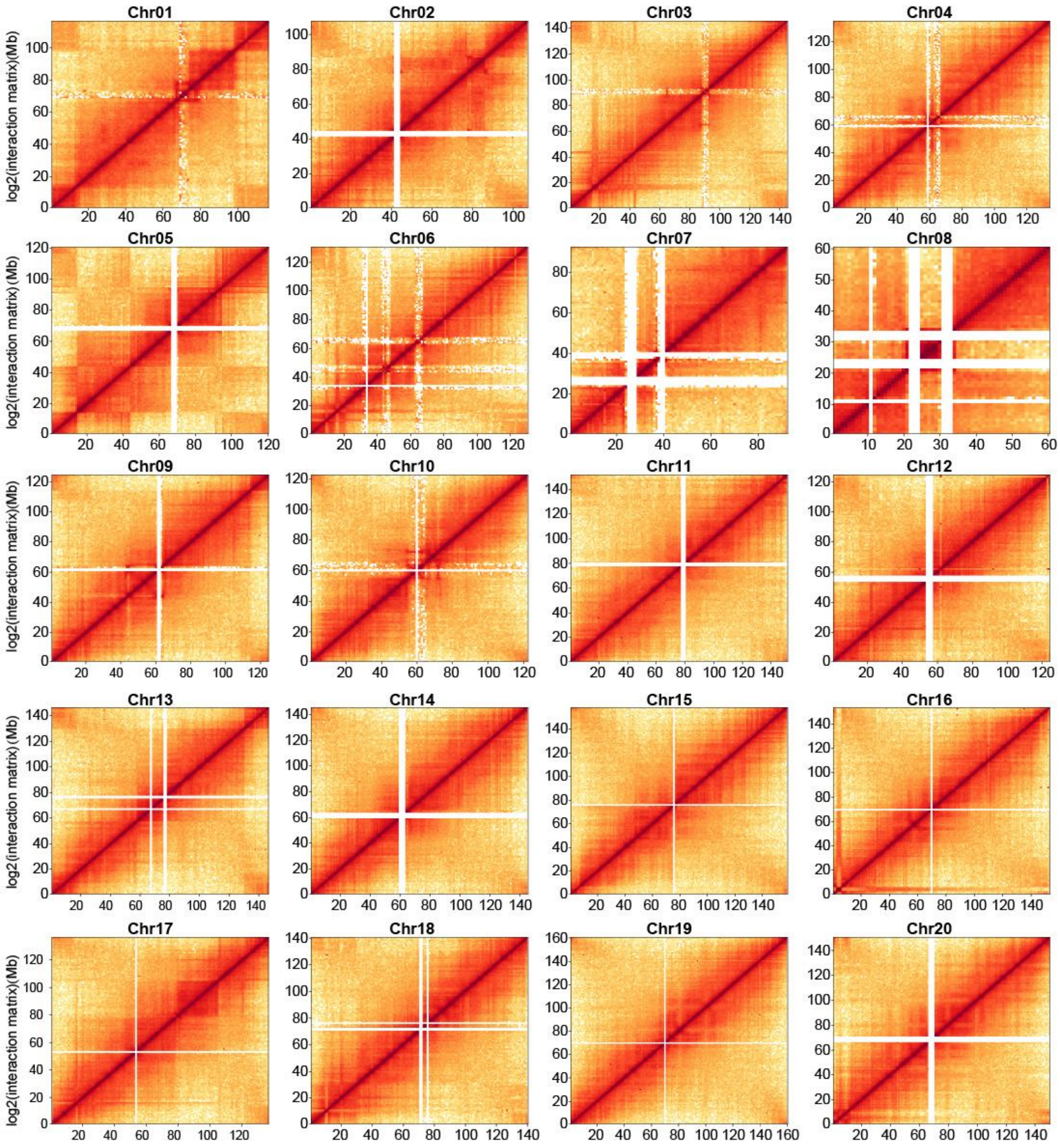

Chr01
Chr02
Chr03
Chr04
Chr05
Chr06
Chr07
Chr08
Chr09
Chr10
Chr11
Chr12
Chr13
Chr14
Chr15
Chr16
Chr17
Chr18
Chr19
Chr20
log2(interaction matrix)(Mb)

d

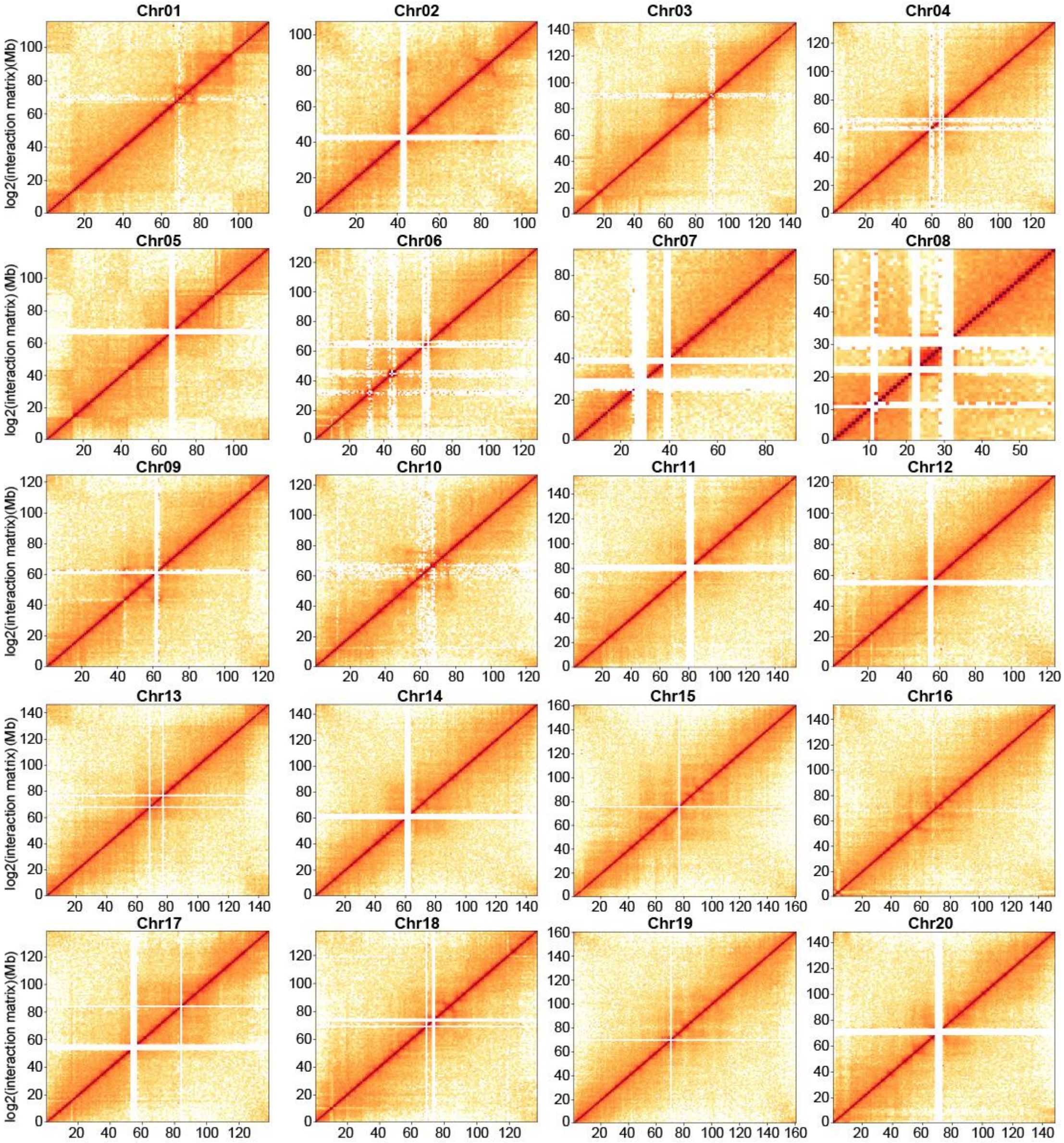
Chr01
Chr02
Chr03
Chr04
Chr05
Chr06
Chr07
Chr08
Chr09
Chr10
Chr11
Chr12
Chr13
Chr14
Chr15
Chr16
Chr17
Chr18
Chr19
Chr20
log2(interaction matrix)(Mb)

e

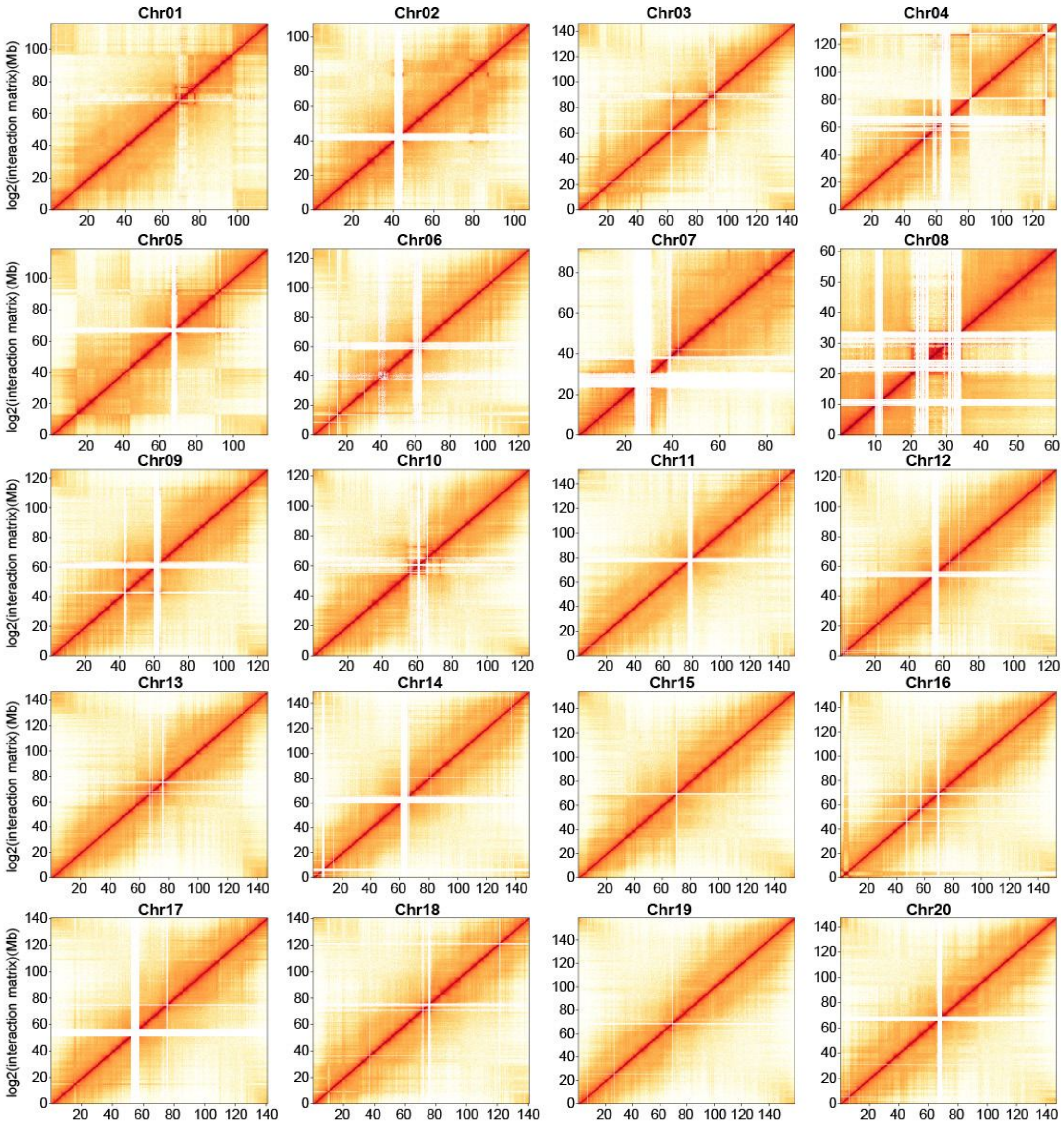

Supplementary Fig. 1 Hi-C chromatin interaction map of the six assembly peanut genomes. Synthetic intra-chromosomal matrix of chr01 to chr20, respectively. **a,** V14167(Chr01-Chr10) and K30076(Chr11-Chr20) accessions. **b-e,** S245, HN873, HN51 and S83 accessions. The colored bar on the right side of the Figure indicates the strength of interaction.

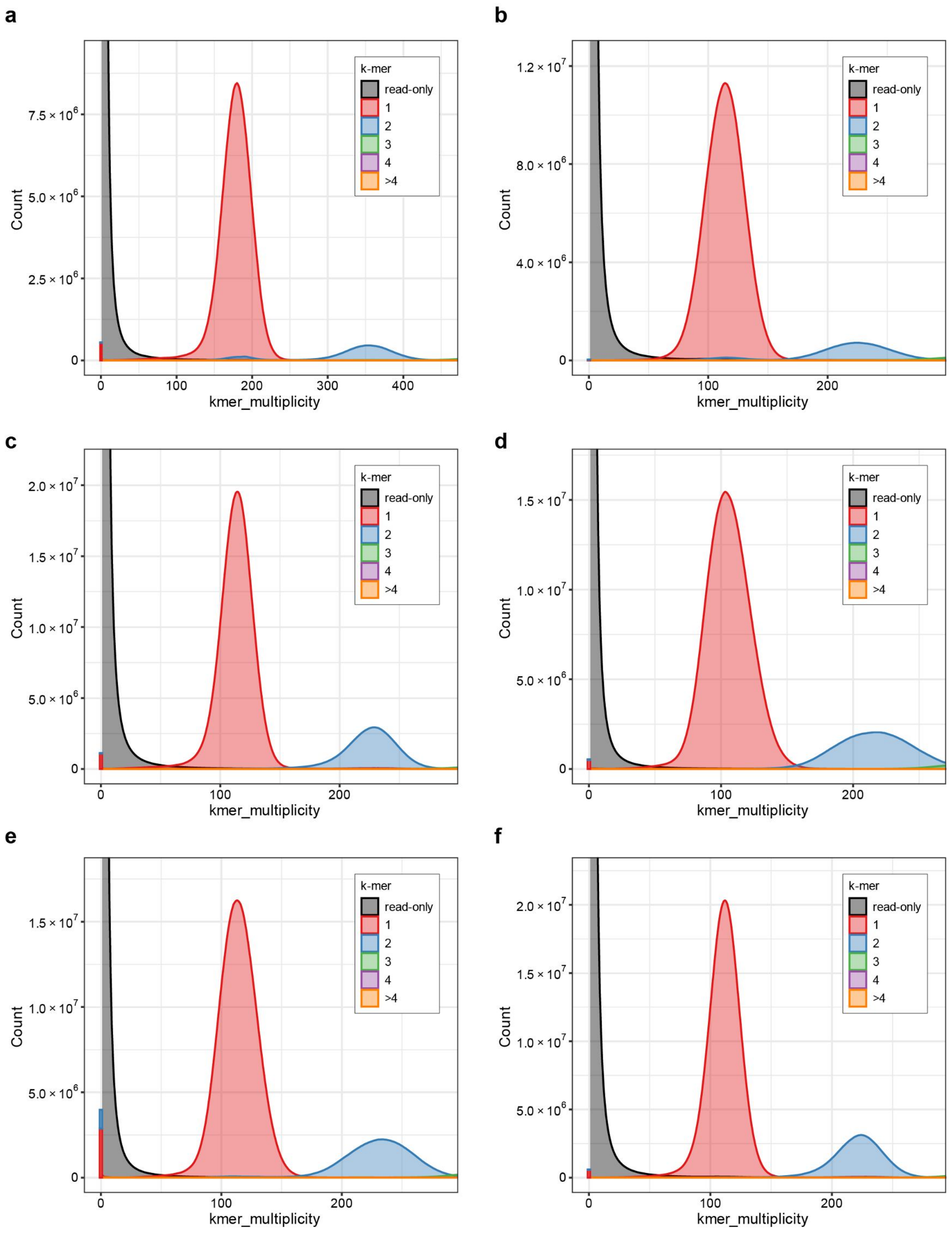

Supplementary Fig. 2 Merqury copy number spectrum plots for the six assembled peanut genomes. The copy number spectrum (spectra-cn) of the same K-mers is plotted as stacked histograms, colored by the copy numbers found in the combined assembly. The samples are: **a,** V14167; **b,** K30076; **c,** S245; **d,** HN873; **e,** HN51; **f,** S83.

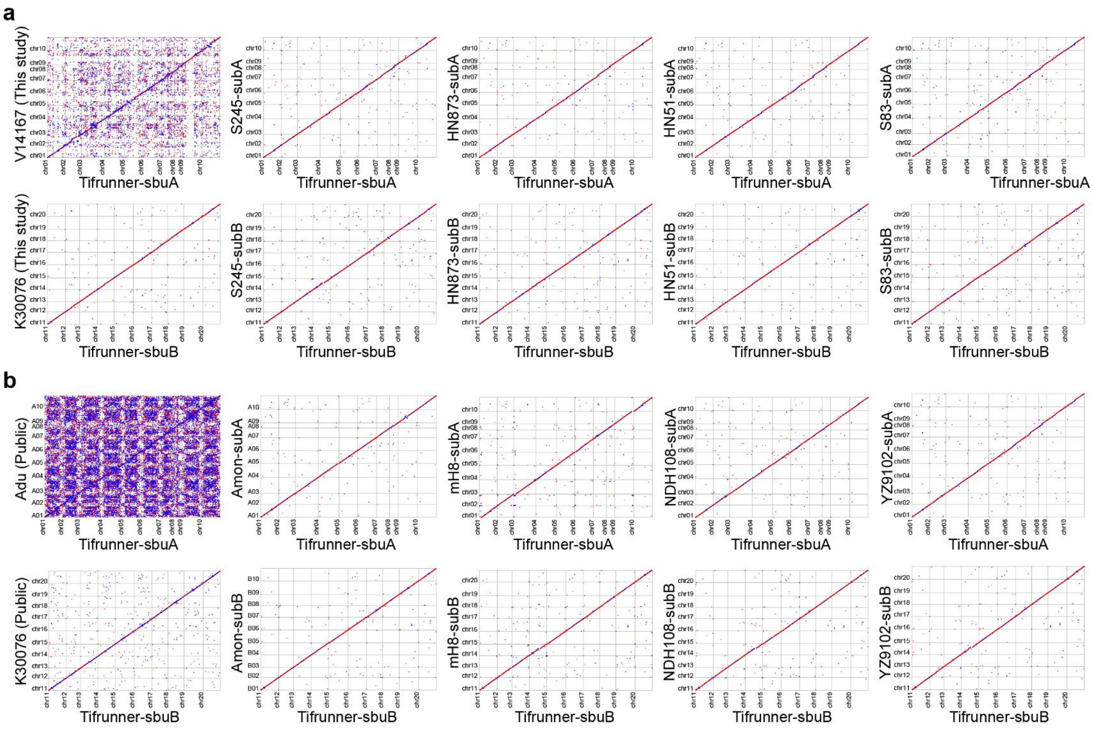

Supplementary Fig. 3 Genome synteny comparison between this study and published accessions. Genomic synteny analysis between the A genome/subgenome vs. Tifrunner_subA and B genome/subgenome vs. Tifrunner_subB, with Tifrunner as the reference (Bertioli et al. (2019)[1]). **a,** All six T2T genome assemblies in this study. **b,** Six genomes from previously published articles, including the K30076 genome from Bertioli et al. (2016)[2]. The Adu, Amon, mH8, and NHD108 genomes are from Zhao et al. (2025)[3]; YZ9102 is from Wang et al. (2025)[4]. Except for YZ9102, which is a T2T genome, the contig sequences in the other five genomes were deleted prior to alignment, leaving only chromosome sequences.

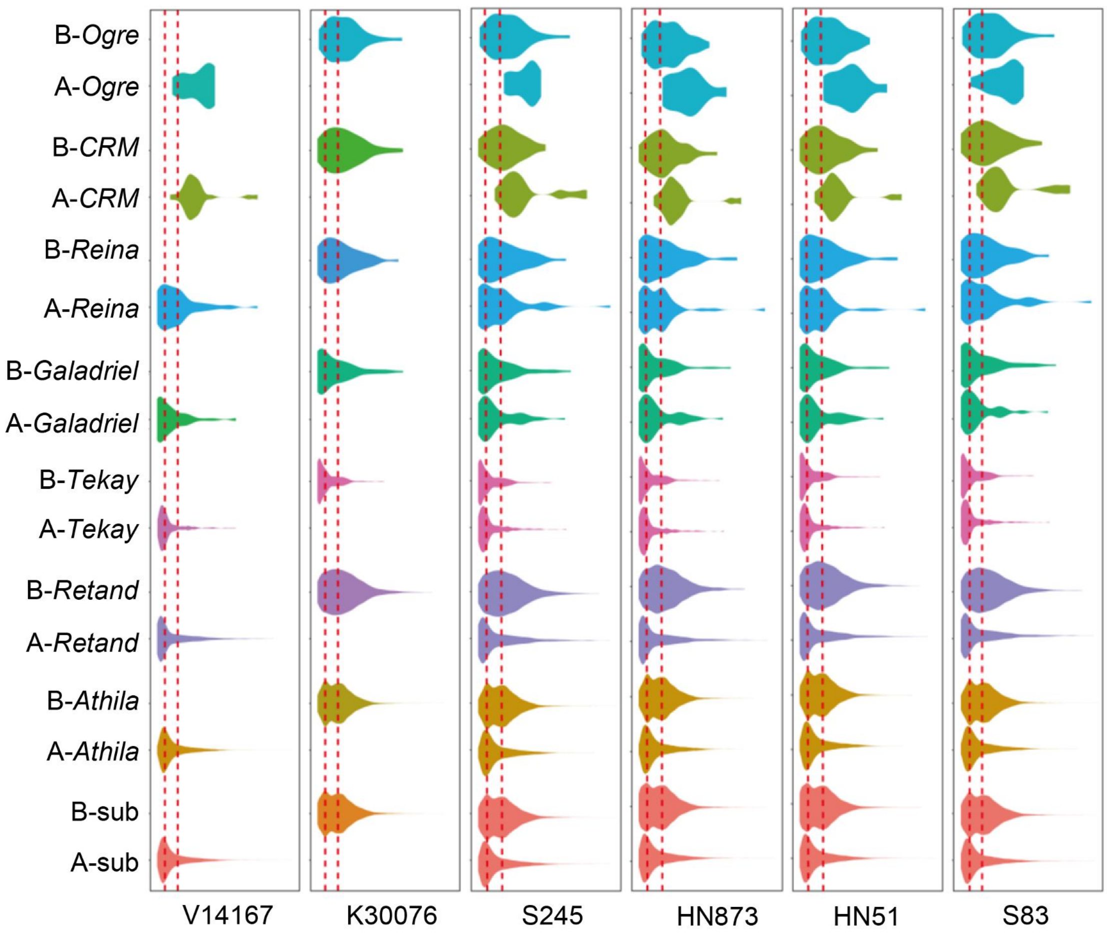

Supplementary Fig. 4 Different insertion events of *Gypsy*-type LTRs in diploid and tetraploid genomes. Gypsy-type LTRs divided into seven subfamilies, including: *Athila*, *Retand*, *Tekay*, *Galadriel*, *Reina*, *CRM* and *Ogre*. The peak represents an expansion event, with the dashed line on the right indicating the first expansion occurring around 0.69 million years ago (Mya), and the dashed line on the left indicating the second expansion occurring around 0.27 Mya.

a

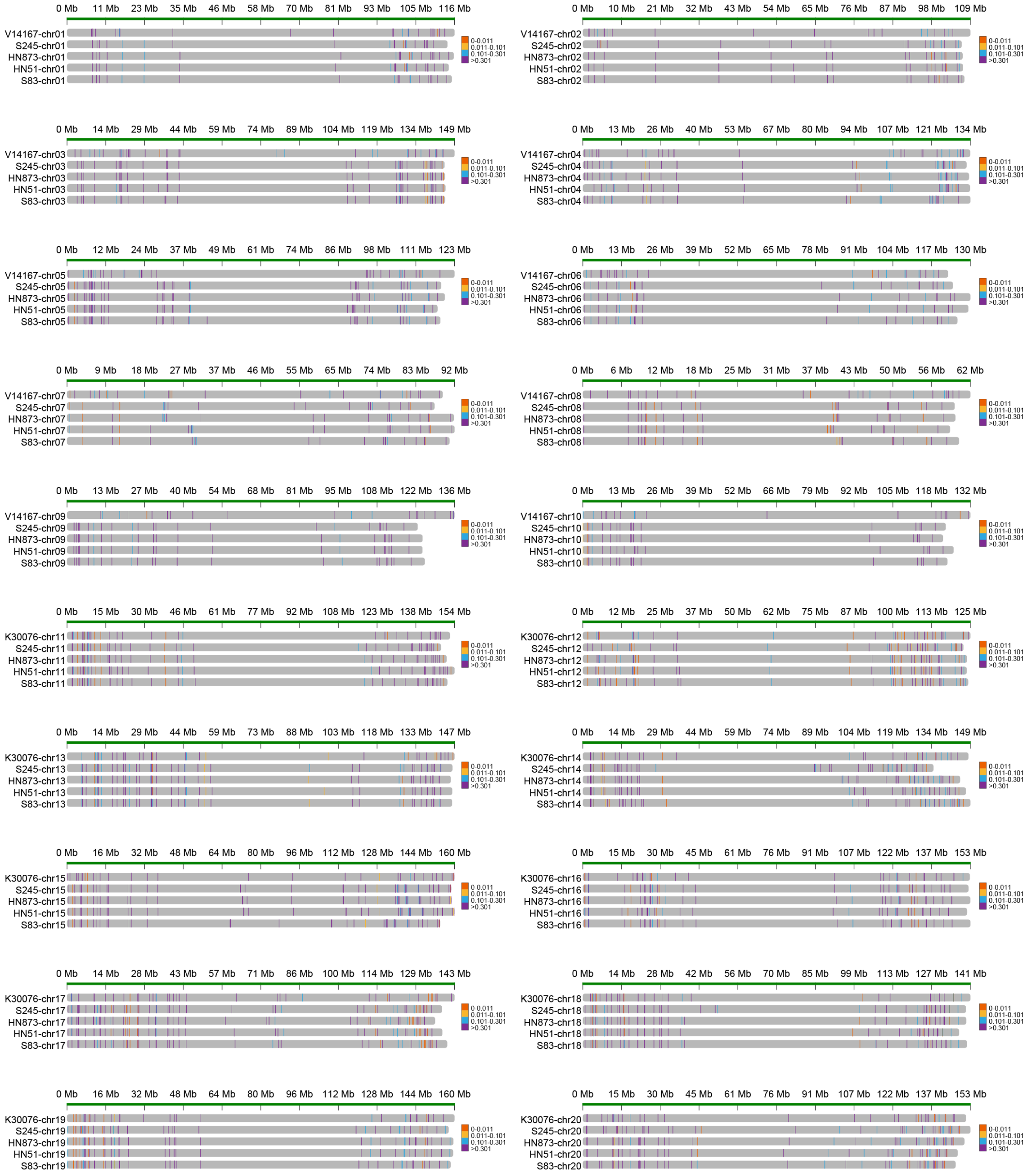

b

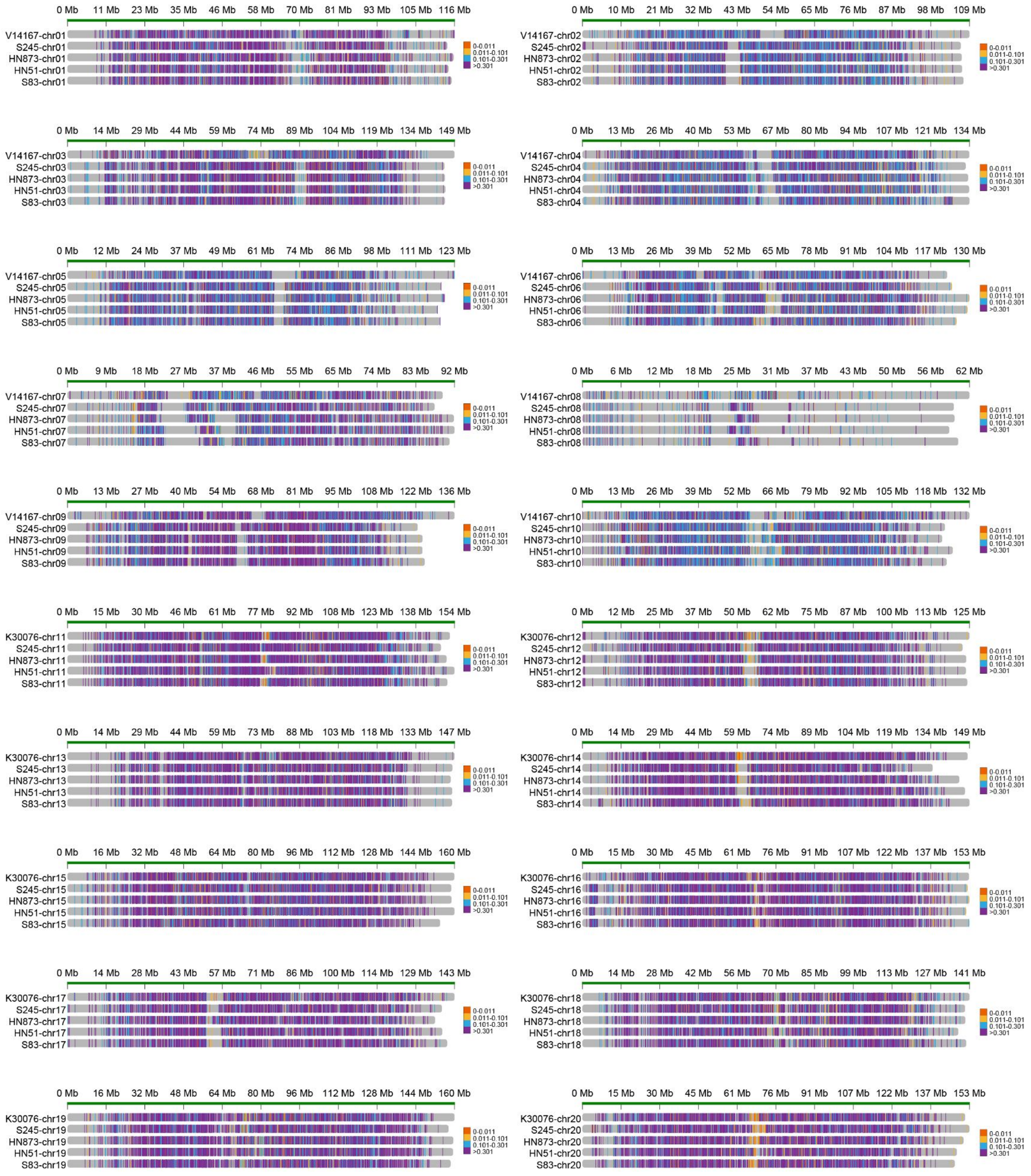
0 Mb 11 Mb 23 Mb 35 Mb 46 Mb 58 Mb 70 Mb 81 Mb 93 Mb 105 Mb 116 Mb
V14167-chr01
S245-chr01
HN873-chr01
HN51-chr01
S83-chr01
0 Mb 10 Mb 21 Mb 32 Mb 43 Mb 54 Mb 65 Mb 76 Mb 87 Mb 98 Mb 109 Mb
V14167-chr02
S245-chr02
HN873-chr02
HN51-chr02
S83-chr02
0 Mb 14 Mb 29 Mb 44 Mb 59 Mb 74 Mb 89 Mb 104 Mb 119 Mb 134 Mb 149 Mb
V14167-chr03
S245-chr03
HN873-chr03
HN51-chr03
S83-chr03
0 Mb 13 Mb 26 Mb 40 Mb 53 Mb 67 Mb 80 Mb 94 Mb 107 Mb 121 Mb 134 Mb
V14167-chr04
S245-chr04
HN873-chr04
HN51-chr04
S83-chr04
0 Mb 12 Mb 24 Mb 37 Mb 49 Mb 61 Mb 74 Mb 86 Mb 98 Mb 111 Mb 123 Mb
V14167-chr05
S245-chr05
HN873-chr05
HN51-chr05
S83-chr05
0 Mb 13 Mb 26 Mb 39 Mb 52 Mb 65 Mb 78 Mb 91 Mb 104 Mb 117 Mb 130 Mb
V14167-chr06
S245-chr06
HN873-chr06
HN51-chr06
S83-chr06
0 Mb 9 Mb 18 Mb 27 Mb 37 Mb 46 Mb 55 Mb 65 Mb 74 Mb 83 Mb 92 Mb
V14167-chr07
S245-chr07
HN873-chr07
HN51-chr07
S83-chr07
0 Mb 6 Mb 12 Mb 18 Mb 25 Mb 31 Mb 37 Mb 43 Mb 50 Mb 56 Mb 62 Mb
V14167-chr08
S245-chr08
HN873-chr08
HN51-chr08
S83-chr08
0 Mb 13 Mb 27 Mb 40 Mb 54 Mb 68 Mb 81 Mb 95 Mb 108 Mb 122 Mb 136 Mb
V14167-chr09
S245-chr09
HN873-chr09
HN51-chr09
S83-chr09
0 Mb 13 Mb 26 Mb 39 Mb 52 Mb 66 Mb 79 Mb 92 Mb 105 Mb 118 Mb 132 Mb
V14167-chr10
S245-chr10
HN873-chr10
HN51-chr10
S83-chr10
0 Mb 15 Mb 30 Mb 46 Mb 61 Mb 77 Mb 92 Mb 108 Mb 123 Mb 138 Mb 154 Mb
K30076-chr11
S245-chr11
HN873-chr11
HN51-chr11
S83-chr11
0 Mb 12 Mb 25 Mb 37 Mb 50 Mb 62 Mb 75 Mb 87 Mb 100 Mb 113 Mb 125 Mb
K30076-chr12
S245-chr12
HN873-chr12
HN51-chr12
S83-chr12
0 Mb 14 Mb 29 Mb 44 Mb 59 Mb 73 Mb 88 Mb 103 Mb 118 Mb 133 Mb 147 Mb
K30076-chr13
S245-chr13
HN873-chr13
HN51-chr13
S83-chr13
0 Mb 14 Mb 29 Mb 44 Mb 59 Mb 74 Mb 89 Mb 104 Mb 119 Mb 134 Mb 149 Mb
K30076-chr14
S245-chr14
HN873-chr14
HN51-chr14
S83-chr14
0 Mb 16 Mb 32 Mb 48 Mb 64 Mb 80 Mb 96 Mb 112 Mb 128 Mb 144 Mb 160 Mb
K30076-chr15
S245-chr15
HN873-chr15
HN51-chr15
S83-chr15
0 Mb 15 Mb 30 Mb 45 Mb 61 Mb 76 Mb 91 Mb 107 Mb 122 Mb 137 Mb 153 Mb
K30076-chr16
S245-chr16
HN873-chr16
HN51-chr16
S83-chr16
0 Mb 14 Mb 28 Mb 43 Mb 57 Mb 71 Mb 86 Mb 100 Mb 114 Mb 129 Mb 143 Mb
K30076-chr17
S245-chr17
HN873-chr17
HN51-chr17
S83-chr17
0 Mb 14 Mb 28 Mb 42 Mb 56 Mb 70 Mb 85 Mb 99 Mb 113 Mb 127 Mb 141 Mb
K30076-chr18
S245-chr18
HN873-chr18
HN51-chr18
S83-chr18
0 Mb 16 Mb 32 Mb 48 Mb 64 Mb 80 Mb 96 Mb 112 Mb 128 Mb 144 Mb 160 Mb
K30076-chr19
S245-chr19
HN873-chr19
HN51-chr19
S83-chr19
0 Mb 15 Mb 30 Mb 45 Mb 61 Mb 76 Mb 91 Mb 107 Mb 122 Mb 137 Mb 153 Mb
K30076-chr20
S245-chr20
HN873-chr20
HN51-chr20
S83-chr20
0-0.011
0.011-0.101
0.101-0.301
>0.301

c

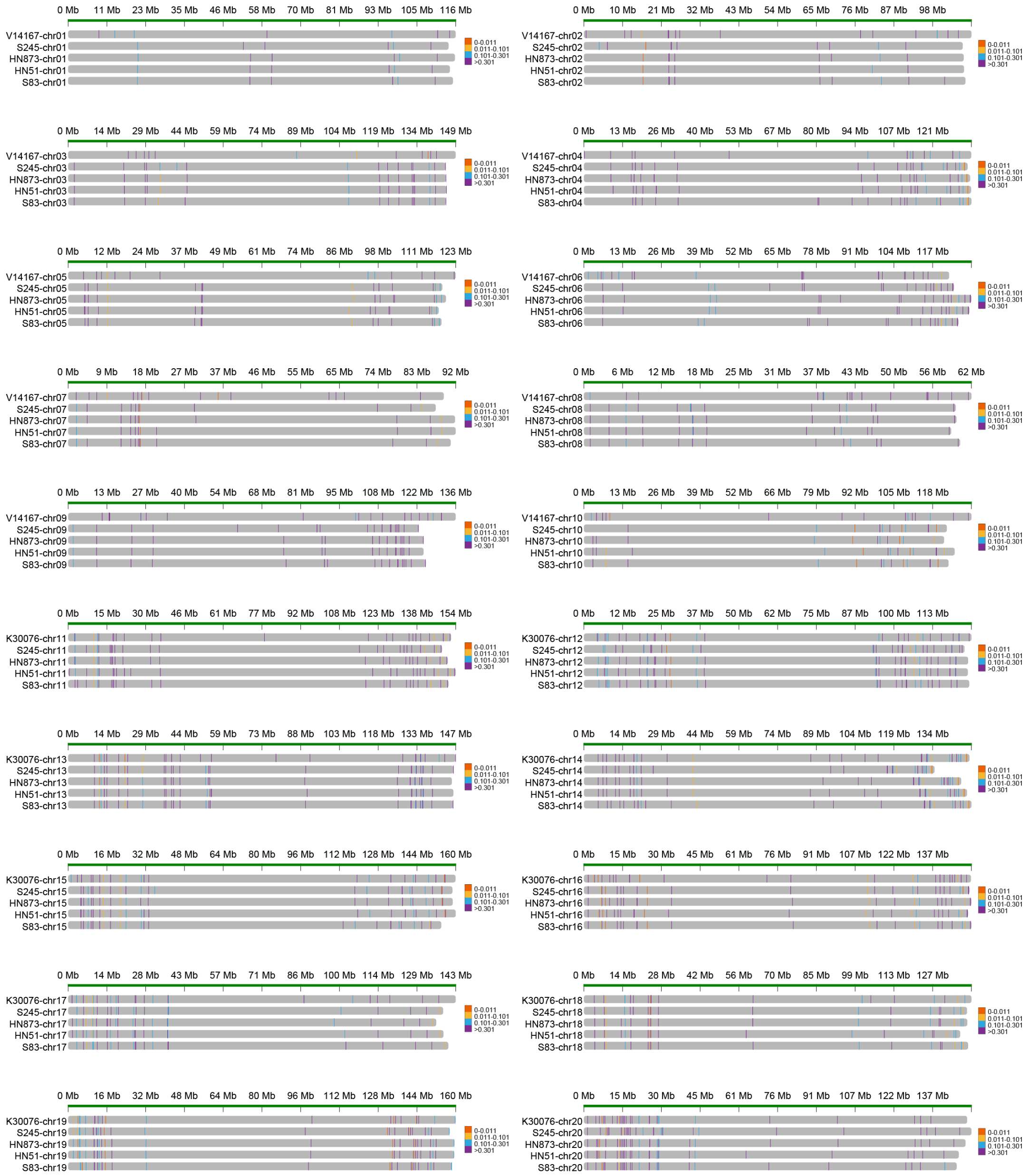

0 Mb 11 Mb 23 Mb 35 Mb 46 Mb 58 Mb 70 Mb 81 Mb 93 Mb 105 Mb 116 Mb
V14167-chr01
S245-chr01
HN873-chr01
HN51-chr01
S83-chr01
0 Mb 10 Mb 21 Mb 32 Mb 43 Mb 54 Mb 65 Mb 76 Mb 87 Mb 98 Mb
V14167-chr02
S245-chr02
HN873-chr02
HN51-chr02
S83-chr02
0 Mb 14 Mb 29 Mb 44 Mb 59 Mb 74 Mb 89 Mb 104 Mb 119 Mb 134 Mb 149 Mb
V14167-chr03
S245-chr03
HN873-chr03
HN51-chr03
S83-chr03
0 Mb 13 Mb 26 Mb 40 Mb 53 Mb 67 Mb 80 Mb 94 Mb 107 Mb 121 Mb
V14167-chr04
S245-chr04
HN873-chr04
HN51-chr04
S83-chr04
0 Mb 12 Mb 24 Mb 37 Mb 49 Mb 61 Mb 74 Mb 86 Mb 98 Mb 111 Mb 123 Mb
V14167-chr05
S245-chr05
HN873-chr05
HN51-chr05
S83-chr05
0 Mb 13 Mb 26 Mb 39 Mb 52 Mb 65 Mb 78 Mb 91 Mb 104 Mb 117 Mb
V14167-chr06
S245-chr06
HN873-chr06
HN51-chr06
S83-chr06
0 Mb 9 Mb 18 Mb 27 Mb 37 Mb 46 Mb 55 Mb 65 Mb 74 Mb 83 Mb 92 Mb
V14167-chr07
S245-chr07
HN873-chr07
HN51-chr07
S83-chr07
0 Mb 6 Mb 12 Mb 18 Mb 25 Mb 31 Mb 37 Mb 43 Mb 50 Mb 56 Mb 62 Mb
V14167-chr08
S245-chr08
HN873-chr08
HN51-chr08
S83-chr08
0 Mb 13 Mb 27 Mb 40 Mb 54 Mb 68 Mb 81 Mb 95 Mb 108 Mb 122 Mb 136 Mb
V14167-chr09
S245-chr09
HN873-chr09
HN51-chr09
S83-chr09
0 Mb 13 Mb 26 Mb 39 Mb 52 Mb 66 Mb 79 Mb 92 Mb 105 Mb 118 Mb
V14167-chr10
S245-chr10
HN873-chr10
HN51-chr10
S83-chr10
0 Mb 15 Mb 30 Mb 46 Mb 61 Mb 77 Mb 92 Mb 108 Mb 123 Mb 138 Mb 154 Mb
K30076-chr11
S245-chr11
HN873-chr11
HN51-chr11
S83-chr11
0 Mb 12 Mb 25 Mb 37 Mb 50 Mb 62 Mb 75 Mb 87 Mb 100 Mb 113 Mb
K30076-chr12
S245-chr12
HN873-chr12
HN51-chr12
S83-chr12
0 Mb 14 Mb 29 Mb 44 Mb 59 Mb 73 Mb 88 Mb 103 Mb 118 Mb 133 Mb 147 Mb
K30076-chr13
S245-chr13
HN873-chr13
HN51-chr13
S83-chr13
0 Mb 14 Mb 29 Mb 44 Mb 59 Mb 74 Mb 89 Mb 104 Mb 119 Mb 134 Mb
K30076-chr14
S245-chr14
HN873-chr14
HN51-chr14
S83-chr14
0 Mb 16 Mb 32 Mb 48 Mb 64 Mb 80 Mb 96 Mb 112 Mb 128 Mb 144 Mb 160 Mb
K30076-chr15
S245-chr15
HN873-chr15
HN51-chr15
S83-chr15
0 Mb 15 Mb 30 Mb 45 Mb 61 Mb 76 Mb 91 Mb 107 Mb 122 Mb 137 Mb
K30076-chr16
S245-chr16
HN873-chr16
HN51-chr16
S83-chr16
0 Mb 14 Mb 28 Mb 43 Mb 57 Mb 71 Mb 86 Mb 100 Mb 114 Mb 129 Mb 143 Mb
K30076-chr17
S245-chr17
HN873-chr17
HN51-chr17
S83-chr17
0 Mb 14 Mb 28 Mb 42 Mb 56 Mb 70 Mb 85 Mb 99 Mb 113 Mb 127 Mb
K30076-chr18
S245-chr18
HN873-chr18
HN51-chr18
S83-chr18
0 Mb 16 Mb 32 Mb 48 Mb 64 Mb 80 Mb 96 Mb 112 Mb 128 Mb 144 Mb 160 Mb
K30076-chr19
S245-chr19
HN873-chr19
HN51-chr19
S83-chr19
0 Mb 15 Mb 30 Mb 45 Mb 61 Mb 76 Mb 91 Mb 107 Mb 122 Mb 137 Mb
K30076-chr20
S245-chr20
HN873-chr20
HN51-chr20
S83-chr20
0-0.011
0.011-0.101
0.101-0.301
>0.301

d

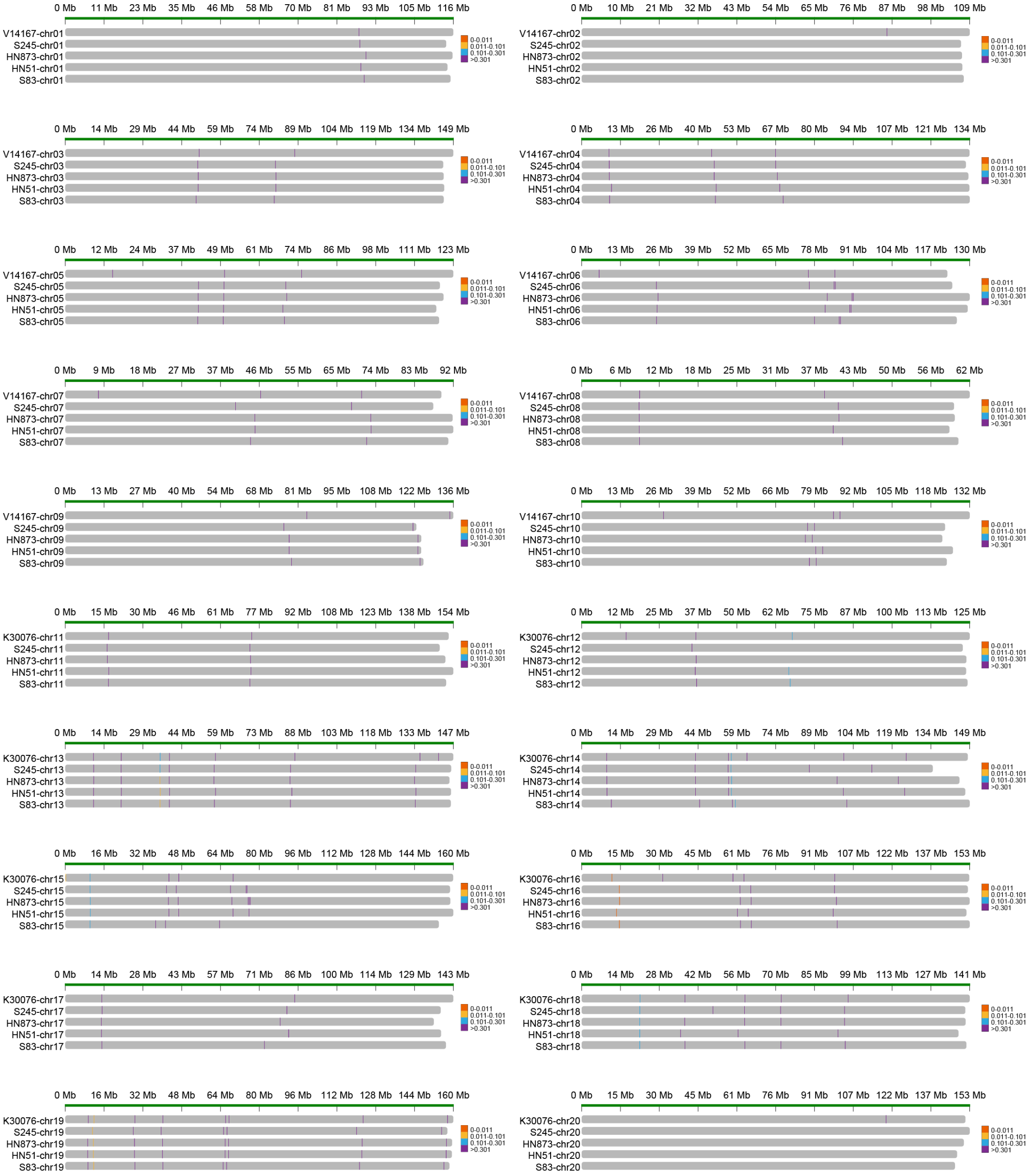
0 Mb 11 Mb 23 Mb 35 Mb 46 Mb 58 Mb 70 Mb 81 Mb 93 Mb 105 Mb 116 Mb
V14167-chr01
S245-chr01
HN873-chr01
HN51-chr01
S83-chr01
0 Mb 10 Mb 21 Mb 32 Mb 43 Mb 54 Mb 65 Mb 76 Mb 87 Mb 98 Mb 109 Mb
V14167-chr02
S245-chr02
HN873-chr02
HN51-chr02
S83-chr02
0 Mb 14 Mb 29 Mb 44 Mb 59 Mb 74 Mb 89 Mb 104 Mb 119 Mb 134 Mb 149 Mb
V14167-chr03
S245-chr03
HN873-chr03
HN51-chr03
S83-chr03
0 Mb 13 Mb 26 Mb 40 Mb 53 Mb 67 Mb 80 Mb 94 Mb 107 Mb 121 Mb 134 Mb
V14167-chr04
S245-chr04
HN873-chr04
HN51-chr04
S83-chr04
0 Mb 12 Mb 24 Mb 37 Mb 49 Mb 61 Mb 74 Mb 86 Mb 98 Mb 111 Mb 123 Mb
V14167-chr05
S245-chr05
HN873-chr05
HN51-chr05
S83-chr05
0 Mb 13 Mb 26 Mb 39 Mb 52 Mb 65 Mb 78 Mb 91 Mb 104 Mb 117 Mb 130 Mb
V14167-chr06
S245-chr06
HN873-chr06
HN51-chr06
S83-chr06
0 Mb 9 Mb 18 Mb 27 Mb 37 Mb 46 Mb 55 Mb 65 Mb 74 Mb 83 Mb 92 Mb
V14167-chr07
S245-chr07
HN873-chr07
HN51-chr07
S83-chr07
0 Mb 6 Mb 12 Mb 18 Mb 25 Mb 31 Mb 37 Mb 43 Mb 50 Mb 56 Mb 62 Mb
V14167-chr08
S245-chr08
HN873-chr08
HN51-chr08
S83-chr08
0 Mb 13 Mb 27 Mb 40 Mb 54 Mb 68 Mb 81 Mb 95 Mb 108 Mb 122 Mb 136 Mb
V14167-chr09
S245-chr09
HN873-chr09
HN51-chr09
S83-chr09
0 Mb 13 Mb 26 Mb 39 Mb 52 Mb 66 Mb 79 Mb 92 Mb 105 Mb 118 Mb 132 Mb
V14167-chr10
S245-chr10
HN873-chr10
HN51-chr10
S83-chr10
0 Mb 15 Mb 30 Mb 46 Mb 61 Mb 77 Mb 92 Mb 108 Mb 123 Mb 138 Mb 154 Mb
K30076-chr11
S245-chr11
HN873-chr11
HN51-chr11
S83-chr11
0 Mb 12 Mb 25 Mb 37 Mb 50 Mb 62 Mb 75 Mb 87 Mb 100 Mb 113 Mb 125 Mb
K30076-chr12
S245-chr12
HN873-chr12
HN51-chr12
S83-chr12
0 Mb 14 Mb 29 Mb 44 Mb 59 Mb 73 Mb 88 Mb 103 Mb 118 Mb 133 Mb 147 Mb
K30076-chr13
S245-chr13
HN873-chr13
HN51-chr13
S83-chr13
0 Mb 14 Mb 29 Mb 44 Mb 59 Mb 74 Mb 89 Mb 104 Mb 119 Mb 134 Mb 149 Mb
K30076-chr14
S245-chr14
HN873-chr14
HN51-chr14
S83-chr14
0 Mb 16 Mb 32 Mb 48 Mb 64 Mb 80 Mb 96 Mb 112 Mb 128 Mb 144 Mb 160 Mb
K30076-chr15
S245-chr15
HN873-chr15
HN51-chr15
S83-chr15
0 Mb 15 Mb 30 Mb 45 Mb 61 Mb 76 Mb 91 Mb 107 Mb 122 Mb 137 Mb 153 Mb
K30076-chr16
S245-chr16
HN873-chr16
HN51-chr16
S83-chr16
0 Mb 14 Mb 28 Mb 43 Mb 57 Mb 71 Mb 86 Mb 100 Mb 114 Mb 129 Mb 143 Mb
K30076-chr17
S245-chr17
HN873-chr17
HN51-chr17
S83-chr17
0 Mb 14 Mb 28 Mb 42 Mb 56 Mb 70 Mb 85 Mb 99 Mb 113 Mb 127 Mb 141 Mb
K30076-chr18
S245-chr18
HN873-chr18
HN51-chr18
S83-chr18
0 Mb 16 Mb 32 Mb 48 Mb 64 Mb 80 Mb 96 Mb 112 Mb 128 Mb 144 Mb 160 Mb
K30076-chr19
S245-chr19
HN873-chr19
HN51-chr19
S83-chr19
0 Mb 15 Mb 30 Mb 45 Mb 61 Mb 76 Mb 91 Mb 107 Mb 122 Mb 137 Mb 153 Mb
K30076-chr20
S245-chr20
HN873-chr20
HN51-chr20
S83-chr20

e

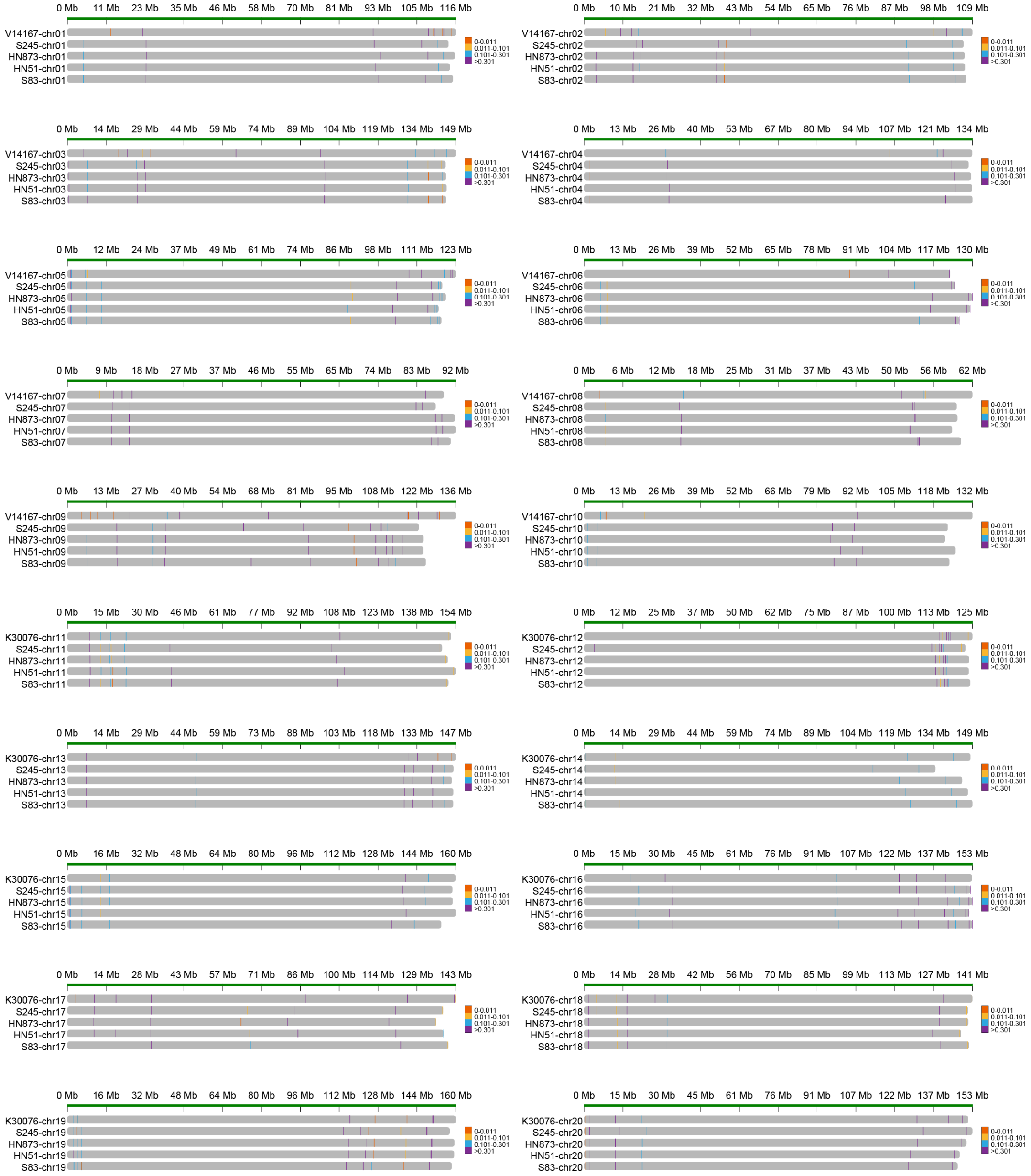

V14167-chr01
S245-chr01
HN873-chr01
HN51-chr01
S83-chr01
V14167-chr02
S245-chr02
HN873-chr02
HN51-chr02
S83-chr02
V14167-chr03
S245-chr03
HN873-chr03
HN51-chr03
S83-chr03
V14167-chr04
S245-chr04
HN873-chr04
HN51-chr04
S83-chr04
V14167-chr05
S245-chr05
HN873-chr05
HN51-chr05
S83-chr05
V14167-chr06
S245-chr06
HN873-chr06
HN51-chr06
S83-chr06
V14167-chr07
S245-chr07
HN873-chr07
HN51-chr07
S83-chr07
V14167-chr08
S245-chr08
HN873-chr08
HN51-chr08
S83-chr08
V14167-chr09
S245-chr09
HN873-chr09
HN51-chr09
S83-chr09
V14167-chr10
S245-chr10
HN873-chr10
HN51-chr10
S83-chr10
K30076-chr11
S245-chr11
HN873-chr11
HN51-chr11
S83-chr11
K30076-chr12
S245-chr12
HN873-chr12
HN51-chr12
S83-chr12
K30076-chr13
S245-chr13
HN873-chr13
HN51-chr13
S83-chr13
K30076-chr14
S245-chr14
HN873-chr14
HN51-chr14
S83-chr14
K30076-chr15
S245-chr15
HN873-chr15
HN51-chr15
S83-chr15
K30076-chr16
S245-chr16
HN873-chr16
HN51-chr16
S83-chr16
K30076-chr17
S245-chr17
HN873-chr17
HN51-chr17
S83-chr17
K30076-chr18
S245-chr18
HN873-chr18
HN51-chr18
S83-chr18
K30076-chr19
S245-chr19
HN873-chr19
HN51-chr19
S83-chr19
K30076-chr20
S245-chr20
HN873-chr20
HN51-chr20
S83-chr20
0-0.011
0.011-0.101
0.101-0.301
>0.301

f

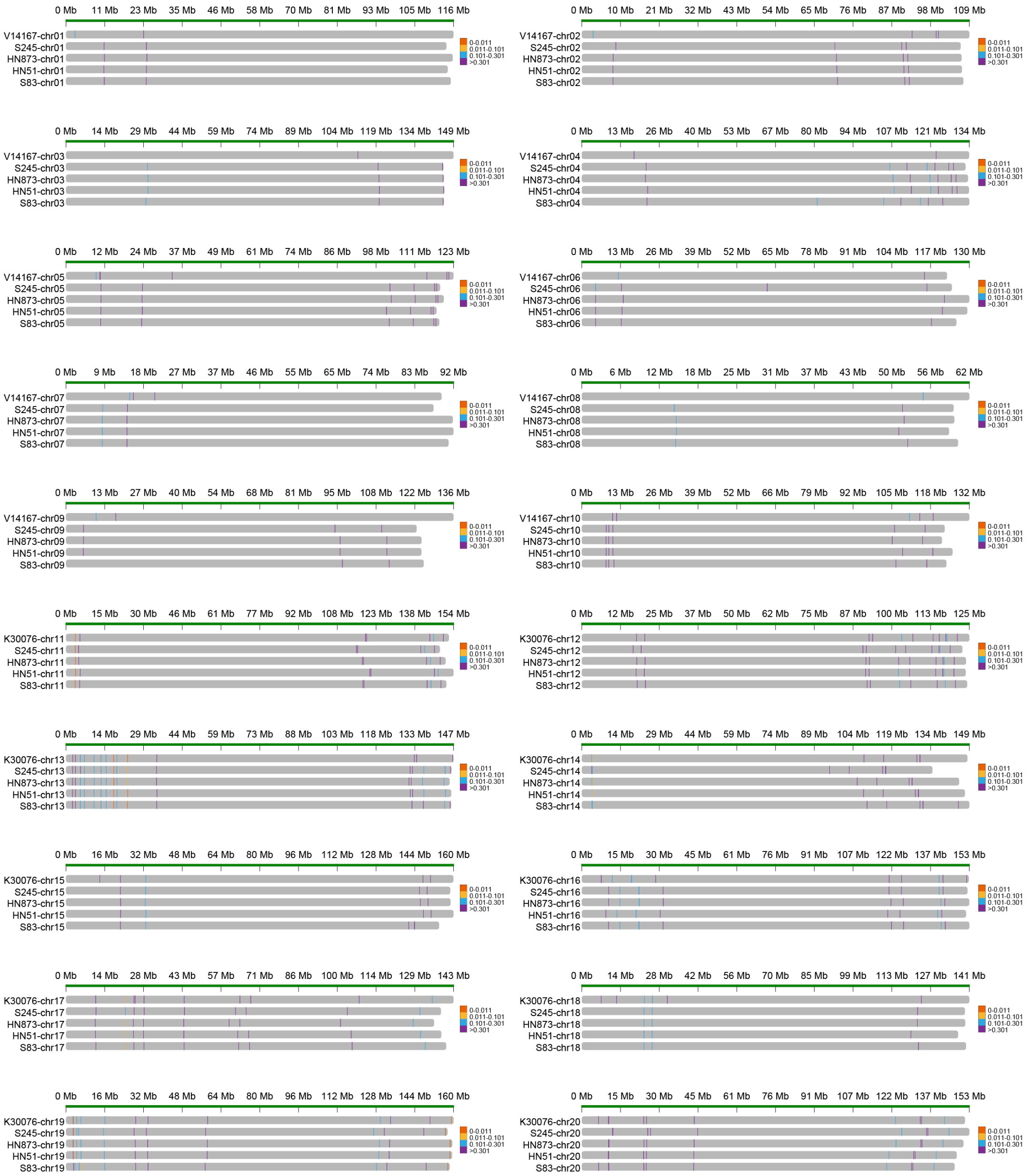
0 Mb 11 Mb 23 Mb 35 Mb 46 Mb 58 Mb 70 Mb 81 Mb 93 Mb 105 Mb 116 Mb
V14167-chr01
S245-chr01
HN873-chr01
HN51-chr01
S83-chr01
0-0.011
0.011-0.101
0.101-0.301
>0.301
0 Mb 10 Mb 21 Mb 32 Mb 43 Mb 54 Mb 65 Mb 76 Mb 87 Mb 98 Mb 109 Mb
V14167-chr02
S245-chr02
HN873-chr02
HN51-chr02
S83-chr02
0 Mb 14 Mb 29 Mb 44 Mb 59 Mb 74 Mb 89 Mb 104 Mb 119 Mb 134 Mb 149 Mb
V14167-chr03
S245-chr03
HN873-chr03
HN51-chr03
S83-chr03
0 Mb 13 Mb 26 Mb 40 Mb 53 Mb 67 Mb 80 Mb 94 Mb 107 Mb 121 Mb 134 Mb
V14167-chr04
S245-chr04
HN873-chr04
HN51-chr04
S83-chr04
0 Mb 12 Mb 24 Mb 37 Mb 49 Mb 61 Mb 74 Mb 86 Mb 98 Mb 111 Mb 123 Mb
V14167-chr05
S245-chr05
HN873-chr05
HN51-chr05
S83-chr05
0 Mb 13 Mb 26 Mb 39 Mb 52 Mb 65 Mb 78 Mb 91 Mb 104 Mb 117 Mb 130 Mb
V14167-chr06
S245-chr06
HN873-chr06
HN51-chr06
S83-chr06
0 Mb 9 Mb 18 Mb 27 Mb 37 Mb 46 Mb 55 Mb 65 Mb 74 Mb 83 Mb 92 Mb
V14167-chr07
S245-chr07
HN873-chr07
HN51-chr07
S83-chr07
0 Mb 6 Mb 12 Mb 18 Mb 25 Mb 31 Mb 37 Mb 43 Mb 50 Mb 56 Mb 62 Mb
V14167-chr08
S245-chr08
HN873-chr08
HN51-chr08
S83-chr08
0 Mb 13 Mb 27 Mb 40 Mb 54 Mb 68 Mb 81 Mb 95 Mb 108 Mb 122 Mb 136 Mb
V14167-chr09
S245-chr09
HN873-chr09
HN51-chr09
S83-chr09
0 Mb 13 Mb 26 Mb 39 Mb 52 Mb 66 Mb 79 Mb 92 Mb 105 Mb 118 Mb 132 Mb
V14167-chr10
S245-chr10
HN873-chr10
HN51-chr10
S83-chr10
0 Mb 15 Mb 30 Mb 46 Mb 61 Mb 77 Mb 92 Mb 108 Mb 123 Mb 138 Mb 154 Mb
K30076-chr11
S245-chr11
HN873-chr11
HN51-chr11
S83-chr11
0 Mb 12 Mb 25 Mb 37 Mb 50 Mb 62 Mb 75 Mb 87 Mb 100 Mb 113 Mb 125 Mb
K30076-chr12
S245-chr12
HN873-chr12
HN51-chr12
S83-chr12
0 Mb 14 Mb 29 Mb 44 Mb 59 Mb 73 Mb 88 Mb 103 Mb 118 Mb 133 Mb 147 Mb
K30076-chr13
S245-chr13
HN873-chr13
HN51-chr13
S83-chr13
0 Mb 14 Mb 29 Mb 44 Mb 59 Mb 74 Mb 89 Mb 104 Mb 119 Mb 134 Mb 149 Mb
K30076-chr14
S245-chr14
HN873-chr14
HN51-chr14
S83-chr14
0 Mb 16 Mb 32 Mb 48 Mb 64 Mb 80 Mb 96 Mb 112 Mb 128 Mb 144 Mb 160 Mb
K30076-chr15
S245-chr15
HN873-chr15
HN51-chr15
S83-chr15
0 Mb 15 Mb 30 Mb 45 Mb 61 Mb 76 Mb 91 Mb 107 Mb 122 Mb 137 Mb 153 Mb
K30076-chr16
S245-chr16
HN873-chr16
HN51-chr16
S83-chr16
0 Mb 14 Mb 28 Mb 43 Mb 57 Mb 71 Mb 86 Mb 100 Mb 114 Mb 129 Mb 143 Mb
K30076-chr17
S245-chr17
HN873-chr17
HN51-chr17
S83-chr17
0 Mb 14 Mb 28 Mb 42 Mb 56 Mb 70 Mb 85 Mb 99 Mb 113 Mb 127 Mb 141 Mb
K30076-chr18
S245-chr18
HN873-chr18
HN51-chr18
S83-chr18
0 Mb 16 Mb 32 Mb 48 Mb 64 Mb 80 Mb 96 Mb 112 Mb 128 Mb 144 Mb 160 Mb
K30076-chr19
S245-chr19
HN873-chr19
HN51-chr19
S83-chr19
0 Mb 15 Mb 30 Mb 45 Mb 61 Mb 76 Mb 91 Mb 107 Mb 122 Mb 137 Mb 153 Mb
K30076-chr20
S245-chr20
HN873-chr20
HN51-chr20
S83-chr20

g

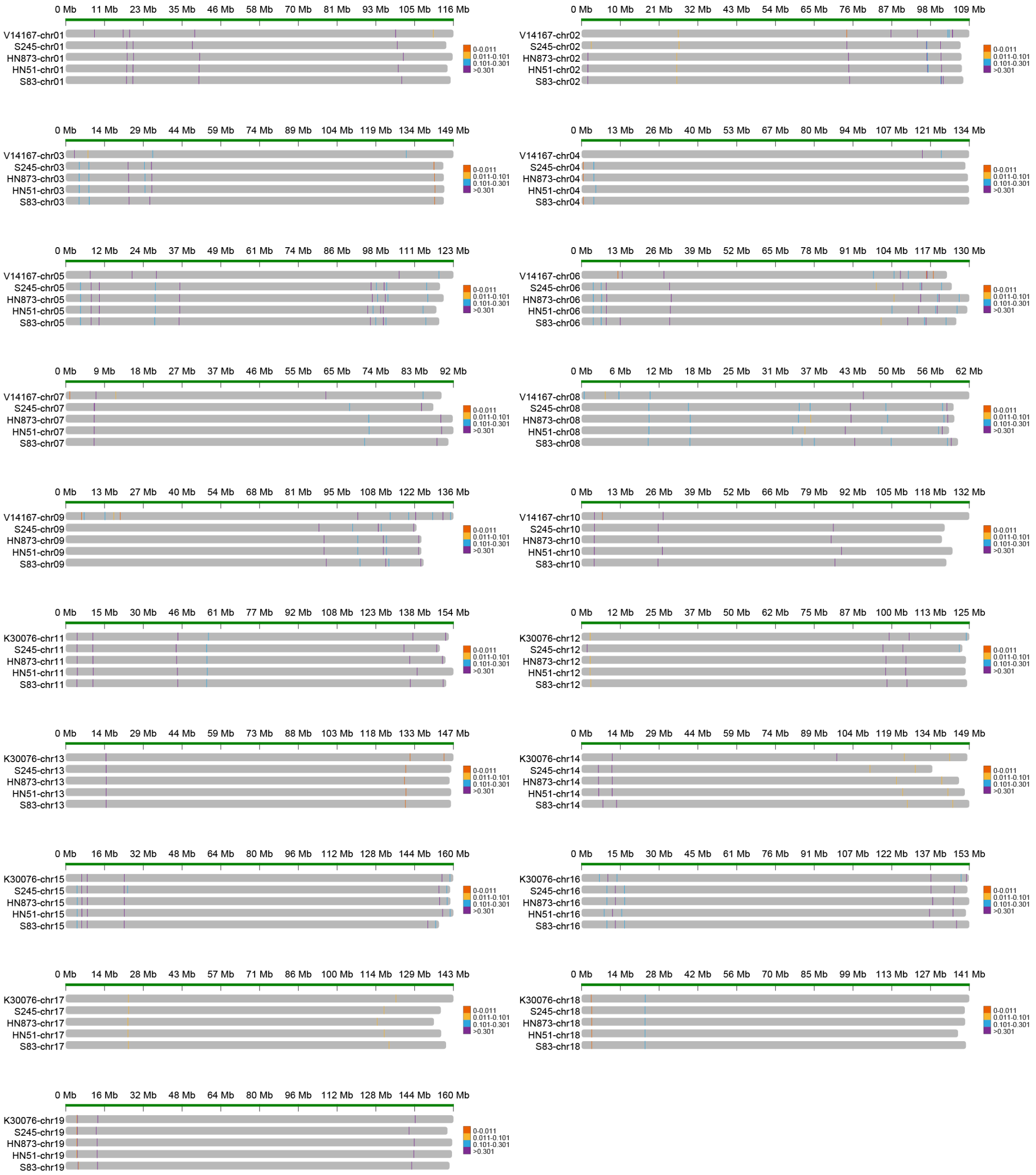

0 Mb 11 Mb 23 Mb 35 Mb 46 Mb 58 Mb 70 Mb 81 Mb 93 Mb 105 Mb 116 Mb
V14167-chr01
S245-chr01
HN873-chr01
HN51-chr01
S83-chr01
0 Mb 10 Mb 21 Mb 32 Mb 43 Mb 54 Mb 65 Mb 76 Mb 87 Mb 98 Mb 109 Mb
V14167-chr02
S245-chr02
HN873-chr02
HN51-chr02
S83-chr02
0 Mb 14 Mb 29 Mb 44 Mb 59 Mb 74 Mb 89 Mb 104 Mb 119 Mb 134 Mb 149 Mb
V14167-chr03
S245-chr03
HN873-chr03
HN51-chr03
S83-chr03
0 Mb 13 Mb 26 Mb 40 Mb 53 Mb 67 Mb 80 Mb 94 Mb 107 Mb 121 Mb 134 Mb
V14167-chr04
S245-chr04
HN873-chr04
HN51-chr04
S83-chr04
0 Mb 12 Mb 24 Mb 37 Mb 49 Mb 61 Mb 74 Mb 86 Mb 98 Mb 111 Mb 123 Mb
V14167-chr05
S245-chr05
HN873-chr05
HN51-chr05
S83-chr05
0 Mb 13 Mb 26 Mb 39 Mb 52 Mb 65 Mb 78 Mb 91 Mb 104 Mb 117 Mb 130 Mb
V14167-chr06
S245-chr06
HN873-chr06
HN51-chr06
S83-chr06
0 Mb 9 Mb 18 Mb 27 Mb 37 Mb 46 Mb 55 Mb 65 Mb 74 Mb 83 Mb 92 Mb
V14167-chr07
S245-chr07
HN873-chr07
HN51-chr07
S83-chr07
0 Mb 6 Mb 12 Mb 18 Mb 25 Mb 31 Mb 37 Mb 43 Mb 50 Mb 56 Mb 62 Mb
V14167-chr08
S245-chr08
HN873-chr08
HN51-chr08
S83-chr08
0 Mb 13 Mb 27 Mb 40 Mb 54 Mb 68 Mb 81 Mb 95 Mb 108 Mb 122 Mb 136 Mb
V14167-chr09
S245-chr09
HN873-chr09
HN51-chr09
S83-chr09
0 Mb 13 Mb 26 Mb 39 Mb 52 Mb 66 Mb 79 Mb 92 Mb 105 Mb 118 Mb 132 Mb
V14167-chr10
S245-chr10
HN873-chr10
HN51-chr10
S83-chr10
0 Mb 15 Mb 30 Mb 46 Mb 61 Mb 77 Mb 92 Mb 108 Mb 123 Mb 138 Mb 154 Mb
K30076-chr11
S245-chr11
HN873-chr11
HN51-chr11
S83-chr11
0 Mb 12 Mb 25 Mb 37 Mb 50 Mb 62 Mb 75 Mb 87 Mb 100 Mb 113 Mb 125 Mb
K30076-chr12
S245-chr12
HN873-chr12
HN51-chr12
S83-chr12
0 Mb 14 Mb 29 Mb 44 Mb 59 Mb 73 Mb 88 Mb 103 Mb 118 Mb 133 Mb 147 Mb
K30076-chr13
S245-chr13
HN873-chr13
HN51-chr13
S83-chr13
0 Mb 14 Mb 29 Mb 44 Mb 59 Mb 74 Mb 89 Mb 104 Mb 119 Mb 134 Mb 149 Mb
K30076-chr14
S245-chr14
HN873-chr14
HN51-chr14
S83-chr14
0 Mb 16 Mb 32 Mb 48 Mb 64 Mb 80 Mb 96 Mb 112 Mb 128 Mb 144 Mb 160 Mb
K30076-chr15
S245-chr15
HN873-chr15
HN51-chr15
S83-chr15
0 Mb 15 Mb 30 Mb 45 Mb 61 Mb 76 Mb 91 Mb 107 Mb 122 Mb 137 Mb 153 Mb
K30076-chr16
S245-chr16
HN873-chr16
HN51-chr16
S83-chr16
0 Mb 14 Mb 28 Mb 43 Mb 57 Mb 71 Mb 86 Mb 100 Mb 114 Mb 129 Mb 143 Mb
K30076-chr17
S245-chr17
HN873-chr17
HN51-chr17
S83-chr17
0 Mb 14 Mb 28 Mb 42 Mb 56 Mb 70 Mb 85 Mb 99 Mb 113 Mb 127 Mb 141 Mb
K30076-chr18
S245-chr18
HN873-chr18
HN51-chr18
S83-chr18
0 Mb 16 Mb 32 Mb 48 Mb 64 Mb 80 Mb 96 Mb 112 Mb 128 Mb 144 Mb 160 Mb
K30076-chr19
S245-chr19
HN873-chr19
HN51-chr19
S83-chr19
0-0.011
0.011-0.101
0.101-0.301
>0.301

h

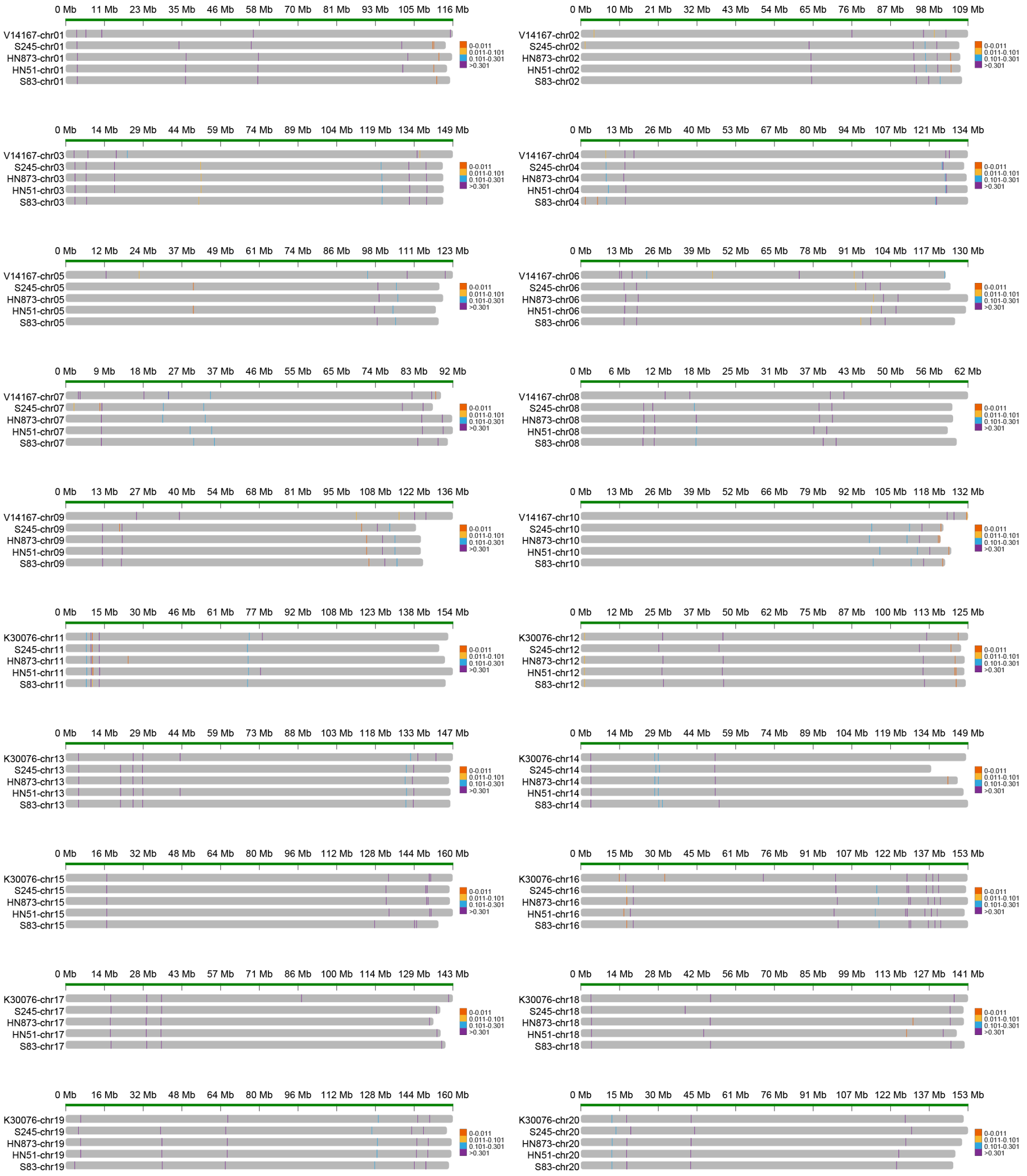

i

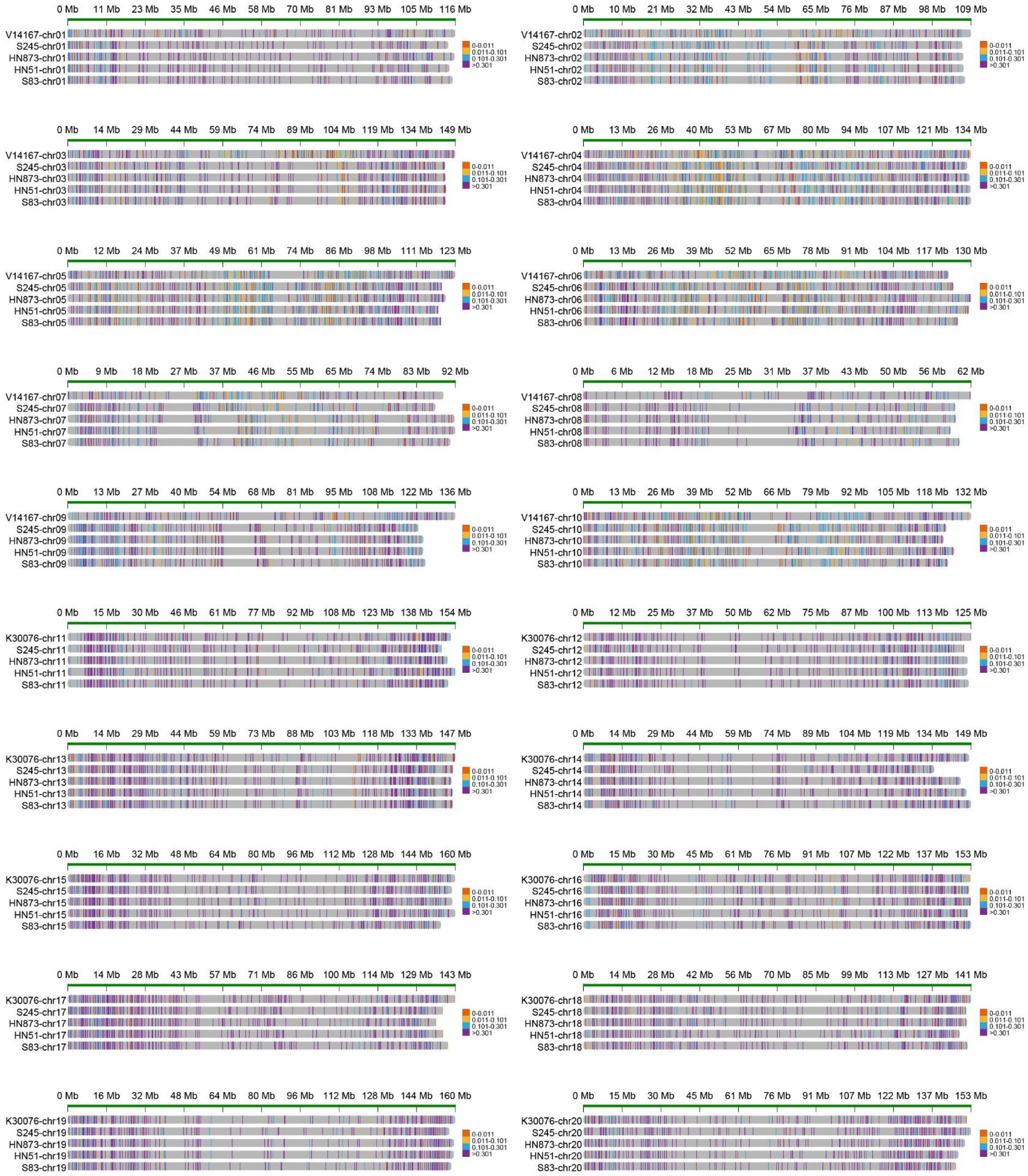
0 Mb 11 Mb 23 Mb 35 Mb 46 Mb 58 Mb 70 Mb 81 Mb 93 Mb 105 Mb 116 Mb
V14167-chr01
S245-chr01
HN873-chr01
HN51-chr01
S83-chr01
0 Mb 10 Mb 21 Mb 32 Mb 43 Mb 54 Mb 65 Mb 76 Mb 87 Mb 98 Mb 109 Mb
V14167-chr02
S245-chr02
HN873-chr02
HN51-chr02
S83-chr02
0 Mb 14 Mb 29 Mb 44 Mb 59 Mb 74 Mb 89 Mb 104 Mb 119 Mb 134 Mb 149 Mb
V14167-chr03
S245-chr03
HN873-chr03
HN51-chr03
S83-chr03
0 Mb 13 Mb 26 Mb 40 Mb 53 Mb 67 Mb 80 Mb 94 Mb 107 Mb 121 Mb 134 Mb
V14167-chr04
S245-chr04
HN873-chr04
HN51-chr04
S83-chr04
0 Mb 12 Mb 24 Mb 37 Mb 49 Mb 61 Mb 74 Mb 86 Mb 98 Mb 111 Mb 123 Mb
V14167-chr05
S245-chr05
HN873-chr05
HN51-chr05
S83-chr05
0 Mb 13 Mb 26 Mb 39 Mb 52 Mb 65 Mb 78 Mb 91 Mb 104 Mb 117 Mb 130 Mb
V14167-chr06
S245-chr06
HN873-chr06
HN51-chr06
S83-chr06
0 Mb 9 Mb 18 Mb 27 Mb 37 Mb 46 Mb 55 Mb 65 Mb 74 Mb 83 Mb 92 Mb
V14167-chr07
S245-chr07
HN873-chr07
HN51-chr07
S83-chr07
0 Mb 6 Mb 12 Mb 18 Mb 25 Mb 31 Mb 37 Mb 43 Mb 50 Mb 56 Mb 62 Mb
V14167-chr08
S245-chr08
HN873-chr08
HN51-chr08
S83-chr08
0 Mb 13 Mb 27 Mb 40 Mb 54 Mb 68 Mb 81 Mb 95 Mb 108 Mb 122 Mb 136 Mb
V14167-chr09
S245-chr09
HN873-chr09
HN51-chr09
S83-chr09
0 Mb 13 Mb 26 Mb 39 Mb 52 Mb 66 Mb 79 Mb 92 Mb 105 Mb 118 Mb 132 Mb
V14167-chr10
S245-chr10
HN873-chr10
HN51-chr10
S83-chr10
0 Mb 15 Mb 30 Mb 46 Mb 61 Mb 77 Mb 92 Mb 108 Mb 123 Mb 138 Mb 154 Mb
K30076-chr11
S245-chr11
HN873-chr11
HN51-chr11
S83-chr11
0 Mb 12 Mb 25 Mb 37 Mb 50 Mb 62 Mb 75 Mb 87 Mb 100 Mb 113 Mb 125 Mb
K30076-chr12
S245-chr12
HN873-chr12
HN51-chr12
S83-chr12
0 Mb 14 Mb 29 Mb 44 Mb 59 Mb 73 Mb 88 Mb 103 Mb 118 Mb 133 Mb 147 Mb
K30076-chr13
S245-chr13
HN873-chr13
HN51-chr13
S83-chr13
0 Mb 14 Mb 29 Mb 44 Mb 59 Mb 74 Mb 89 Mb 104 Mb 119 Mb 134 Mb 149 Mb
K30076-chr14
S245-chr14
HN873-chr14
HN51-chr14
S83-chr14
0 Mb 16 Mb 32 Mb 48 Mb 64 Mb 80 Mb 96 Mb 112 Mb 128 Mb 144 Mb 160 Mb
K30076-chr15
S245-chr15
HN873-chr15
HN51-chr15
S83-chr15
0 Mb 15 Mb 30 Mb 45 Mb 61 Mb 76 Mb 91 Mb 107 Mb 122 Mb 137 Mb 153 Mb
K30076-chr16
S245-chr16
HN873-chr16
HN51-chr16
S83-chr16
0 Mb 14 Mb 28 Mb 43 Mb 57 Mb 71 Mb 86 Mb 100 Mb 114 Mb 129 Mb 143 Mb
K30076-chr17
S245-chr17
HN873-chr17
HN51-chr17
S83-chr17
0 Mb 14 Mb 28 Mb 42 Mb 56 Mb 70 Mb 85 Mb 99 Mb 113 Mb 127 Mb 141 Mb
K30076-chr18
S245-chr18
HN873-chr18
HN51-chr18
S83-chr18
0 Mb 16 Mb 32 Mb 48 Mb 64 Mb 80 Mb 96 Mb 112 Mb 128 Mb 144 Mb 160 Mb
K30076-chr19
S245-chr19
HN873-chr19
HN51-chr19
S83-chr19
0 Mb 15 Mb 30 Mb 45 Mb 61 Mb 76 Mb 91 Mb 107 Mb 122 Mb 137 Mb 153 Mb
K30076-chr20
S245-chr20
HN873-chr20
HN51-chr20
S83-chr20
0-0.011
0.011-0.101
0.101-0.301
>0.301

j

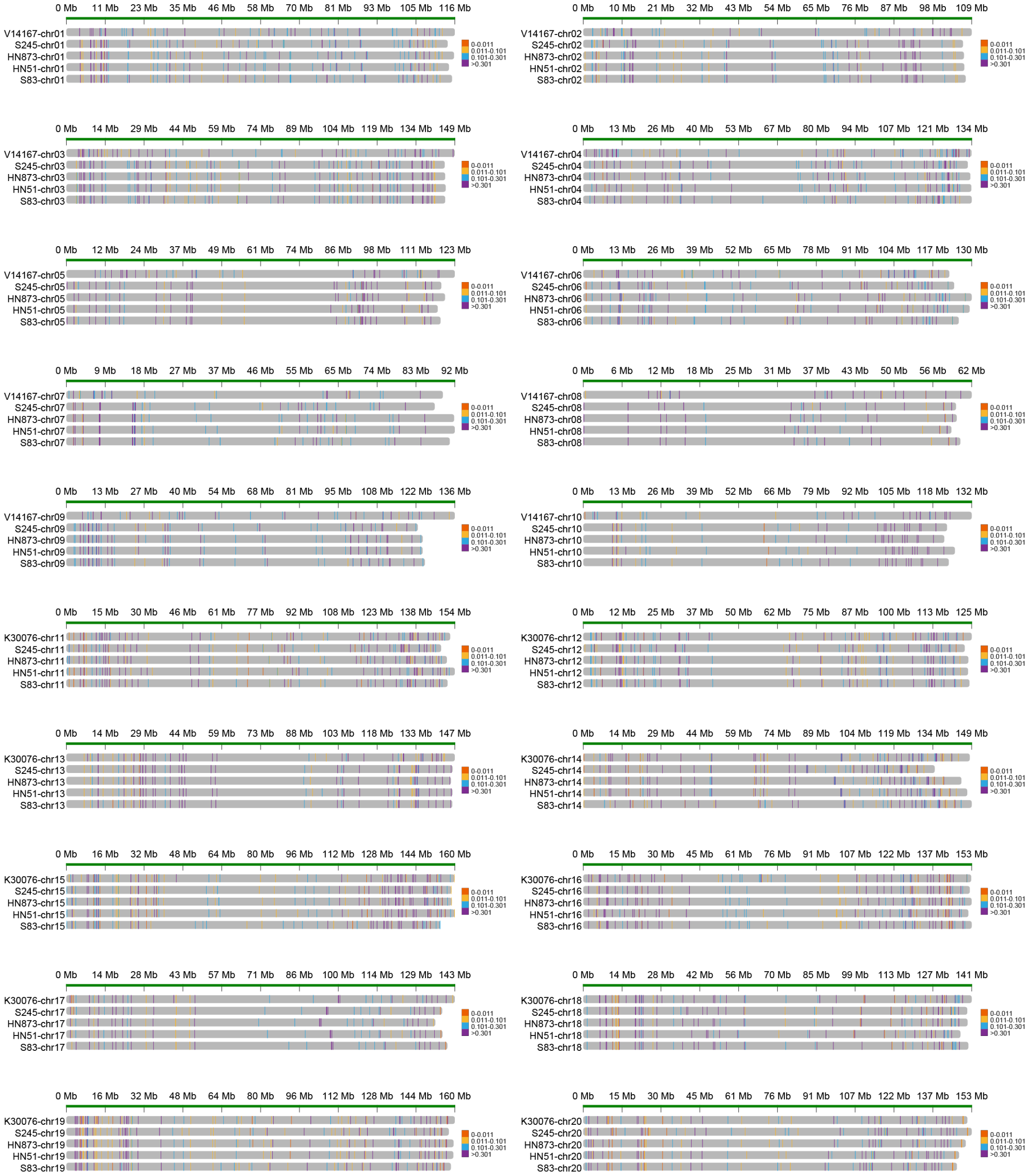

0 Mb 11 Mb 23 Mb 35 Mb 46 Mb 58 Mb 70 Mb 81 Mb 93 Mb 105 Mb 116 Mb
V14167-chr01
S245-chr01
HN873-chr01
HN51-chr01
S83-chr01
0 Mb 10 Mb 21 Mb 32 Mb 43 Mb 54 Mb 65 Mb 76 Mb 87 Mb 98 Mb 109 Mb
V14167-chr02
S245-chr02
HN873-chr02
HN51-chr02
S83-chr02
0 Mb 14 Mb 29 Mb 44 Mb 59 Mb 74 Mb 89 Mb 104 Mb 119 Mb 134 Mb 149 Mb
V14167-chr03
S245-chr03
HN873-chr03
HN51-chr03
S83-chr03
0 Mb 13 Mb 26 Mb 40 Mb 53 Mb 67 Mb 80 Mb 94 Mb 107 Mb 121 Mb 134 Mb
V14167-chr04
S245-chr04
HN873-chr04
HN51-chr04
S83-chr04
0 Mb 12 Mb 24 Mb 37 Mb 49 Mb 61 Mb 74 Mb 86 Mb 98 Mb 111 Mb 123 Mb
V14167-chr05
S245-chr05
HN873-chr05
HN51-chr05
S83-chr05
0 Mb 13 Mb 26 Mb 39 Mb 52 Mb 65 Mb 78 Mb 91 Mb 104 Mb 117 Mb 130 Mb
V14167-chr06
S245-chr06
HN873-chr06
HN51-chr06
S83-chr06
0 Mb 9 Mb 18 Mb 27 Mb 37 Mb 46 Mb 55 Mb 65 Mb 74 Mb 83 Mb 92 Mb
V14167-chr07
S245-chr07
HN873-chr07
HN51-chr07
S83-chr07
0 Mb 6 Mb 12 Mb 18 Mb 25 Mb 31 Mb 37 Mb 43 Mb 50 Mb 56 Mb 62 Mb
V14167-chr08
S245-chr08
HN873-chr08
HN51-chr08
S83-chr08
0 Mb 13 Mb 27 Mb 40 Mb 54 Mb 68 Mb 81 Mb 95 Mb 108 Mb 122 Mb 136 Mb
V14167-chr09
S245-chr09
HN873-chr09
HN51-chr09
S83-chr09
0 Mb 13 Mb 26 Mb 39 Mb 52 Mb 66 Mb 79 Mb 92 Mb 105 Mb 118 Mb 132 Mb
V14167-chr10
S245-chr10
HN873-chr10
HN51-chr10
S83-chr10
0 Mb 15 Mb 30 Mb 46 Mb 61 Mb 77 Mb 92 Mb 108 Mb 123 Mb 138 Mb 154 Mb
K30076-chr11
S245-chr11
HN873-chr11
HN51-chr11
S83-chr11
0 Mb 12 Mb 25 Mb 37 Mb 50 Mb 62 Mb 75 Mb 87 Mb 100 Mb 113 Mb 125 Mb
K30076-chr12
S245-chr12
HN873-chr12
HN51-chr12
S83-chr12
0 Mb 14 Mb 29 Mb 44 Mb 59 Mb 73 Mb 88 Mb 103 Mb 118 Mb 133 Mb 147 Mb
K30076-chr13
S245-chr13
HN873-chr13
HN51-chr13
S83-chr13
0 Mb 14 Mb 29 Mb 44 Mb 59 Mb 74 Mb 89 Mb 104 Mb 119 Mb 134 Mb 149 Mb
K30076-chr14
S245-chr14
HN873-chr14
HN51-chr14
S83-chr14
0 Mb 16 Mb 32 Mb 48 Mb 64 Mb 80 Mb 96 Mb 112 Mb 128 Mb 144 Mb 160 Mb
K30076-chr15
S245-chr15
HN873-chr15
HN51-chr15
S83-chr15
0 Mb 15 Mb 30 Mb 45 Mb 61 Mb 76 Mb 91 Mb 107 Mb 122 Mb 137 Mb 153 Mb
K30076-chr16
S245-chr16
HN873-chr16
HN51-chr16
S83-chr16
0 Mb 14 Mb 28 Mb 43 Mb 57 Mb 71 Mb 86 Mb 100 Mb 114 Mb 129 Mb 143 Mb
K30076-chr17
S245-chr17
HN873-chr17
HN51-chr17
S83-chr17
0 Mb 14 Mb 28 Mb 42 Mb 56 Mb 70 Mb 85 Mb 99 Mb 113 Mb 127 Mb 141 Mb
K30076-chr18
S245-chr18
HN873-chr18
HN51-chr18
S83-chr18
0 Mb 16 Mb 32 Mb 48 Mb 64 Mb 80 Mb 96 Mb 112 Mb 128 Mb 144 Mb 160 Mb
K30076-chr19
S245-chr19
HN873-chr19
HN51-chr19
S83-chr19
0 Mb 15 Mb 30 Mb 45 Mb 61 Mb 76 Mb 91 Mb 107 Mb 122 Mb 137 Mb 153 Mb
K30076-chr20
S245-chr20
HN873-chr20
HN51-chr20
S83-chr20
0-0.011
0.011-0.101
0.101-0.301
>0.301

k

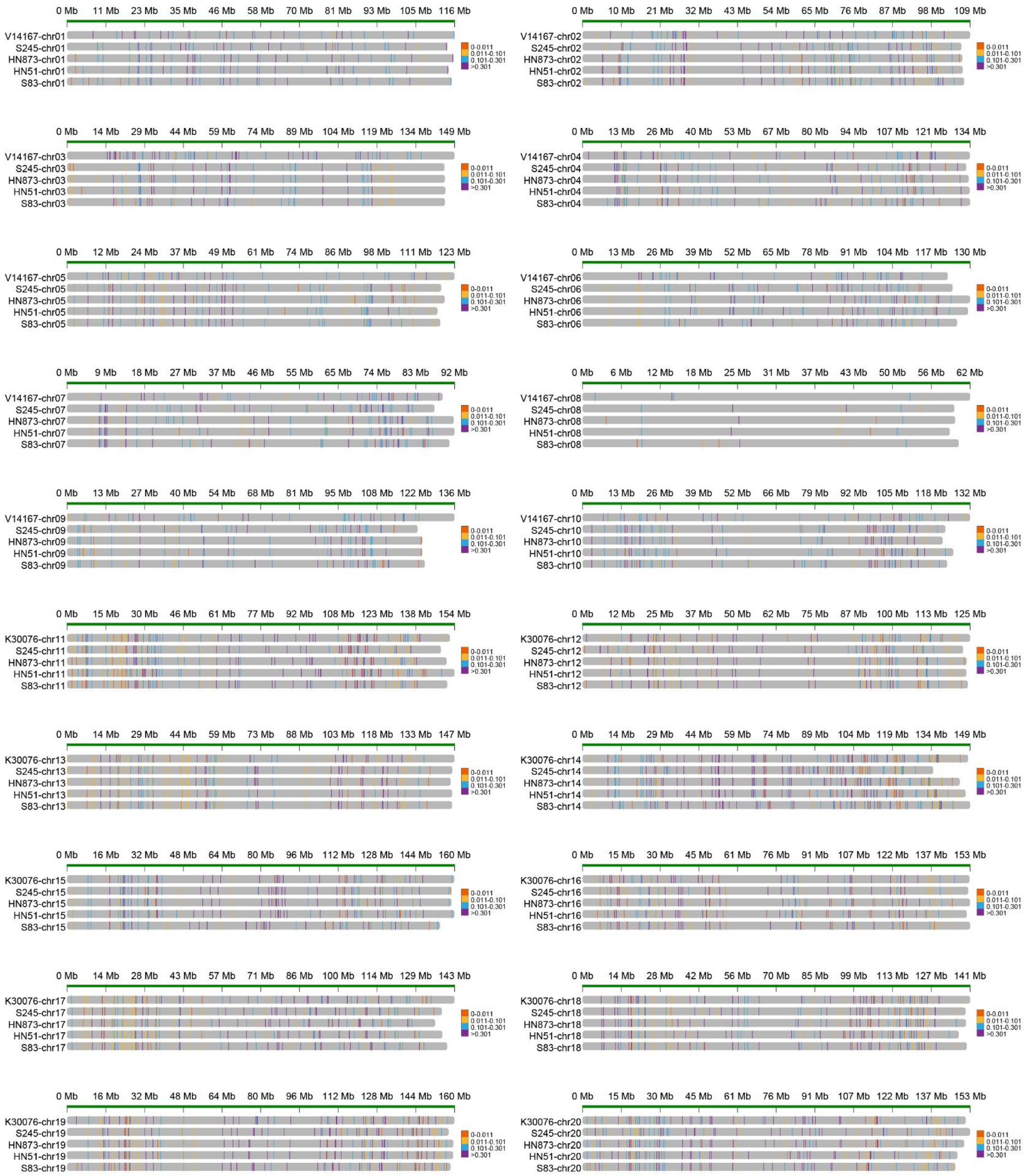
0 Mb 11 Mb 23 Mb 35 Mb 46 Mb 58 Mb 70 Mb 81 Mb 93 Mb 105 Mb 116 Mb
V14167-chr01
S245-chr01
HN873-chr01
HN51-chr01
S83-chr01
0-0.011
0.011-0.101
0.101-0.301
>0.301
0 Mb 10 Mb 21 Mb 32 Mb 43 Mb 54 Mb 65 Mb 76 Mb 87 Mb 98 Mb 109 Mb
V14167-chr02
S245-chr02
HN873-chr02
HN51-chr02
S83-chr02
0 Mb 14 Mb 29 Mb 44 Mb 59 Mb 74 Mb 89 Mb 104 Mb 119 Mb 134 Mb 149 Mb
V14167-chr03
S245-chr03
HN873-chr03
HN51-chr03
S83-chr03
0 Mb 13 Mb 26 Mb 40 Mb 53 Mb 67 Mb 80 Mb 94 Mb 107 Mb 121 Mb 134 Mb
V14167-chr04
S245-chr04
HN873-chr04
HN51-chr04
S83-chr04
0 Mb 12 Mb 24 Mb 37 Mb 49 Mb 61 Mb 74 Mb 86 Mb 98 Mb 111 Mb 123 Mb
V14167-chr05
S245-chr05
HN873-chr05
HN51-chr05
S83-chr05
0 Mb 13 Mb 26 Mb 39 Mb 52 Mb 65 Mb 78 Mb 91 Mb 104 Mb 117 Mb 130 Mb
V14167-chr06
S245-chr06
HN873-chr06
HN51-chr06
S83-chr06
0 Mb 9 Mb 18 Mb 27 Mb 37 Mb 46 Mb 55 Mb 65 Mb 74 Mb 83 Mb 92 Mb
V14167-chr07
S245-chr07
HN873-chr07
HN51-chr07
S83-chr07
0 Mb 6 Mb 12 Mb 18 Mb 25 Mb 31 Mb 37 Mb 43 Mb 50 Mb 56 Mb 62 Mb
V14167-chr08
S245-chr08
HN873-chr08
HN51-chr08
S83-chr08
0 Mb 13 Mb 27 Mb 40 Mb 54 Mb 68 Mb 81 Mb 95 Mb 108 Mb 122 Mb 136 Mb
V14167-chr09
S245-chr09
HN873-chr09
HN51-chr09
S83-chr09
0 Mb 13 Mb 26 Mb 39 Mb 52 Mb 66 Mb 79 Mb 92 Mb 105 Mb 118 Mb 132 Mb
V14167-chr10
S245-chr10
HN873-chr10
HN51-chr10
S83-chr10
0 Mb 15 Mb 30 Mb 46 Mb 61 Mb 77 Mb 92 Mb 108 Mb 123 Mb 138 Mb 154 Mb
K30076-chr11
S245-chr11
HN873-chr11
HN51-chr11
S83-chr11
0 Mb 12 Mb 25 Mb 37 Mb 50 Mb 62 Mb 75 Mb 87 Mb 100 Mb 113 Mb 125 Mb
K30076-chr12
S245-chr12
HN873-chr12
HN51-chr12
S83-chr12
0 Mb 14 Mb 29 Mb 44 Mb 59 Mb 73 Mb 88 Mb 103 Mb 118 Mb 133 Mb 147 Mb
K30076-chr13
S245-chr13
HN873-chr13
HN51-chr13
S83-chr13
0 Mb 14 Mb 29 Mb 44 Mb 59 Mb 74 Mb 89 Mb 104 Mb 119 Mb 134 Mb 149 Mb
K30076-chr14
S245-chr14
HN873-chr14
HN51-chr14
S83-chr14
0 Mb 16 Mb 32 Mb 48 Mb 64 Mb 80 Mb 96 Mb 112 Mb 128 Mb 144 Mb 160 Mb
K30076-chr15
S245-chr15
HN873-chr15
HN51-chr15
S83-chr15
0 Mb 15 Mb 30 Mb 45 Mb 61 Mb 76 Mb 91 Mb 107 Mb 122 Mb 137 Mb 153 Mb
K30076-chr16
S245-chr16
HN873-chr16
HN51-chr16
S83-chr16
0 Mb 14 Mb 28 Mb 43 Mb 57 Mb 71 Mb 86 Mb 100 Mb 114 Mb 129 Mb 143 Mb
K30076-chr17
S245-chr17
HN873-chr17
HN51-chr17
S83-chr17
0 Mb 14 Mb 28 Mb 42 Mb 56 Mb 70 Mb 85 Mb 99 Mb 113 Mb 127 Mb 141 Mb
K30076-chr18
S245-chr18
HN873-chr18
HN51-chr18
S83-chr18
0 Mb 16 Mb 32 Mb 48 Mb 64 Mb 80 Mb 96 Mb 112 Mb 128 Mb 144 Mb 160 Mb
K30076-chr19
S245-chr19
HN873-chr19
HN51-chr19
S83-chr19
0 Mb 15 Mb 30 Mb 45 Mb 61 Mb 76 Mb 91 Mb 107 Mb 122 Mb 137 Mb 153 Mb
K30076-chr20
S245-chr20
HN873-chr20
HN51-chr20
S83-chr20

l

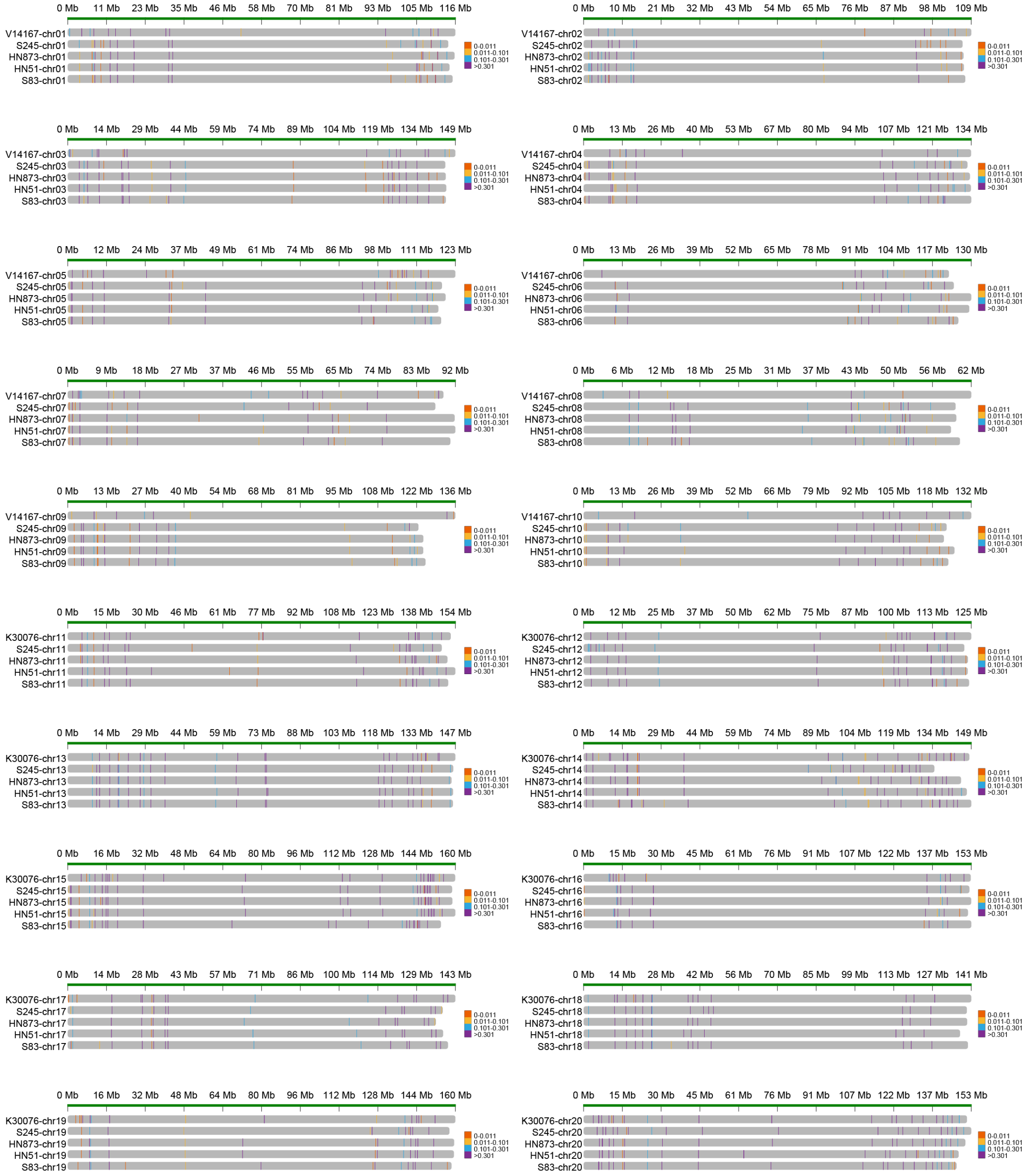

Supplementary Fig. 5 Different insertion times of *Gypsy* and *Copia*-type LTRs in different chromosomes. LTRs were divided into 12 subfamilies, including: **a,** *Ale*; **b,** *Athila*, **c,** *Bianca*; **d,** *CRM*; **e,** *Galadriel*; **f,** *Ikeros*; **g,** *Ivana*; **h,** *Reina*; **i,** *Retand*; **j,** *SIRE*; **k,** *Tekay*; **l,** *Tork*. The insertion times (Mya) are represented by different color.

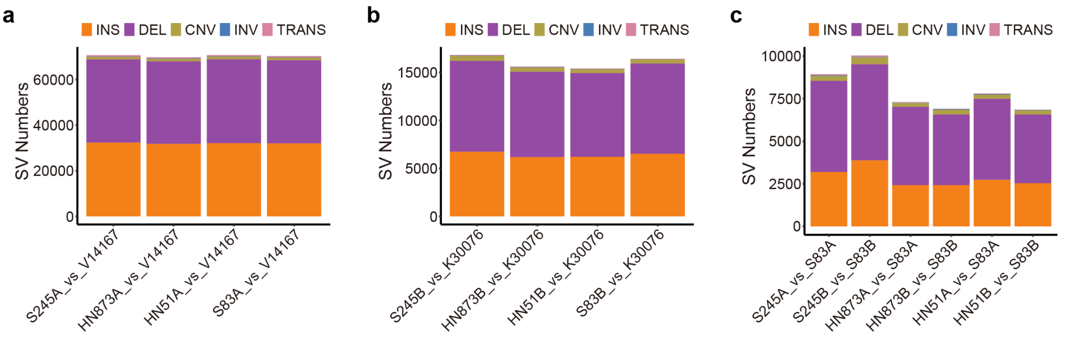

Supplementary Fig. 6 Structural Variations Among Peanut Genomes. a-c, The number of different types of structural variants present in each genome compared to K30076 (**a**), V14167 (**b**), and S83 (**c**), respectively. The types of structural variants include: INS (Insertion), DEL (Deletion), CNV (Copy Number Variation), INV (Inversion), and TRANS (Translocation).

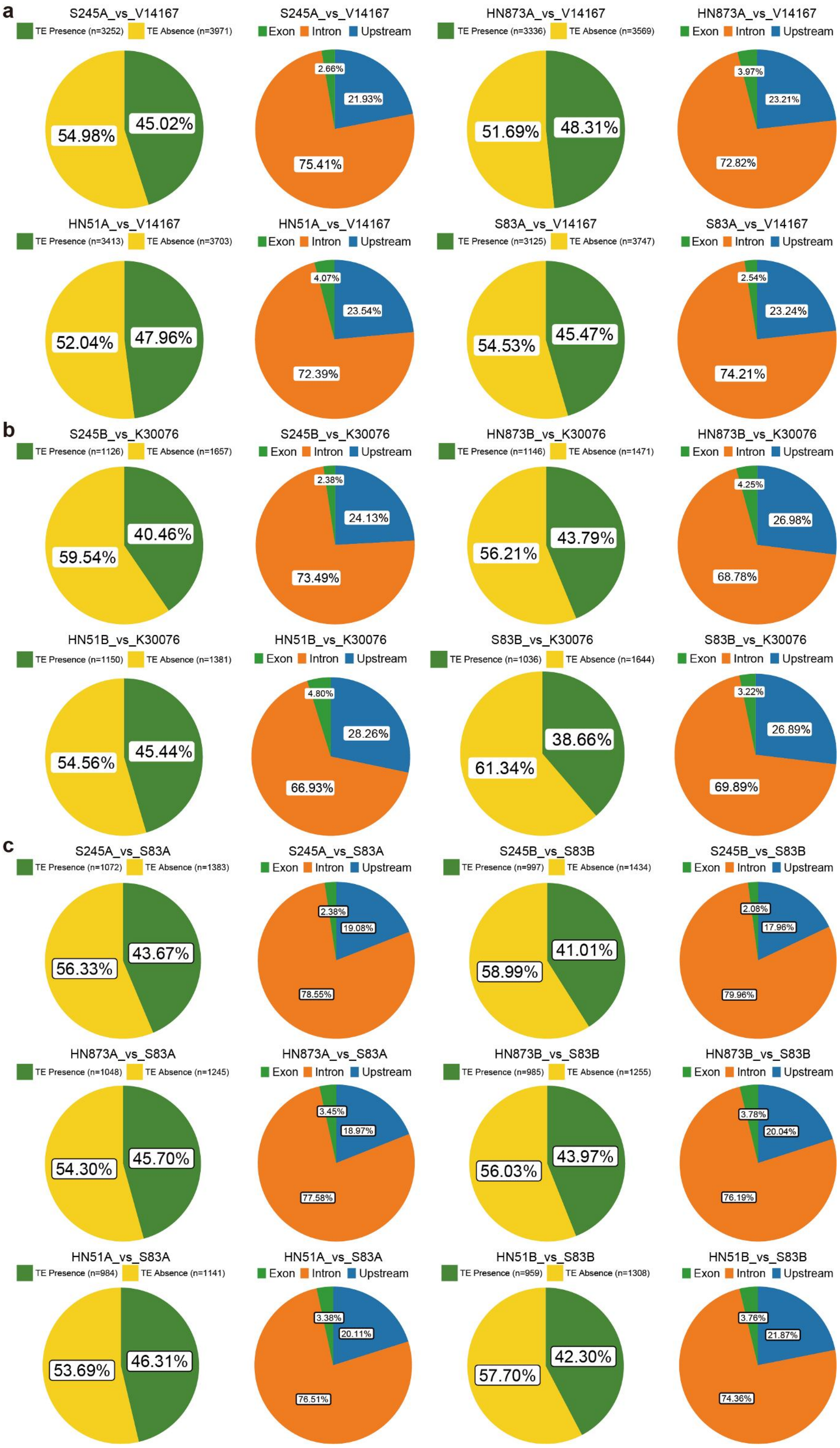

Supplementary Fig. 7 Transposable elements (TEs) and their association with structural variations (SVs) based on the peanut genome. The proportions of SV-genes matched to TEs (on the left), and the percentage of TEs in TE-SV-genes located within 1kb upstream of genes, introns, and exons. **a,** At vs Ad (V14167); **b,** Bt vs Bd (K30076); **c,** At vs At (S83) and Bt vs Bt (S83).

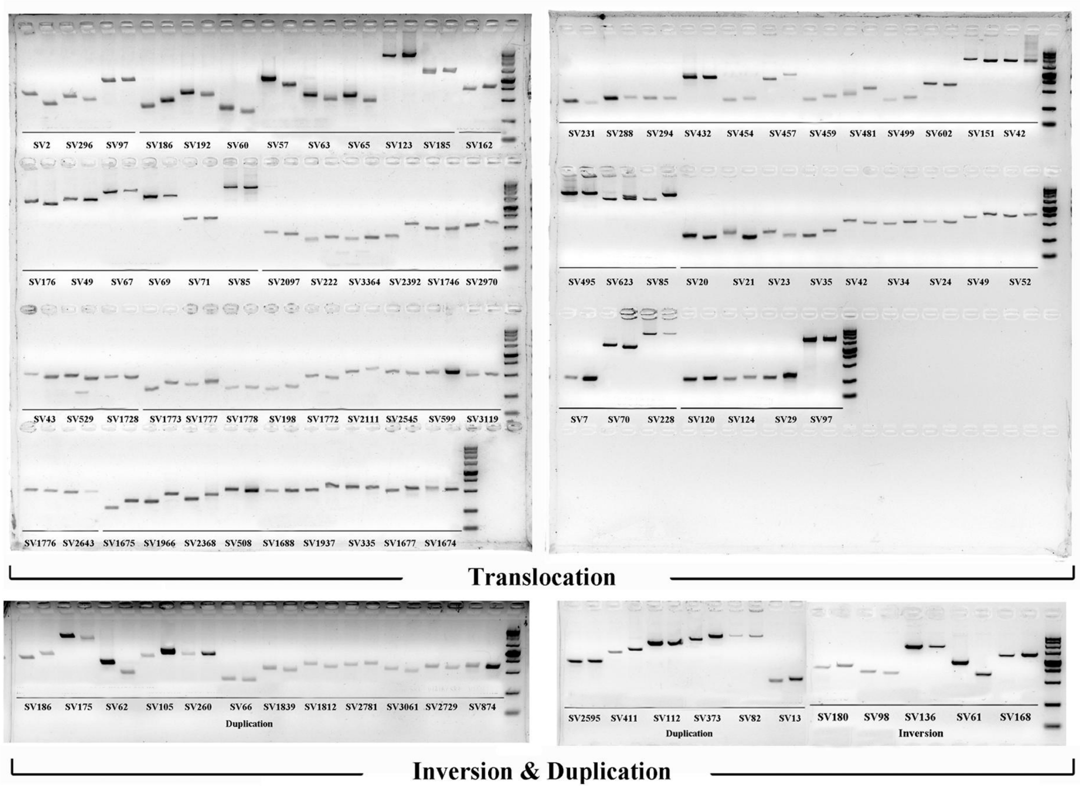

Supplementary Fig. 8 PCR validations for translocations, inversions, and duplications identified among different genomes. Amplified fragments were obtained using primers designed based on the sequence of SV sites within 2 kb upstream and downstream.

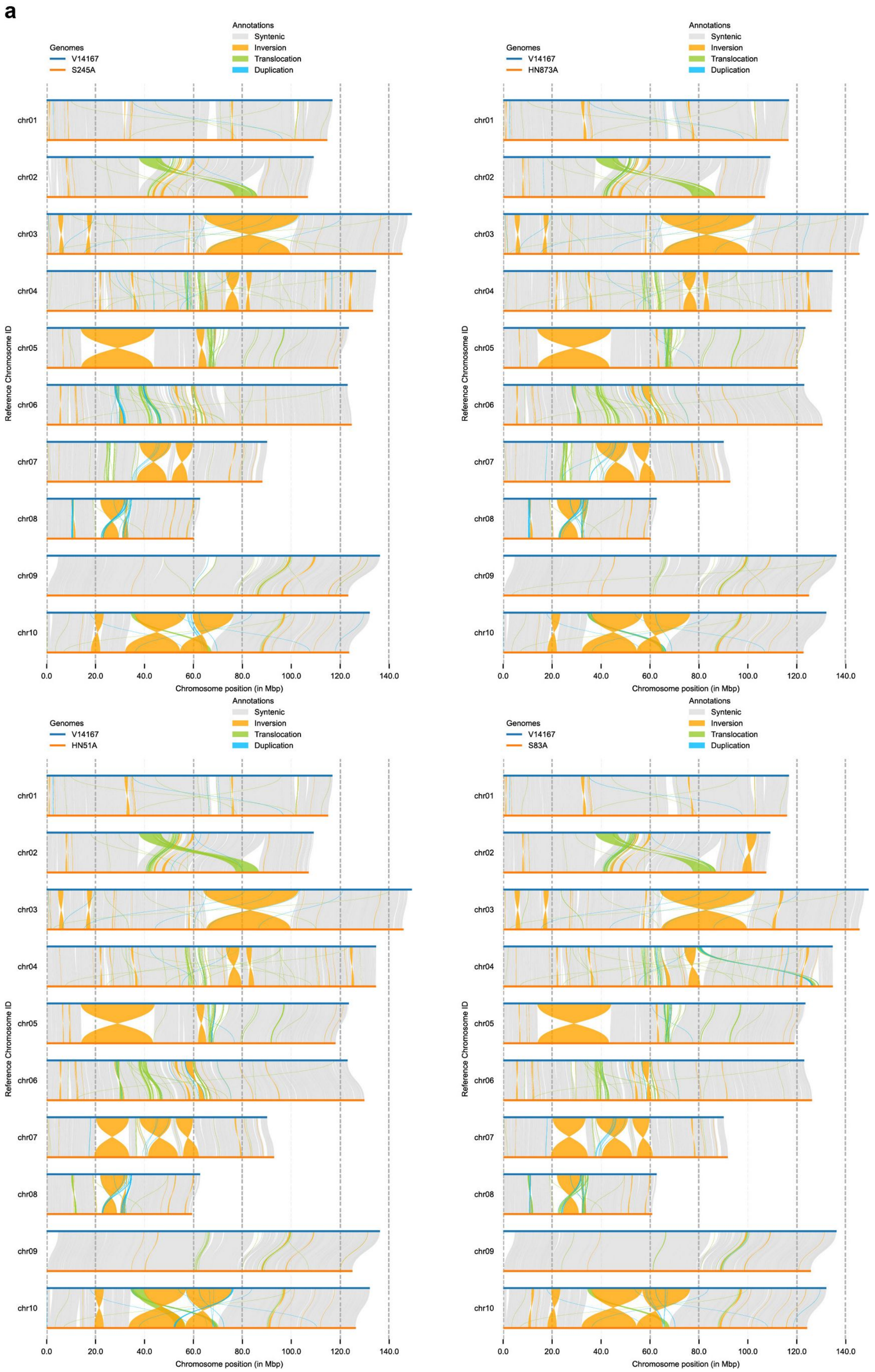
a
Annotations
Syntenic
Inversion
Translocation
Duplication
Genomes
V14167
S245A
HN873A
HN51A
S83A
chr01
chr02
chr03
chr04
chr05
chr06
chr07
chr08
chr09
chr10
Reference Chromosome ID
0.0
20.0
40.0
60.0
80.0
100.0
120.0
140.0
Chromosome position (in Mbp)

b

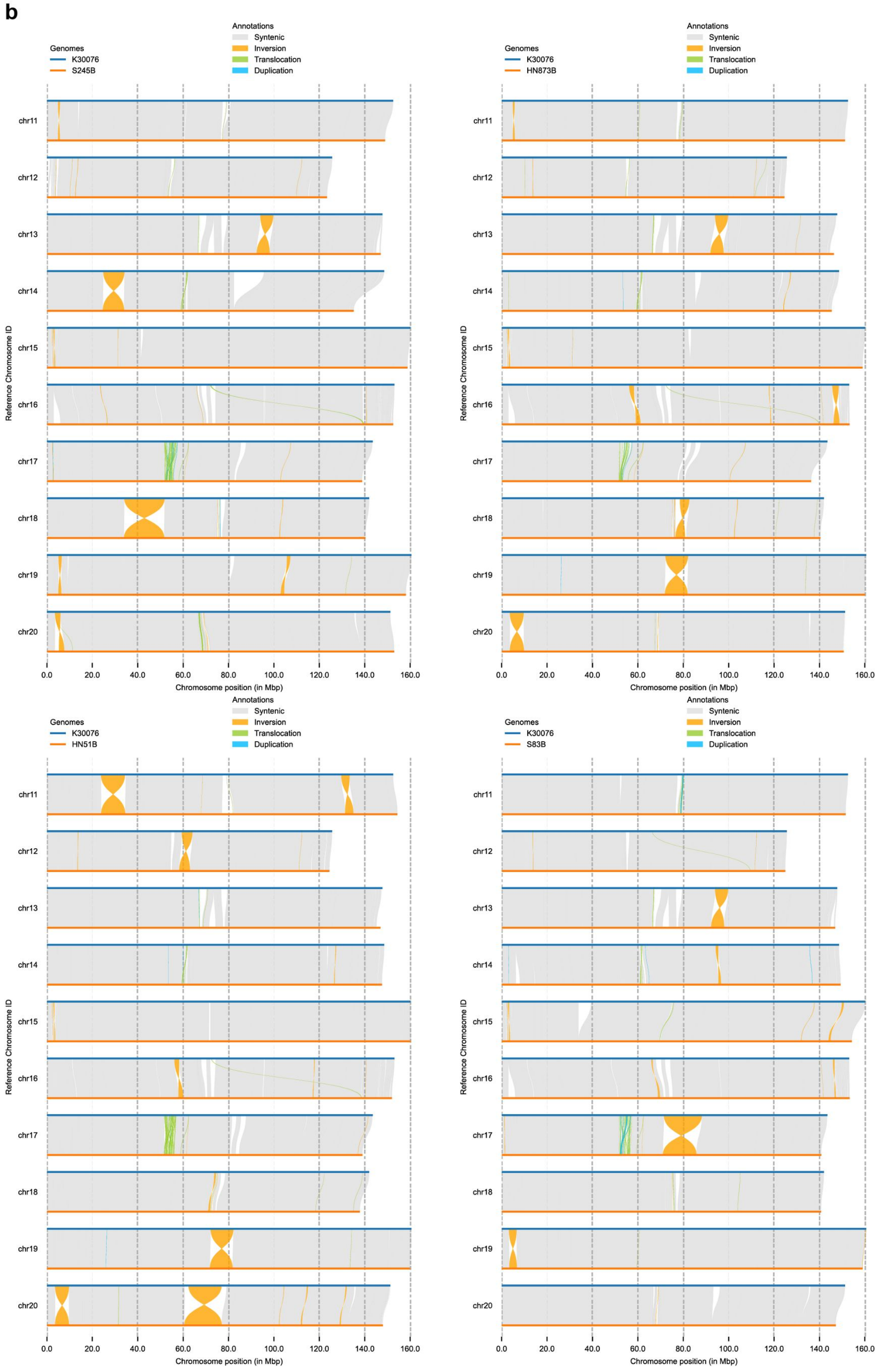

Genomes
K30076
S245B
HN873B
HN51B
S83B
Annotations
Syntenic
Inversion
Translocation
Duplication
Reference Chromosome ID
chr11
chr12
chr13
chr14
chr15
chr16
chr17
chr18
chr19
chr20
0.0
20.0
40.0
60.0
80.0
100.0
120.0
140.0
160.0
Chromosome position (in Mbp)

c

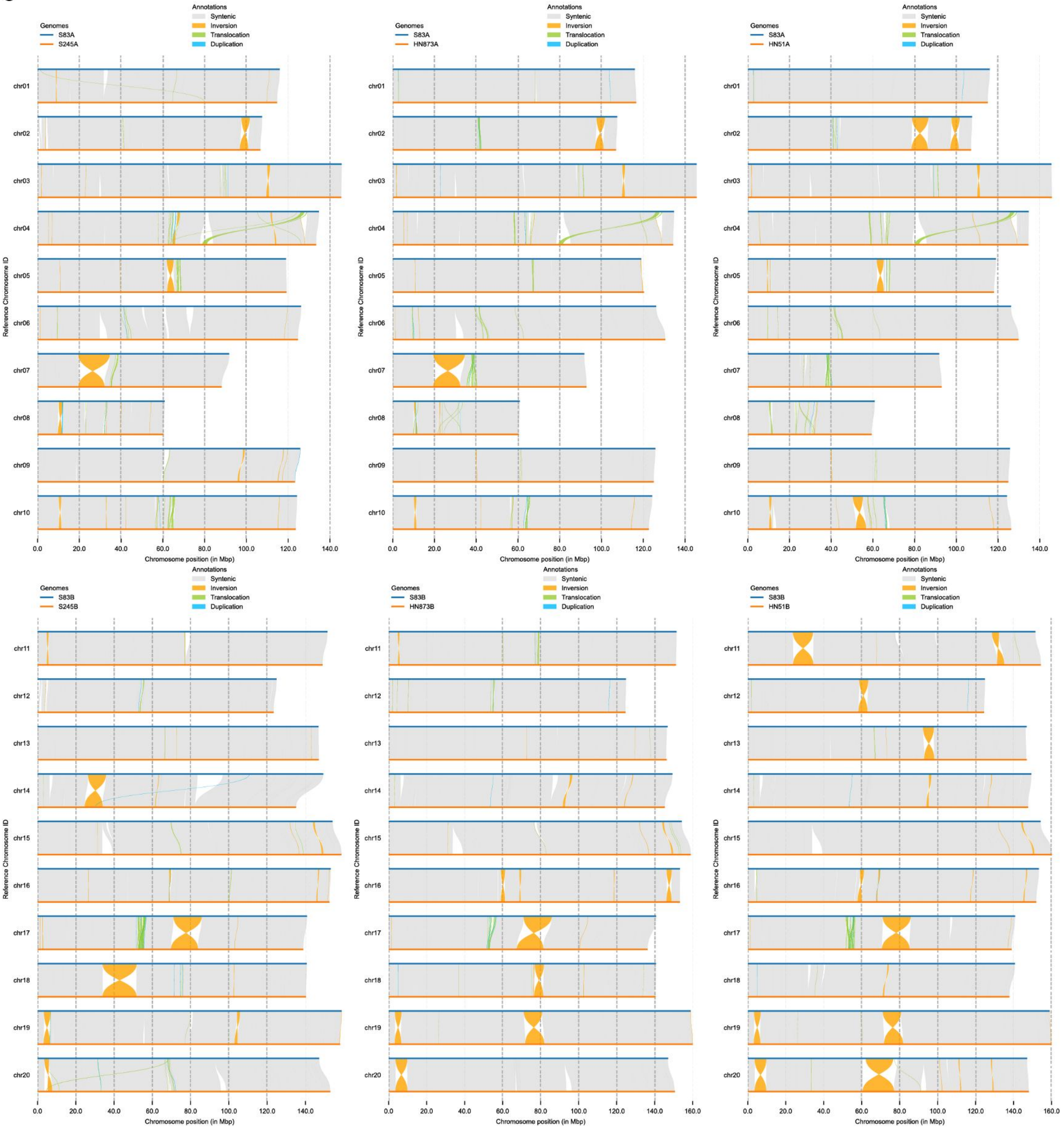

Supplementary Fig. 9 Collinearity analysis among peanut assemblies. a, Genome collinearity of V14167 (AA) genome and At subgenomes, including S245A-vs- V14167, HN873A-vs-V14167, HN51A-vs-V14167 and S83A-vs-V14167; V14167 as reference. **b,** Genome collinearity of K30076 (BB) genome and Bt subgenomes; S245B-vs-K30076, HN873B-vs-K30076, HN51B-vs-K30076 and S83B-vs-K30076; K30076 as reference. **c,** Genome collinearity among tetraploid peanuts, including: S245A-vs-S83A, HN51A-vs-S83A, HN873A-vs-S83A and S245B-vs-S83B, HN51B-vs-S83B, HN873B-vs-S83B, S83A and S83B as reference.

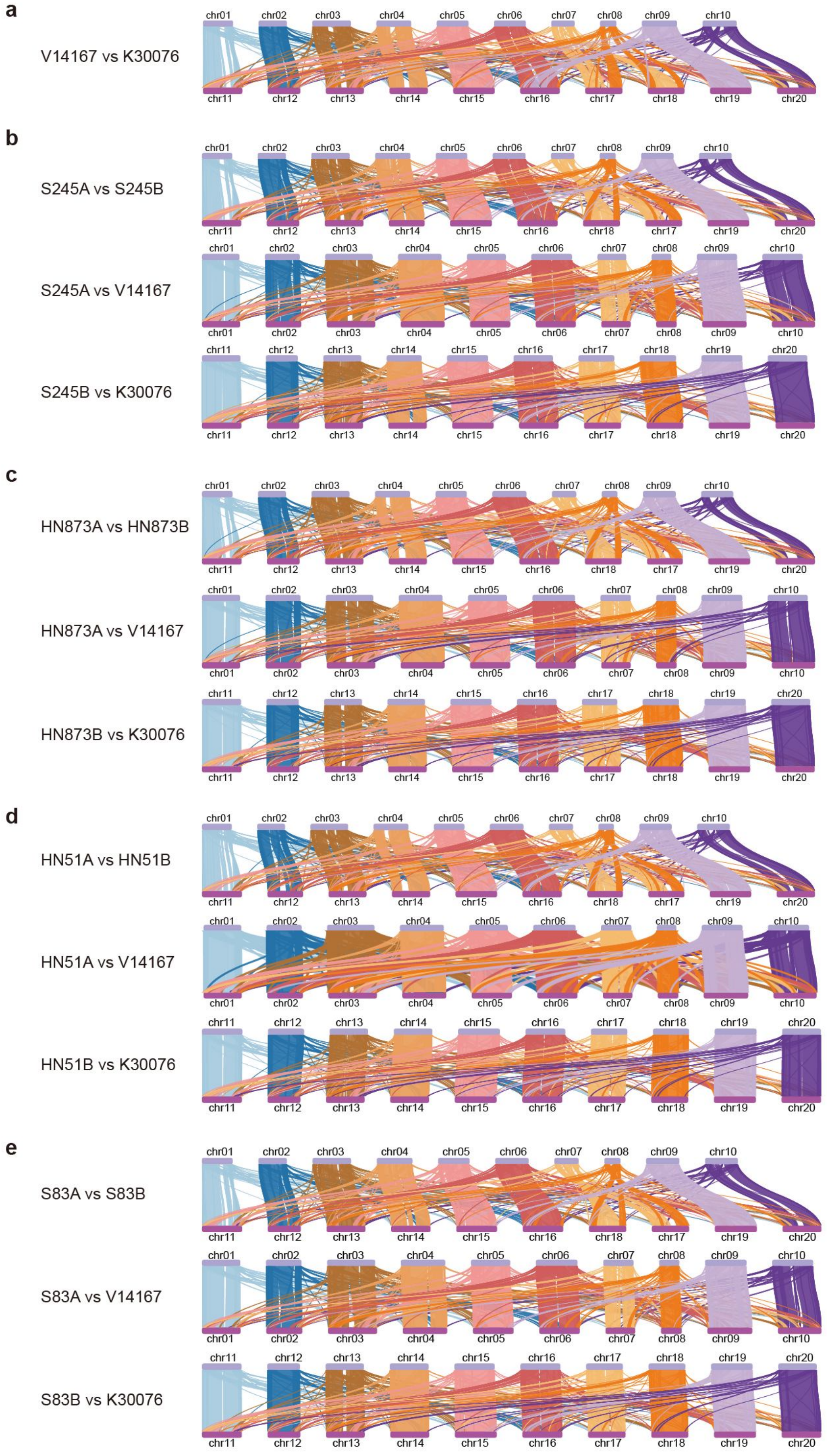

Supplementary Fig. 10 Syntenic analyses between Ad vs Bd, At vs Ad and Bt vs Bd. a, Comparison between diploid genomes Ad and Bd. **b-e,** Synteny analysis of the At and Bt subgenomes of *A.hypogaea* and their corresponding ancestral diploids. Homeologous blocks of ≥ 5 gene pairs between Chr01–Chr10 and Chr11–Chr20 are connected with lines.

a

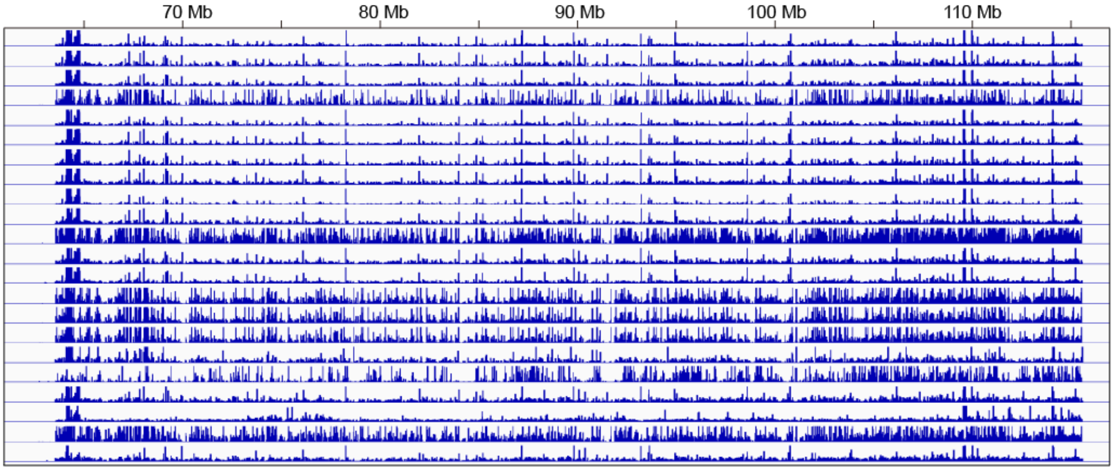

b

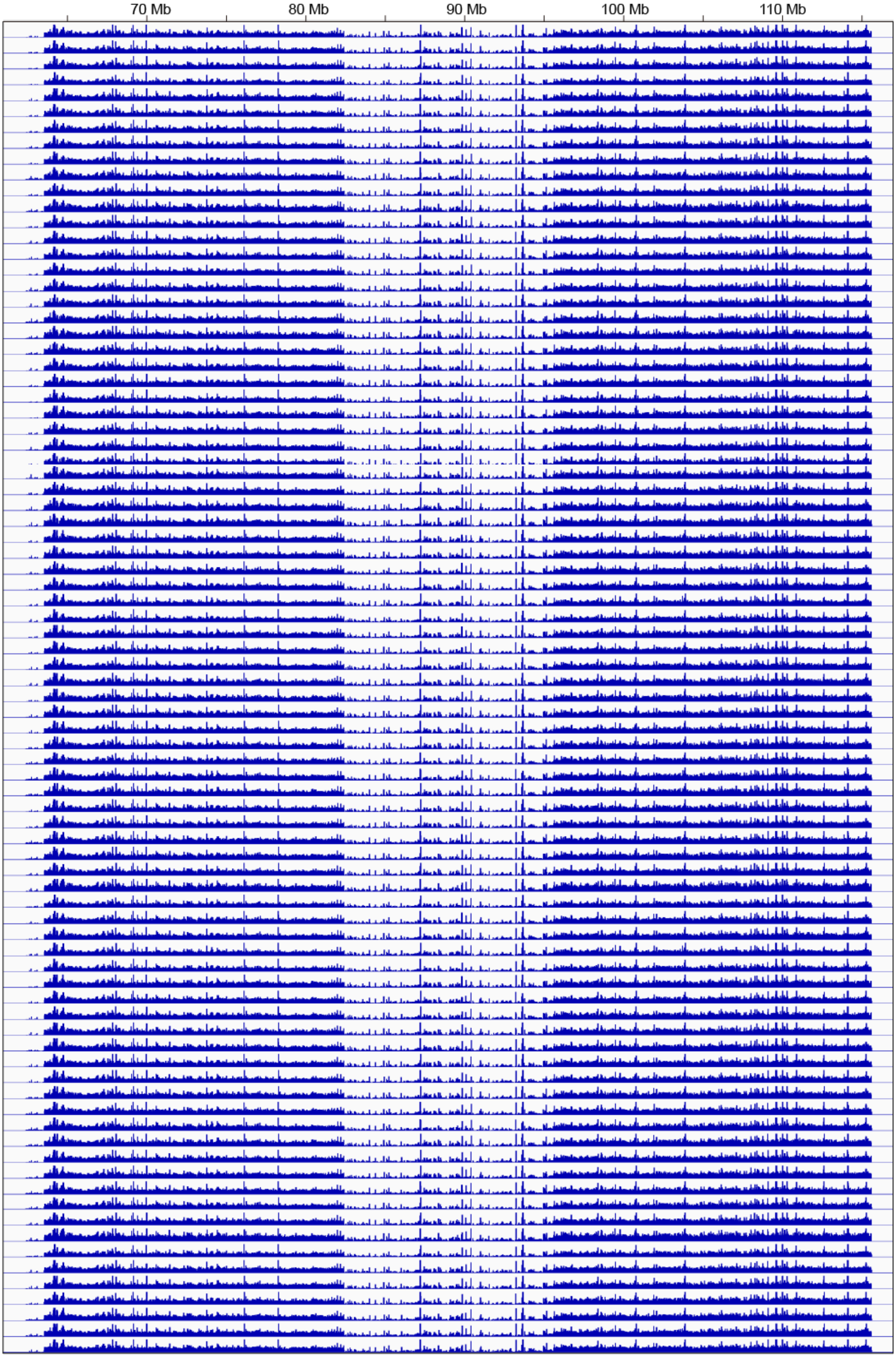

c

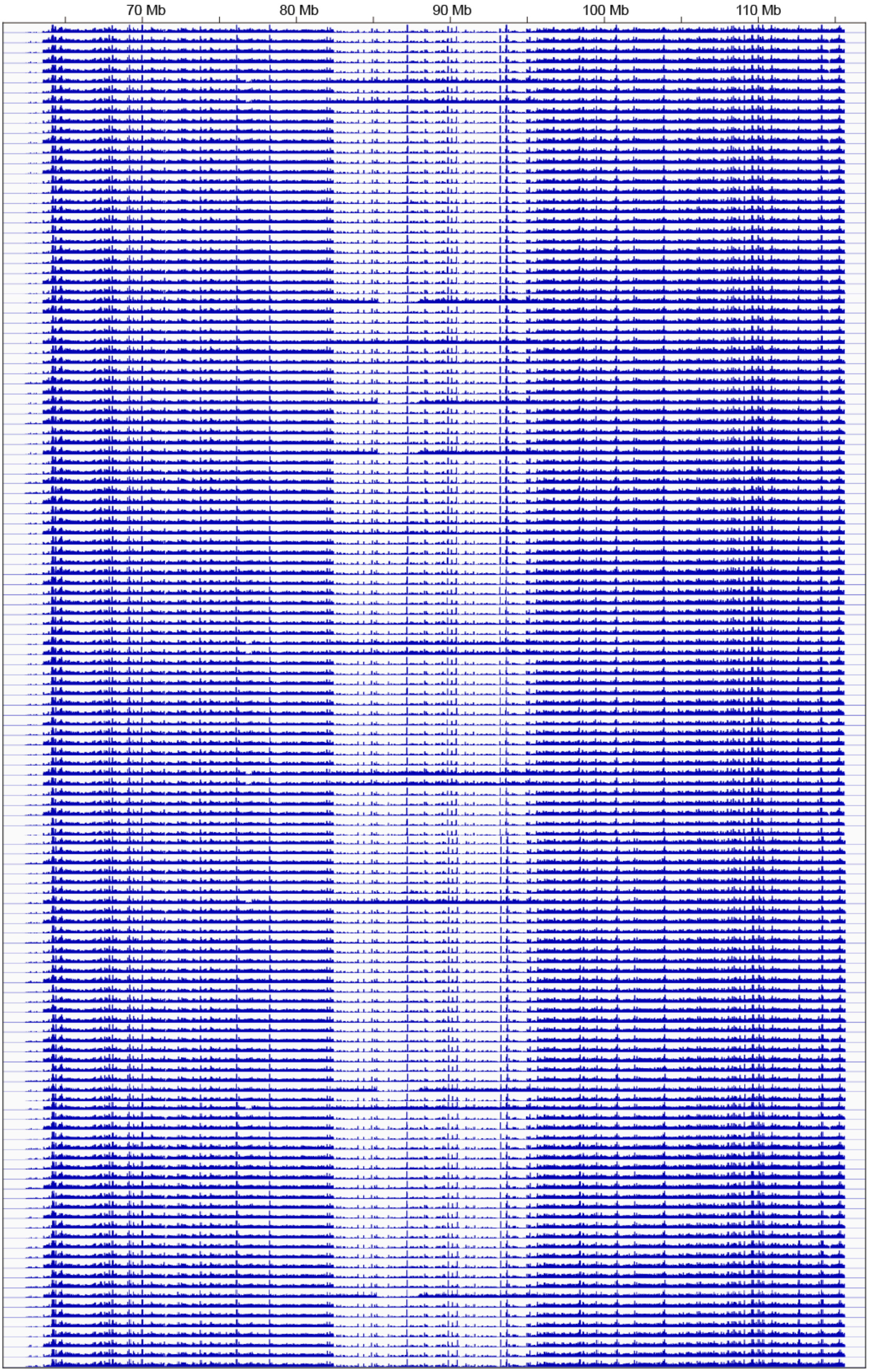

d

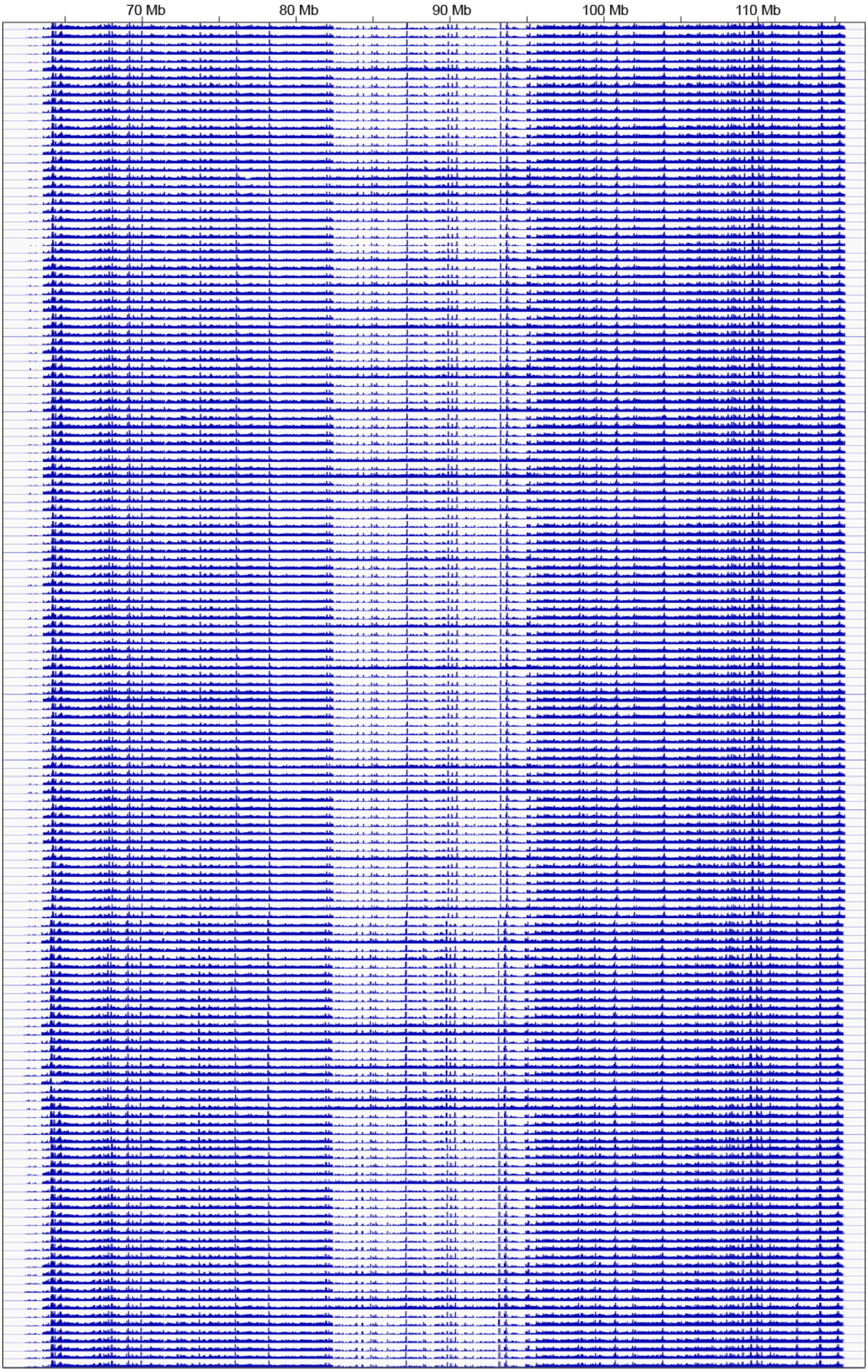

e

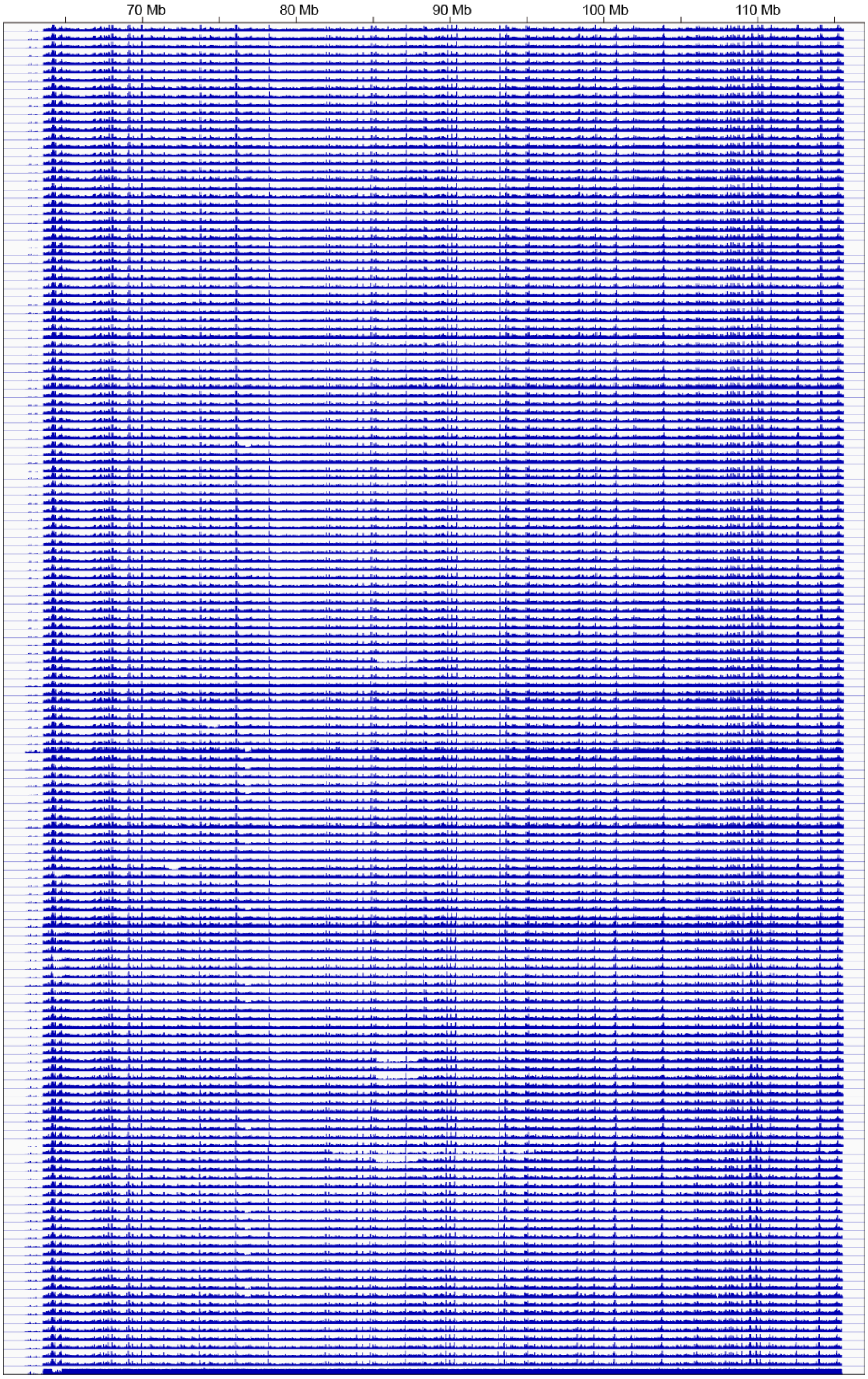

Supplementary Fig. 11 Structural variation (SV) among different botanical varieties. The coverage of 13.23 Mb SVs regions on Chr14, with K30076 as reference genome. **a,** wild diploid, **b,** *var. fastigiate*; **c,** *var. vulgaris*; **d,** *var. hypogaea*; **e,** *var. hypogaea* + *var. hirsuta*. Presence and absence are indicated by the coverage depth.

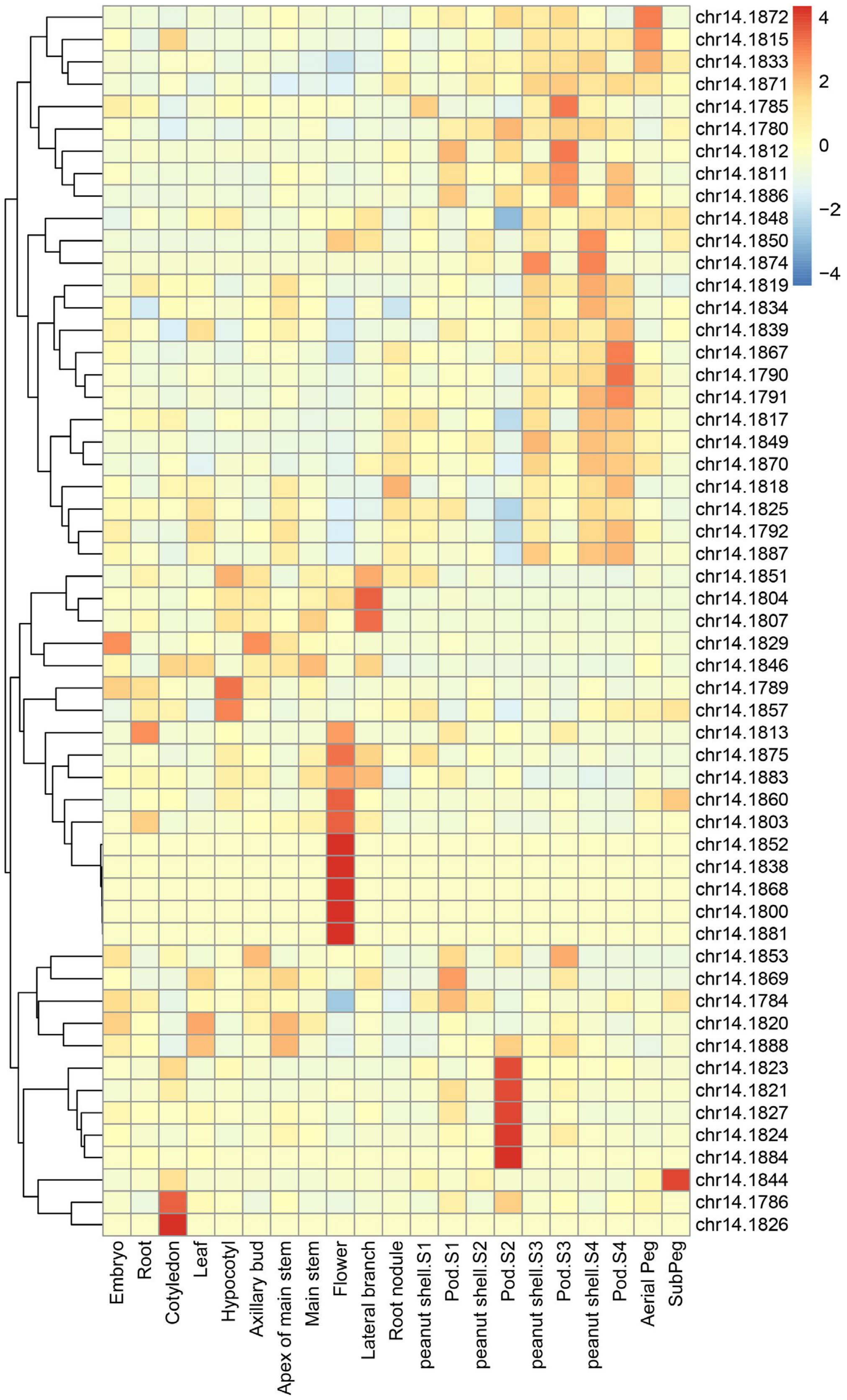

Supplementary Fig. 12 The expression pattern of structural variation (SV) genes present on Chromosome 14 in the S83 accession across different tissues.

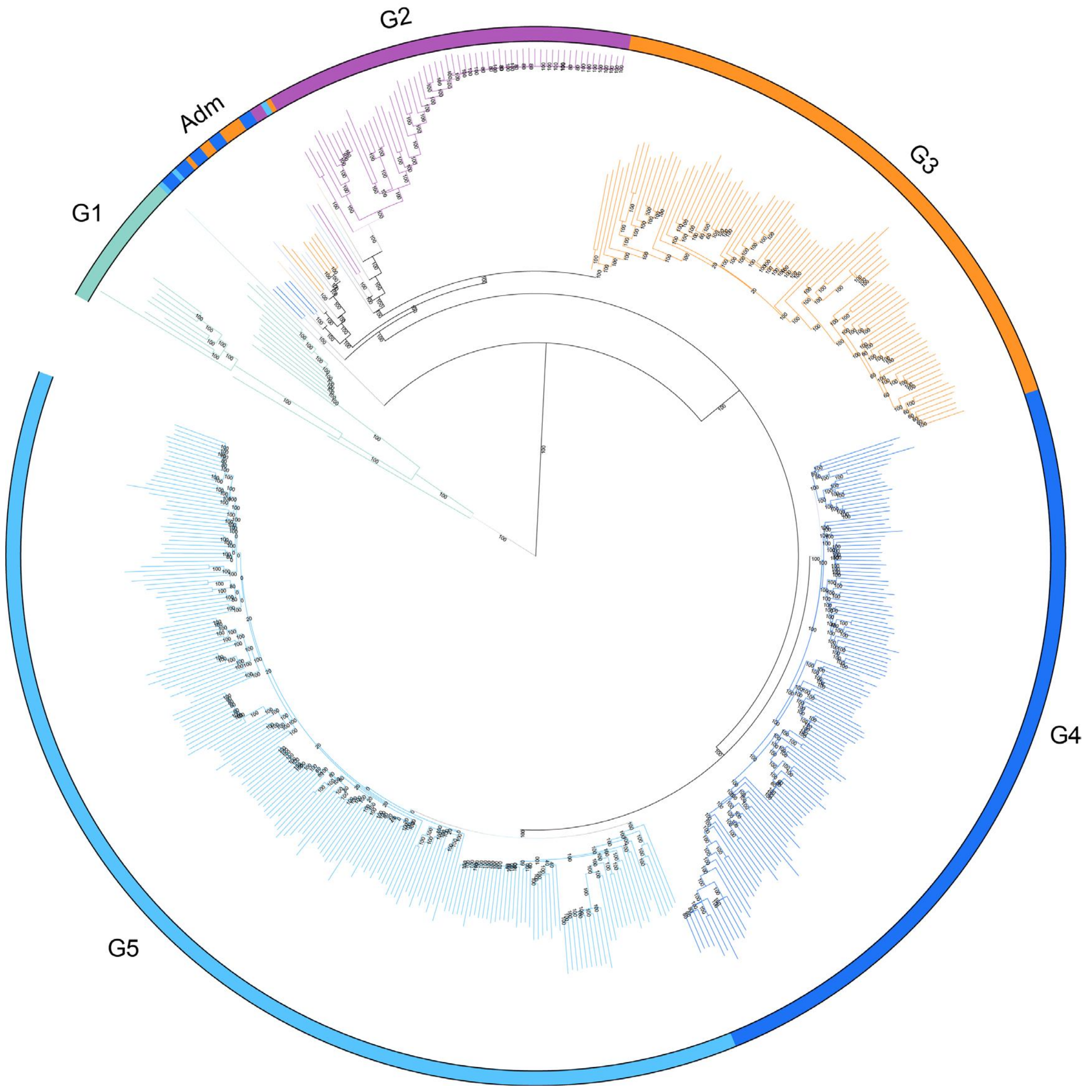

Supplementary Fig. 13 Phylogenetic tree of 521 peanut accessions. The neighbor-joining tree, with support values, divides the 521 peanut accessions into five main groups: wild diploid (G1), *var. fastigiate*(G2), *var. vulgaris* (G3), *var. hypogaea* (G4) and *var. hypogaea* + *var. hirsuta* (G5).

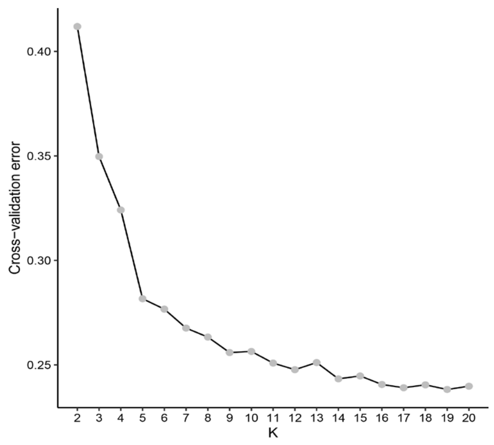

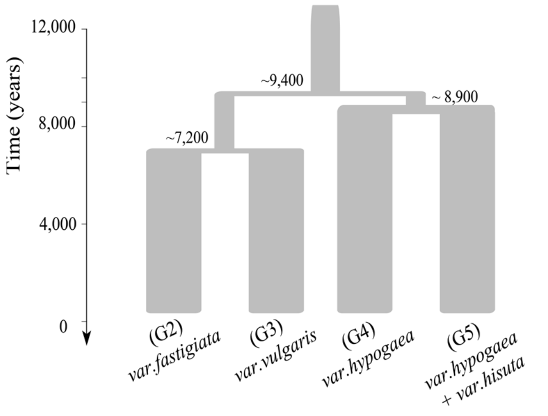

Supplementary Fig. 14 Population structure and demographic history of peanut populations. a, Cross-validation error values for different K values (K = 2 to 20). **b,** Schematic representation of divergence times estimated using fastsimcoal2. The vertical axis indicates the estimated timing of population divergence.

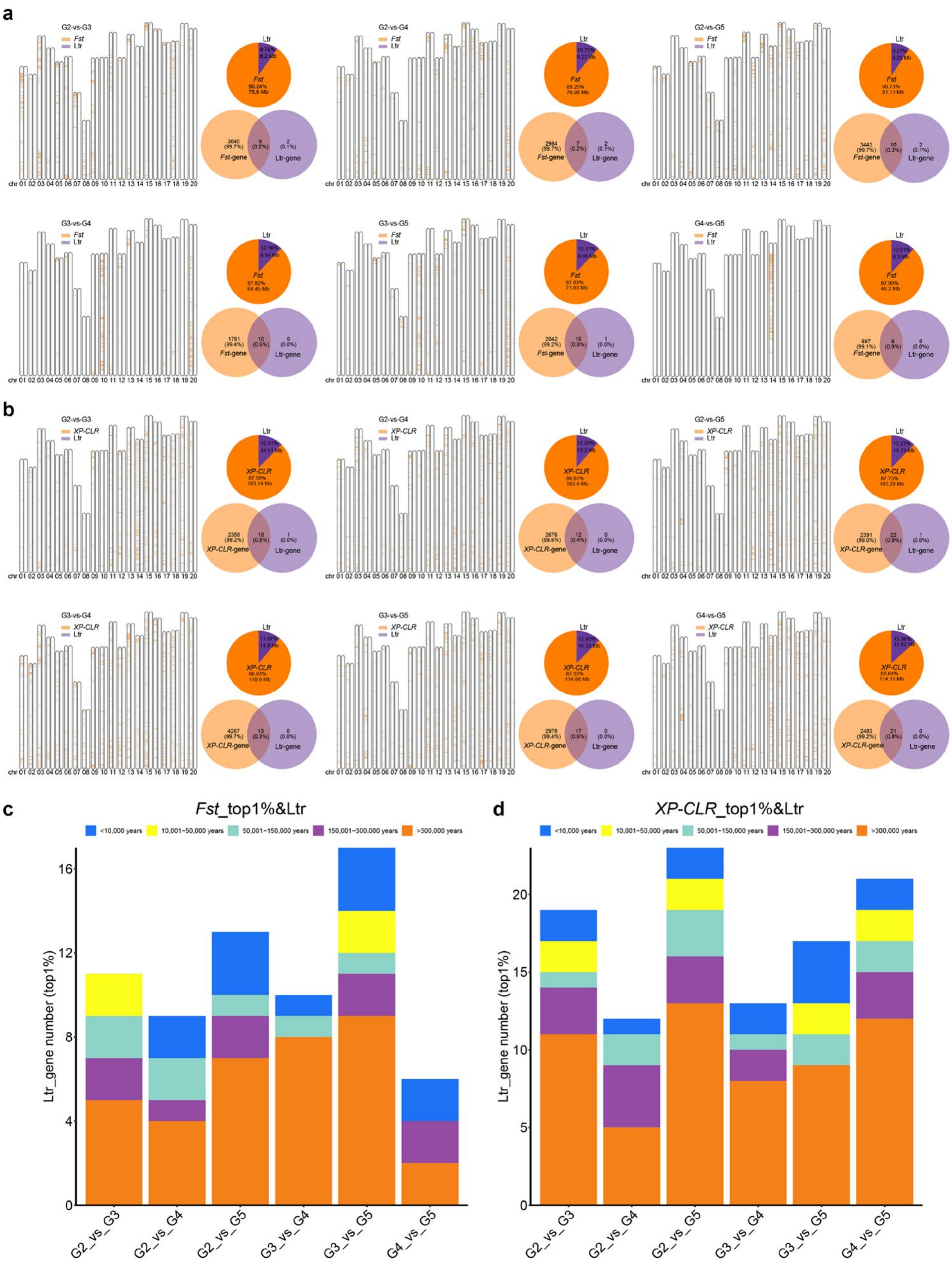

Supplementary Fig. 15 LTR insertion on selected sweep regions. The selective sweep analysis among six different populations was conducted using *Fst* and *XP-CLR*. Selective sweep regions (left) and selective sweep regions with LTR insertion regions (right) were plotted on the chromosome schematic in the left of Fig. **a** and **b**. The proportion of selective sweep intervals and LTRs, along with the corresponding number of genes, is presented as pie charts in the left of Fig A and B. LTR insertions are categorized into five time periods based on insertion times. *Fst*_top1% (**c**) and *XP-CLR*_top1% (**d**) refer to the top 1% intervals retained, respectively. LTR-genes are defined as genes that overlap with LTR elements.

a

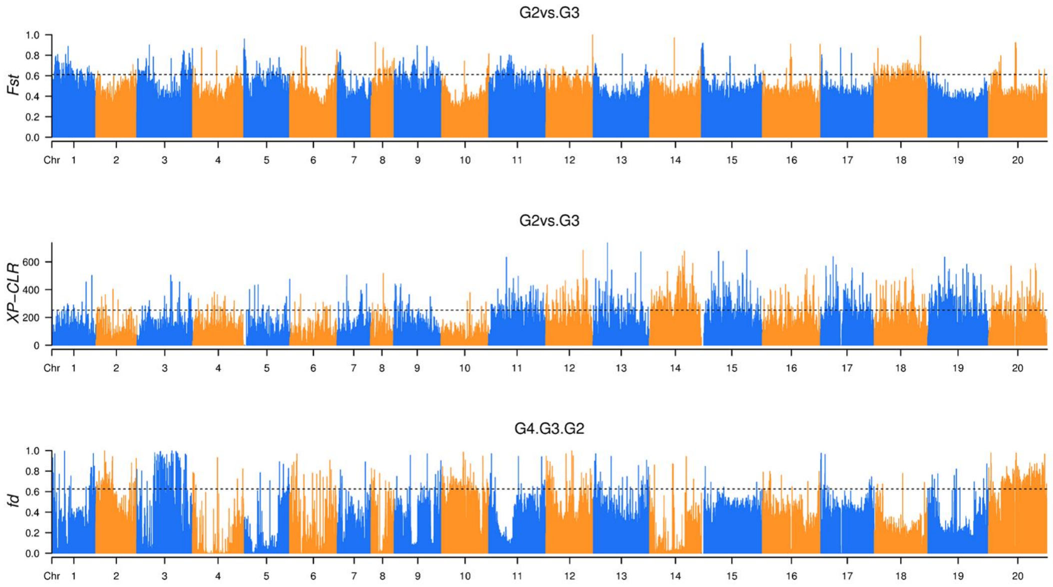

G2vs.G3
Fst
XP−CLR
G4.G3.G2
fd
Chr 1 2 3 4 5 6 7 8 9 10 11 12 13 14 15 16 17 18 19 20

b

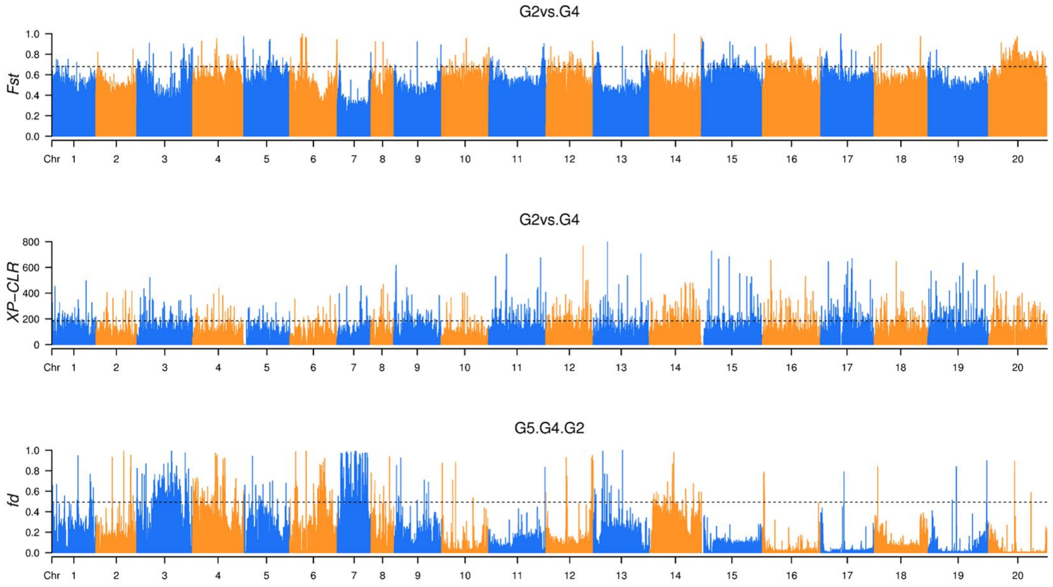

G2vs.G4
Fst
XP−CLR
G5.G4.G2
fd
Chr 1 2 3 4 5 6 7 8 9 10 11 12 13 14 15 16 17 18 19 20

c

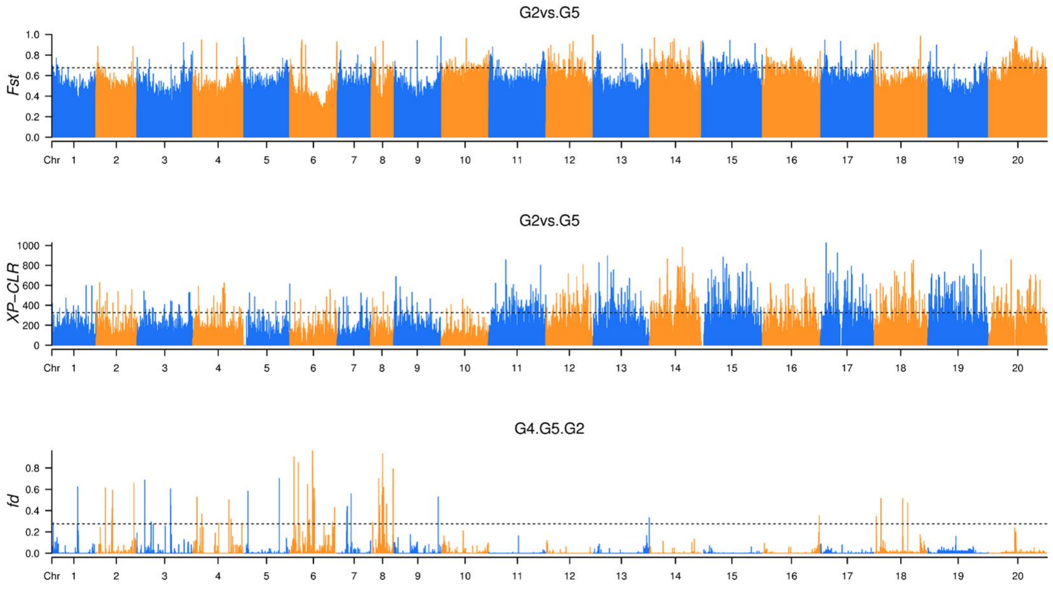
G2vs.G5
Fst
1.0
0.8
0.6
0.4
0.2
0.0
Chr 1 2 3 4 5 6 7 8 9 10 11 12 13 14 15 16 17 18 19 20
G2vs.G5
XP−CLR
1000
800
600
400
200
0
Chr 1 2 3 4 5 6 7 8 9 10 11 12 13 14 15 16 17 18 19 20
G4.G5.G2
fd
0.8
0.6
0.4
0.2
0.0
Chr 1 2 3 4 5 6 7 8 9 10 11 12 13 14 15 16 17 18 19 20

d

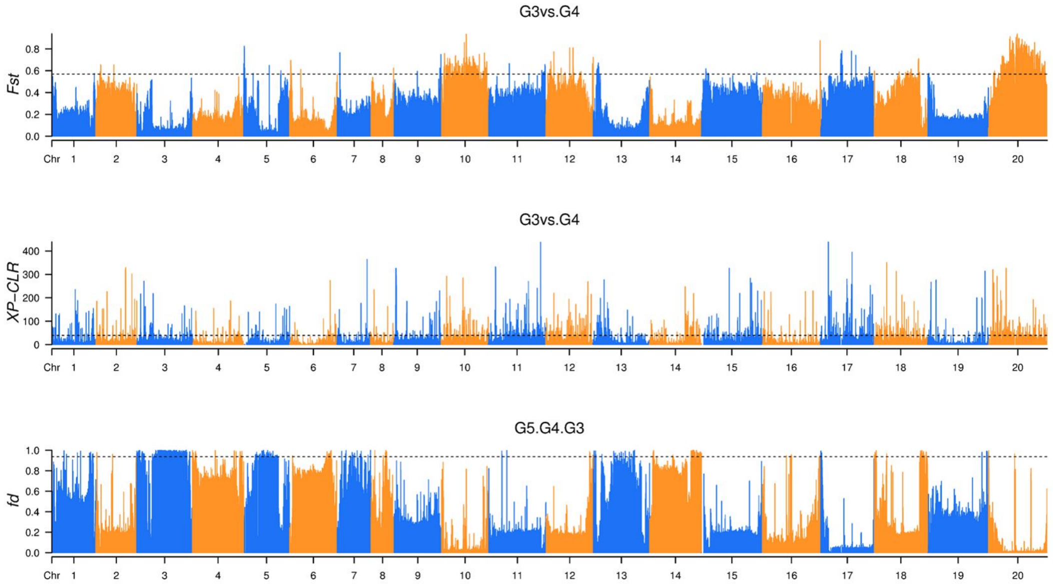
G3vs.G4
Fst
0.8
0.6
0.4
0.2
0.0
Chr 1 2 3 4 5 6 7 8 9 10 11 12 13 14 15 16 17 18 19 20
G3vs.G4
XP−CLR
400
300
200
100
0
Chr 1 2 3 4 5 6 7 8 9 10 11 12 13 14 15 16 17 18 19 20
G5.G4.G3
fd
1.0
0.8
0.6
0.4
0.2
0.0
Chr 1 2 3 4 5 6 7 8 9 10 11 12 13 14 15 16 17 18 19 20

e

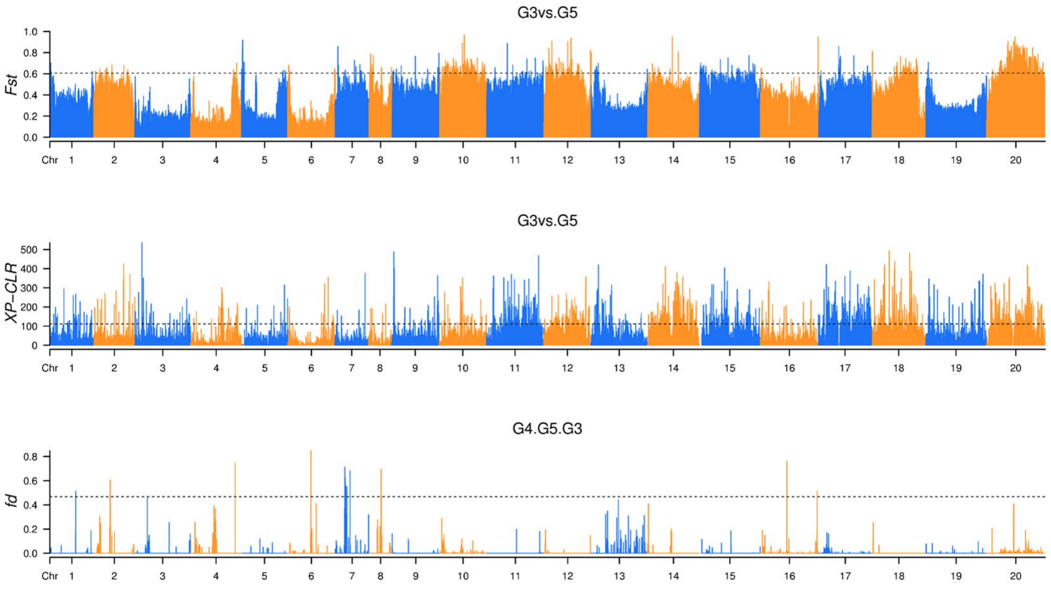

f

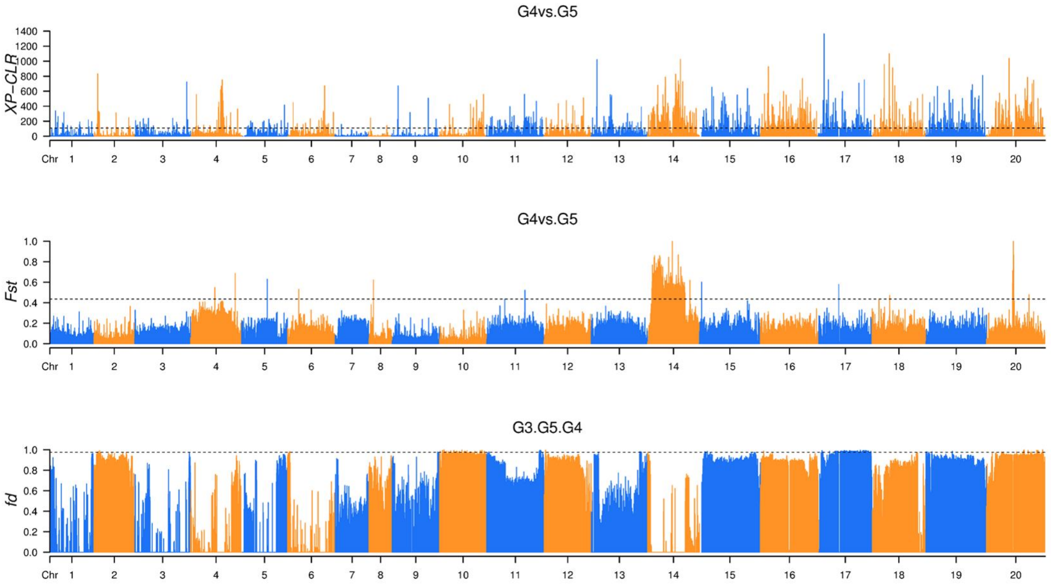

Supplementary Fig. 16 Selected sweep and introgression among different populations. *Fst*, *XP-CLT*, and *fd* analysis were conducted across different chromosomes among the following groups: **a,** G2-G3; **b,** G2-G4; **c,** G2-G5; **d,** G3-G4; **e,** G3-G5; **f,** G4-G5. The threshold line is set at the top 1%.

a

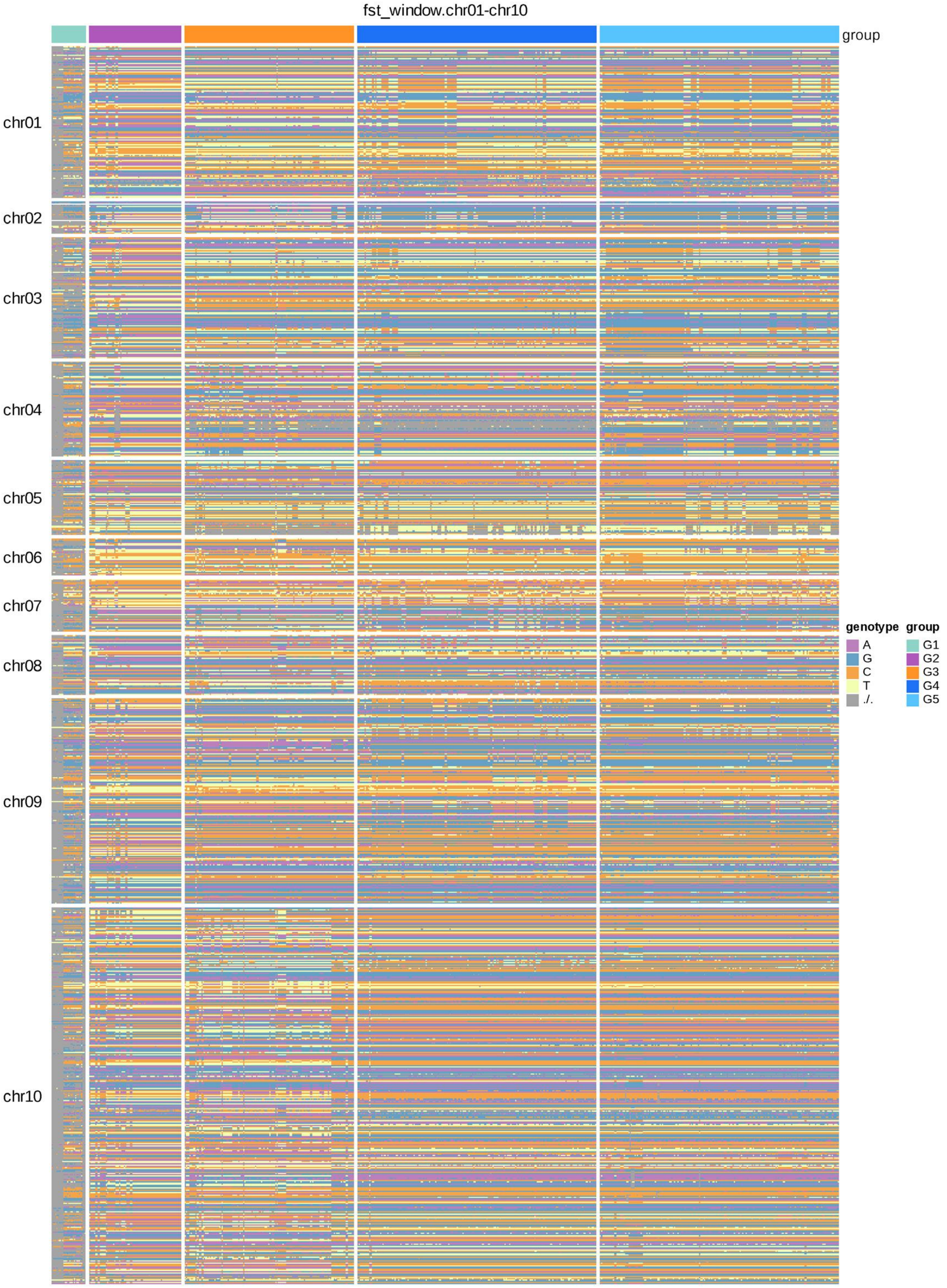

fst_window.chr01-chr10
group
chr01
chr02
chr03
chr04
chr05
chr06
chr07
chr08
chr09
chr10
genotype
A
G
C
T
./.
group
G1
G2
G3
G4
G5

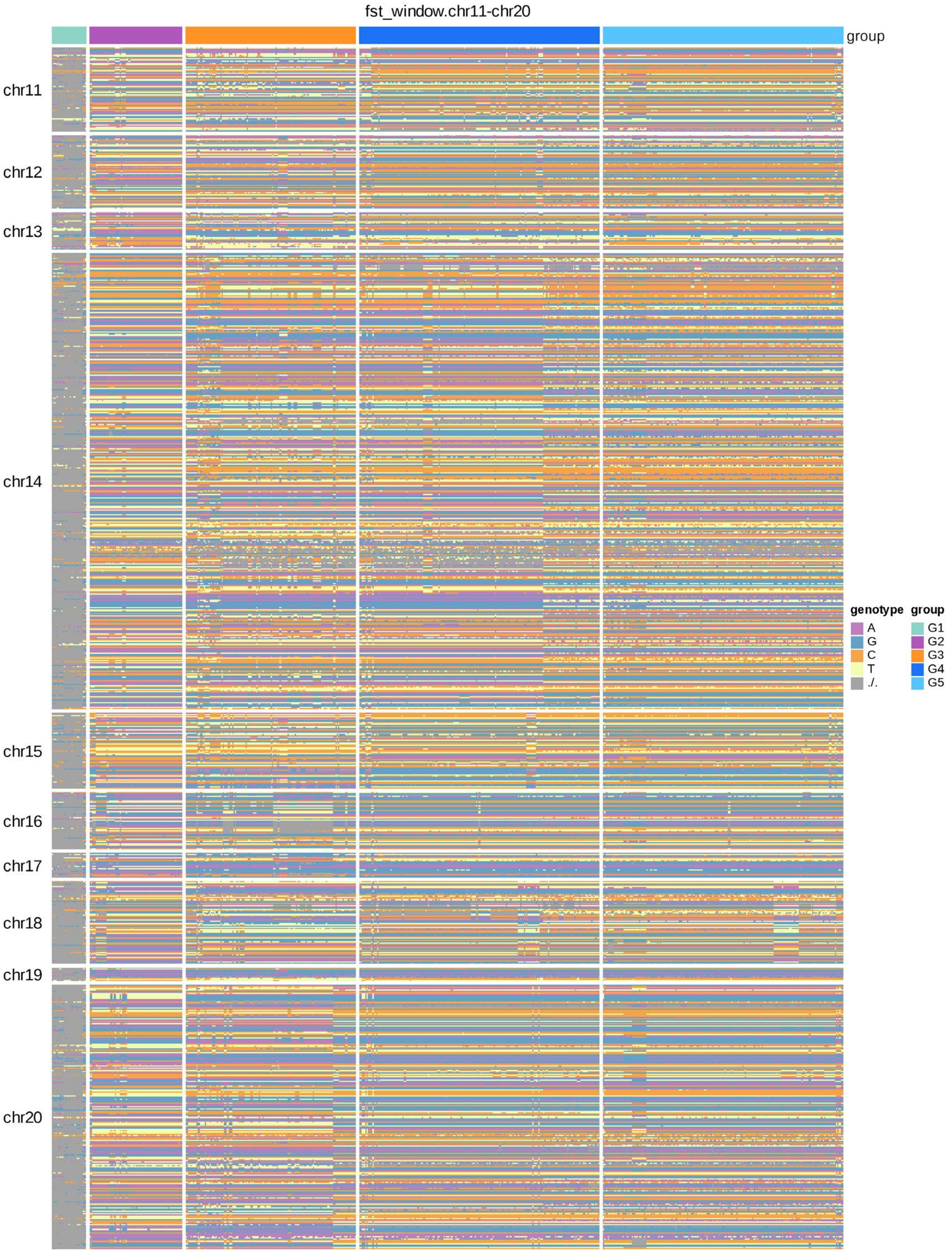

fst_window.chr11-chr20
group
chr11
chr12
chr13
chr14
chr15
chr16
chr17
chr18
chr19
chr20
genotype
A
G
C
T
./.
group
G1
G2
G3
G4
G5

b

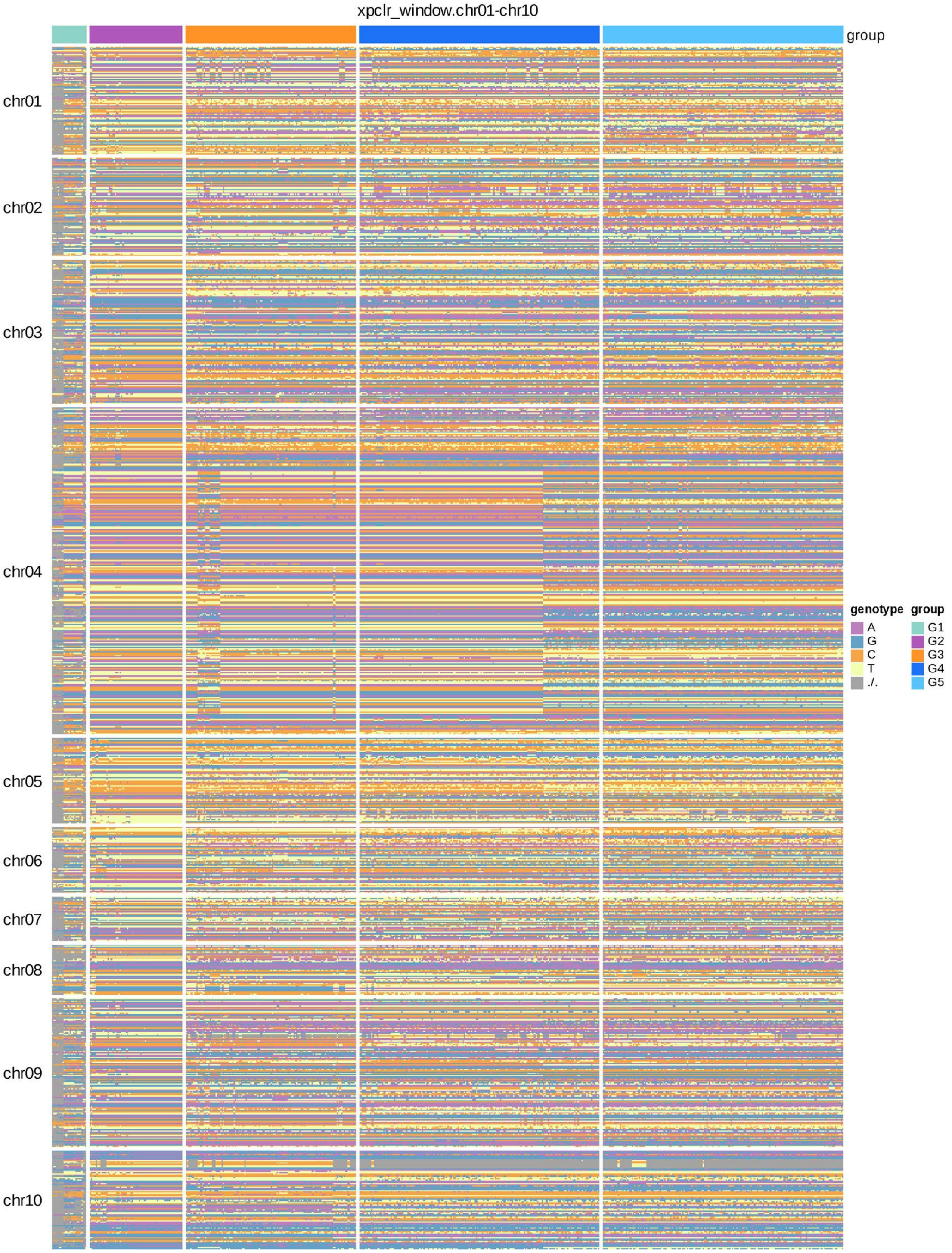

xpclr_window.chr01-chr10
group
chr01
chr02
chr03
chr04
chr05
chr06
chr07
chr08
chr09
chr10
genotype
A
G
C
T
./.
group
G1
G2
G3
G4
G5

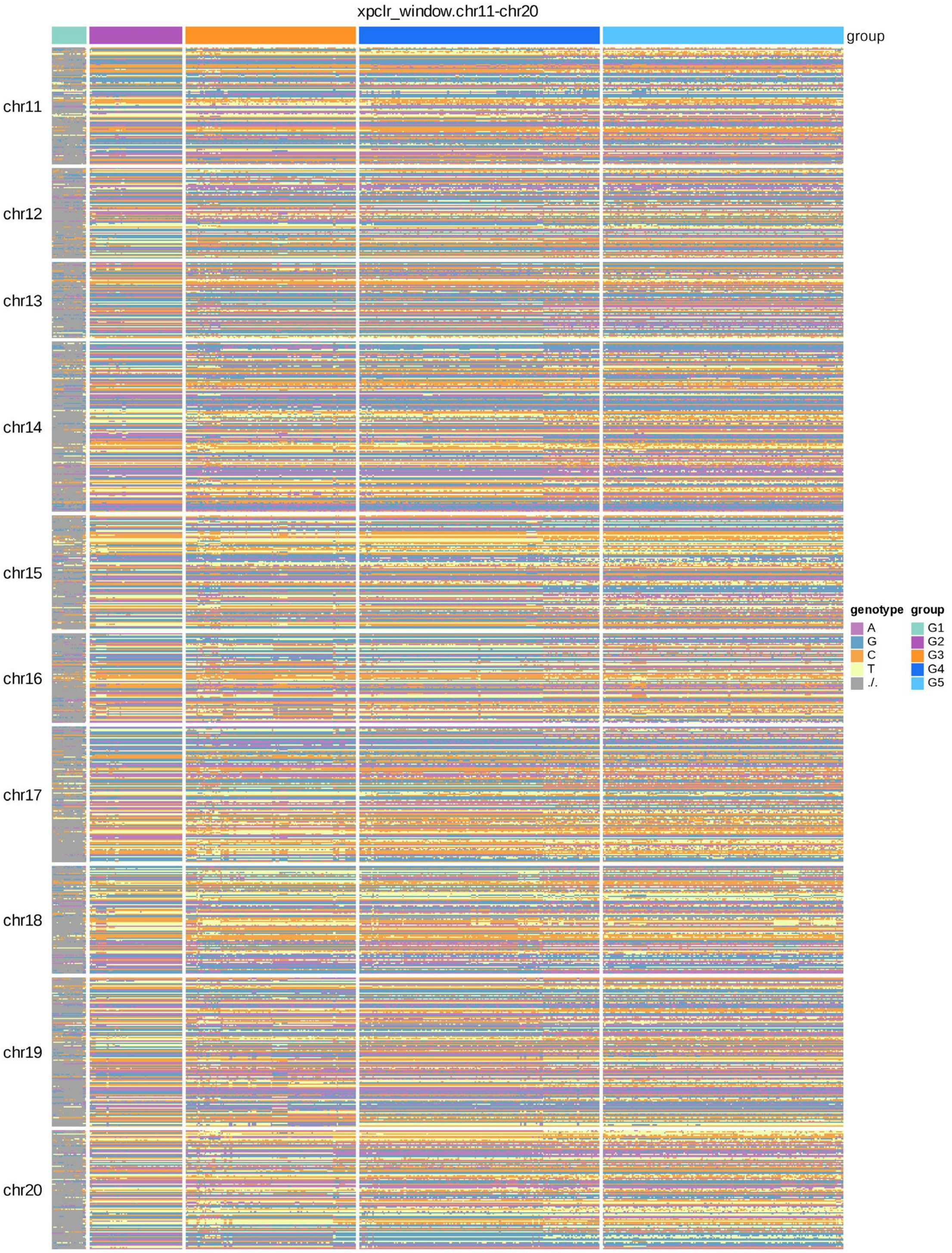

xpclr_window.chr11-chr20
group
chr11
chr12
chr13
chr14
chr15
chr16
chr17
chr18
chr19
chr20
genotype
A
G
C
T
./.
group
G1
G2
G3
G4
G5

c

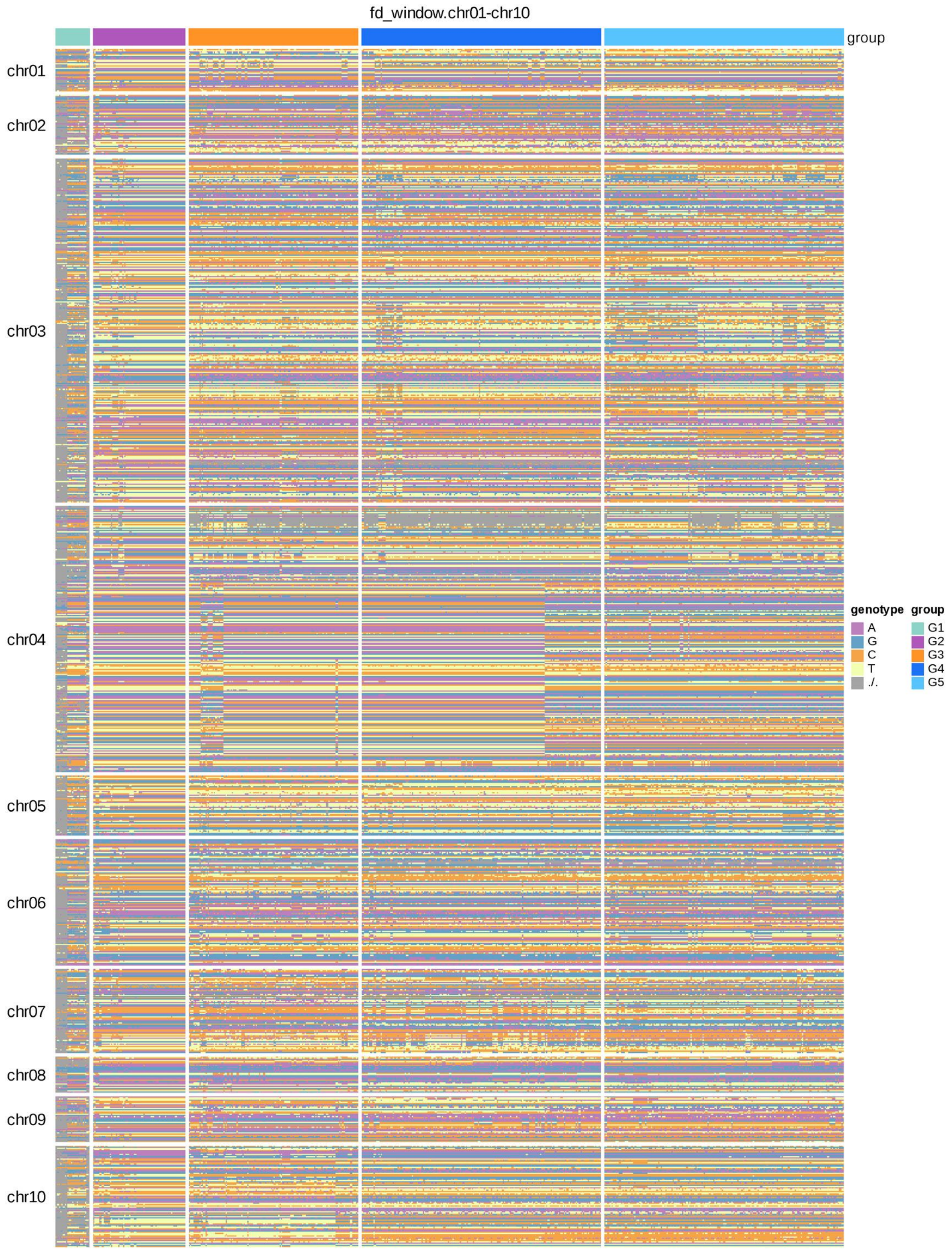

fd_window.chr01-chr10
group
chr01
chr02
chr03
chr04
chr05
chr06
chr07
chr08
chr09
chr10
genotype
A
G
C
T
./.
group
G1
G2
G3
G4
G5

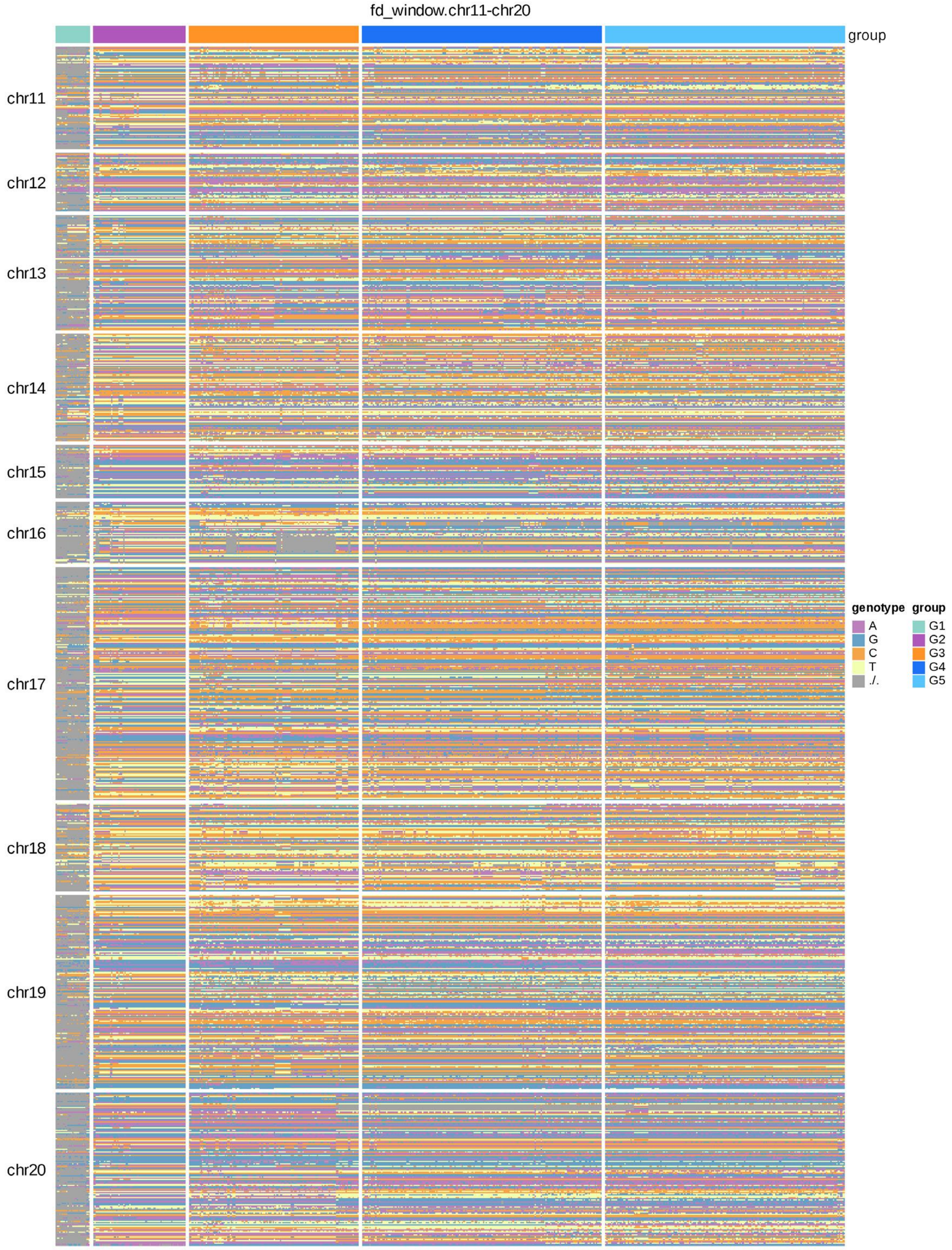

Supplementary Fig. 17 SNP Variation Types in Selected and Introgressed Regions. The distribution of SNP variants (A, G, C, T, and ./.) across Group1 to Group5 is visualized with different colors. The S245 reference genome is used for comparison. **a,** Top 1% *Fst* regions. **b,** Top 1% *XP-CLR* regions. **c,** Top 1% Introgressed regions.

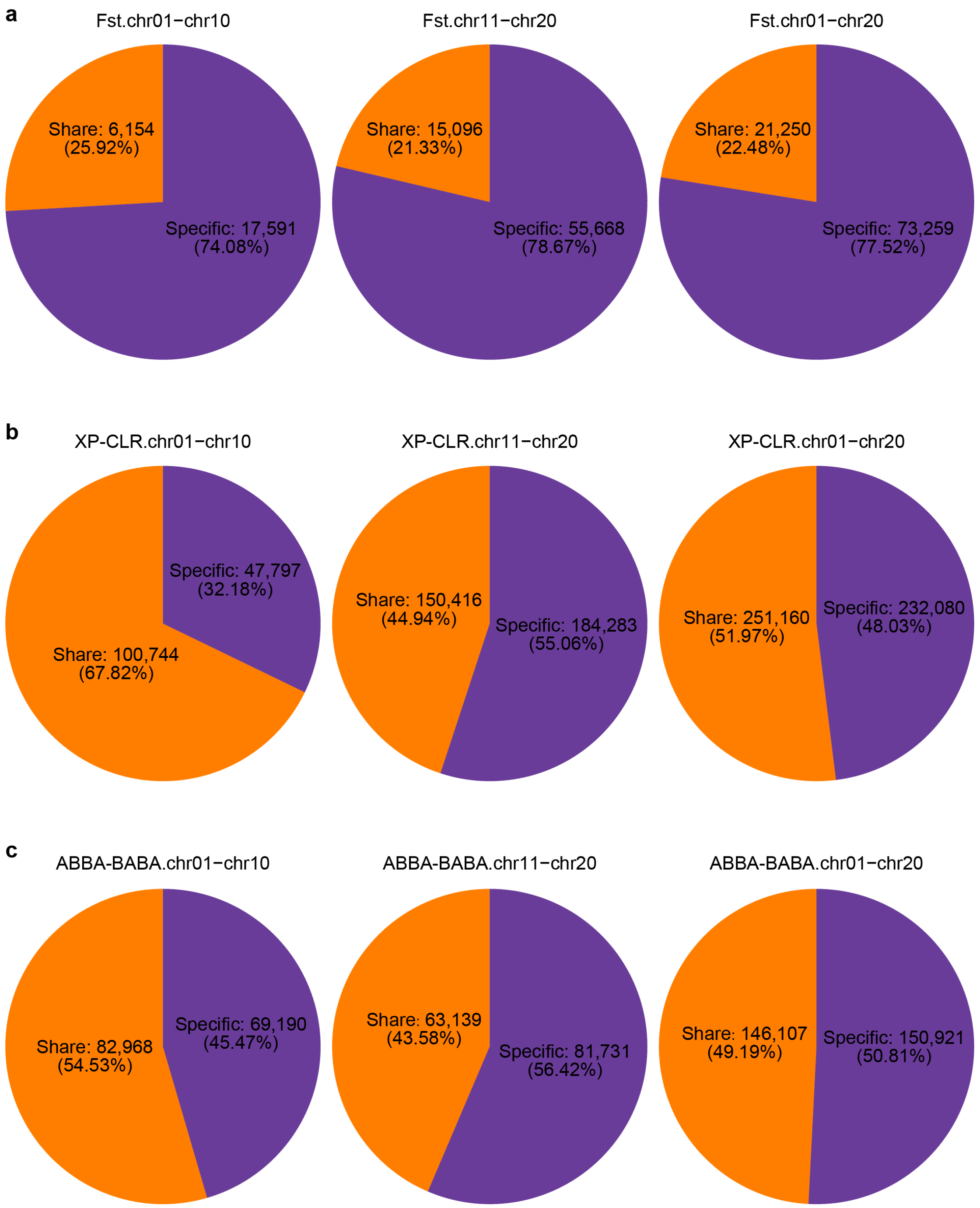

Supplementary Fig. 18 Percentage of share and specific variation between diploids and tetraploids. The variation type in tetraploids is defined as shared variation if it exists in the wild accessions; otherwise, it is considered tetraploid-specific variation. **a,** Top 1% *Fst* regions. **b,** Top 1% *XP-CLR* regions. **c,** Top 1% introgressed regions. From left to right, represent the At (chr01-chr10), Bt (chr11-chr20), and At Bt (chr01-chr20) subgenomes.

a

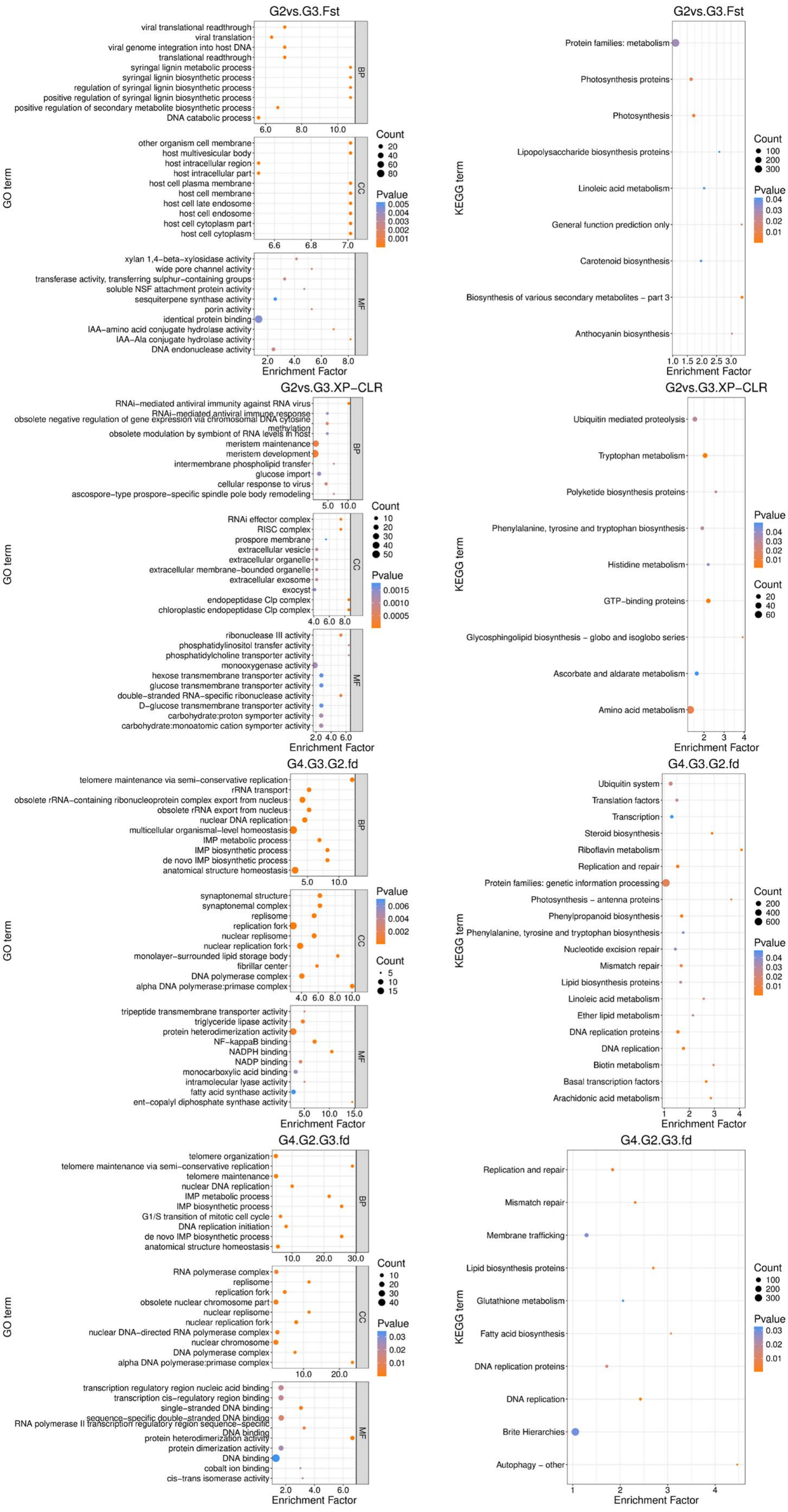
G2vs.G3.Fst
viral translational readthrough
viral translation
viral genome integration into host DNA
translational readthrough
syringal lignin metabolic process
syringal lignin biosynthetic process
regulation of syringal lignin biosynthetic process
positive regulation of syringal lignin biosynthetic process
positive regulation of secondary metabolite biosynthetic process
DNA catabolic process
other organism cell membrane
host multivesicular body
host intracellular region
host intracellular part
host cell plasma membrane
host cell membrane
host cell late endosome
host cell endosome
host cell cytoplasm part
host cell cytoplasm
xylan 1,4−beta−xylosidase activity
wide pore channel activity
transferase activity, transferring sulphur−containing groups
soluble NSF attachment protein activity
sesquiterpene synthase activity
porin activity
identical protein binding
IAA−amino acid conjugate hydrolase activity
IAA−Ala conjugate hydrolase activity
DNA endonuclease activity
BP
CC
MF
GO term
Count
Pvalue
Enrichment Factor
Protein families: metabolism
Photosynthesis proteins
Photosynthesis
Lipopolysaccharide biosynthesis proteins
Linoleic acid metabolism
General function prediction only
Carotenoid biosynthesis
Biosynthesis of various secondary metabolites − part 3
Anthocyanin biosynthesis
KEGG term
G2vs.G3.XP−CLR
RNAi−mediated antiviral immunity against RNA virus
RNAi−mediated antiviral immune response
obsolete negative regulation of gene expression via chromosomal DNA cytosine methylation
obsolete modulation by symbiont of RNA levels in host
meristem maintenance
meristem development
intermembrane phospholipid transfer
glucose import
cellular response to virus
ascospore−type prospore−specific spindle pole body remodeling
RNAi effector complex
RISC complex
prospore membrane
extracellular vesicle
extracellular organelle
extracellular membrane−bounded organelle
extracellular exosome
exocyst
endopeptidase Clp complex
chloroplastic endopeptidase Clp complex
ribonuclease III activity
phosphatidylinositol transfer activity
phosphatidylcholine transporter activity
monooxygenase activity
hexose transmembrane transporter activity
glucose transmembrane transporter activity
double−stranded RNA−specific ribonuclease activity
D−glucose transmembrane transporter activity
carbohydrate:proton symporter activity
carbohydrate:monoatomic cation symporter activity
Ubiquitin mediated proteolysis
Tryptophan metabolism
Polyketide biosynthesis proteins
Phenylalanine, tyrosine and tryptophan biosynthesis
Histidine metabolism
GTP−binding proteins
Glycosphingolipid biosynthesis − globo and isoglobo series
Ascorbate and aldarate metabolism
Amino acid metabolism
G4.G3.G2.fd
telomere maintenance via semi−conservative replication
rRNA transport
obsolete rRNA−containing ribonucleoprotein complex export from nucleus
obsolete rRNA export from nucleus
nuclear DNA replication
multicellular organismal−level homeostasis
IMP metabolic process
IMP biosynthetic process
de novo IMP biosynthetic process
anatomical structure homeostasis
synaptonemal structure
synaptonemal complex
replisome
replication fork
nuclear replisome
nuclear replication fork
monolayer−surrounded lipid storage body
fibrillar center
DNA polymerase complex
alpha DNA polymerase:primase complex
tripeptide transmembrane transporter activity
triglyceride lipase activity
protein heterodimerization activity
NF−kappaB binding
NADPH binding
NADP binding
monocarboxylic acid binding
intramolecular lyase activity
fatty acid synthase activity
ent−copalyl diphosphate synthase activity
Ubiquitin system
Translation factors
Transcription
Steroid biosynthesis
Riboflavin metabolism
Replication and repair
Protein families: genetic information processing
Photosynthesis − antenna proteins
Phenylpropanoid biosynthesis
Phenylalanine, tyrosine and tryptophan biosynthesis
Nucleotide excision repair
Mismatch repair
Lipid biosynthesis proteins
Linoleic acid metabolism
Ether lipid metabolism
DNA replication proteins
DNA replication
Biotin metabolism
Basal transcription factors
Arachidonic acid metabolism
G4.G2.G3.fd
telomere organization
telomere maintenance via semi−conservative replication
telomere maintenance
nuclear DNA replication
IMP metabolic process
IMP biosynthetic process
G1/S transition of mitotic cell cycle
DNA replication initiation
de novo IMP biosynthetic process
anatomical structure homeostasis
RNA polymerase complex
replisome
replication fork
obsolete nuclear chromosome part
nuclear replisome
nuclear replication fork
nuclear DNA−directed RNA polymerase complex
nuclear chromosome
DNA polymerase complex
alpha DNA polymerase:primase complex
transcription regulatory region nucleic acid binding
transcription cis−regulatory region binding
single−stranded DNA binding
sequence−specific double−stranded DNA binding
RNA polymerase II transcription regulatory region sequence−specific DNA binding
protein heterodimerization activity
protein dimerization activity
DNA binding
cobalt ion binding
cis−trans isomerase activity
Replication and repair
Mismatch repair
Membrane trafficking
Lipid biosynthesis proteins
Glutathione metabolism
Fatty acid biosynthesis
DNA replication proteins
DNA replication
Brite Hierarchies
Autophagy − other

b

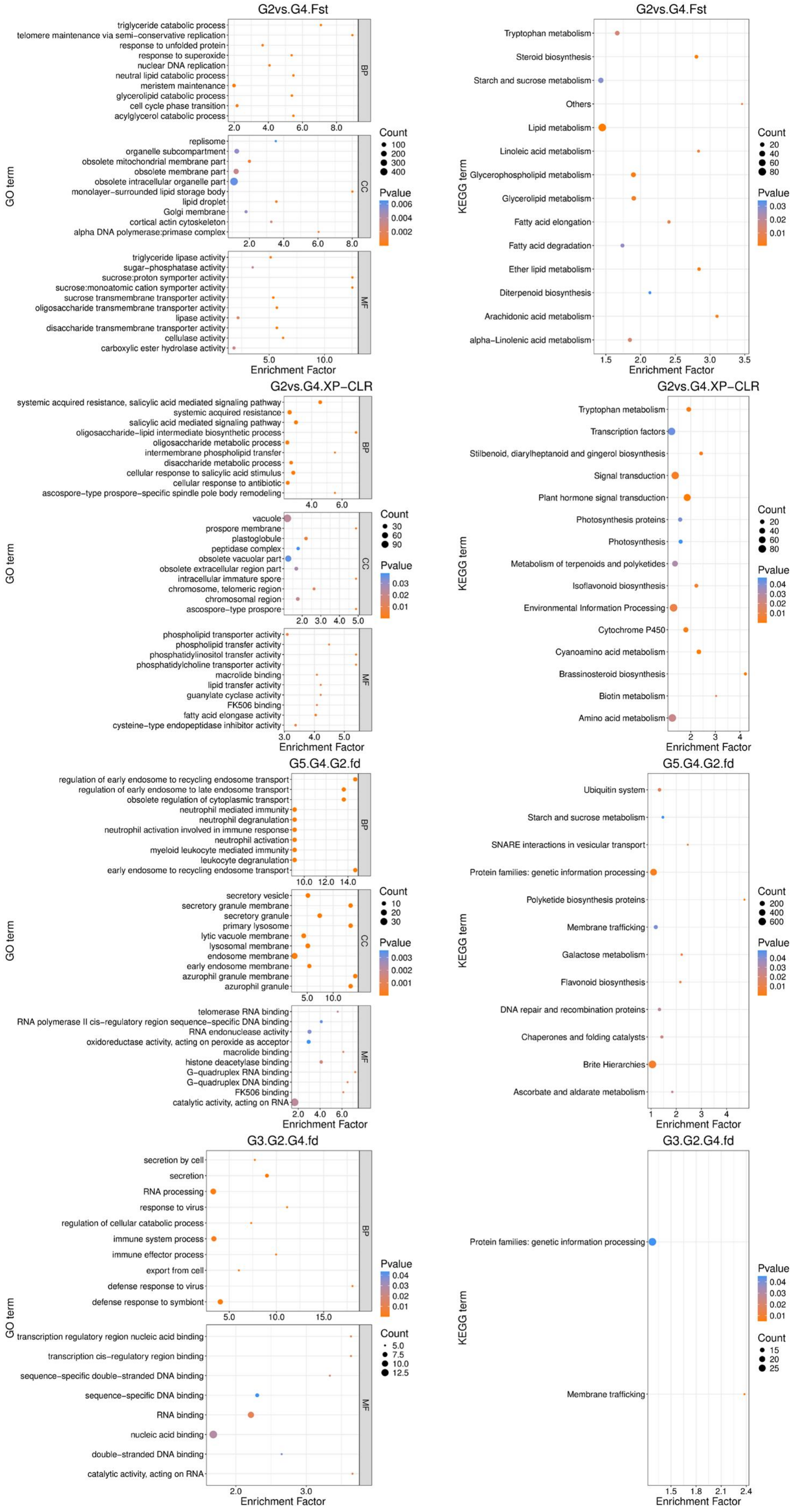
G2vs.G4.Fst
triglyceride catabolic process
telomere maintenance via semi−conservative replication
response to unfolded protein
response to superoxide
nuclear DNA replication
neutral lipid catabolic process
meristem maintenance
glycerolipid catabolic process
cell cycle phase transition
acylglycerol catabolic process
replisome
organelle subcompartment
obsolete mitochondrial membrane part
obsolete membrane part
obsolete intracellular organelle part
monolayer−surrounded lipid storage body
lipid droplet
Golgi membrane
cortical actin cytoskeleton
alpha DNA polymerase:primase complex
triglyceride lipase activity
sugar−phosphatase activity
sucrose:proton symporter activity
sucrose:monoatomic cation symporter activity
sucrose transmembrane transporter activity
oligosaccharide transmembrane transporter activity
lipase activity
disaccharide transmembrane transporter activity
cellulase activity
carboxylic ester hydrolase activity
BP
CC
MF
GO term
Enrichment Factor
Count
Pvalue
Tryptophan metabolism
Steroid biosynthesis
Starch and sucrose metabolism
Others
Lipid metabolism
Linoleic acid metabolism
Glycerophospholipid metabolism
Glycerolipid metabolism
Fatty acid elongation
Fatty acid degradation
Ether lipid metabolism
Diterpenoid biosynthesis
Arachidonic acid metabolism
alpha−Linolenic acid metabolism
KEGG term
G2vs.G4.XP−CLR
systemic acquired resistance, salicylic acid mediated signaling pathway
systemic acquired resistance
salicylic acid mediated signaling pathway
oligosaccharide−lipid intermediate biosynthetic process
oligosaccharide metabolic process
intermembrane phospholipid transfer
disaccharide metabolic process
cellular response to salicylic acid stimulus
cellular response to antibiotic
ascospore−type prospore−specific spindle pole body remodeling
vacuole
prospore membrane
plastoglobule
peptidase complex
obsolete vacuolar part
obsolete extracellular region part
intracellular immature spore
chromosome, telomeric region
chromosomal region
ascospore−type prospore
phospholipid transporter activity
phospholipid transfer activity
phosphatidylinositol transfer activity
phosphatidylcholine transporter activity
macrolide binding
lipid transfer activity
guanylate cyclase activity
FK506 binding
fatty acid elongase activity
cysteine−type endopeptidase inhibitor activity
Transcription factors
Stilbenoid, diarylheptanoid and gingerol biosynthesis
Signal transduction
Plant hormone signal transduction
Photosynthesis proteins
Photosynthesis
Metabolism of terpenoids and polyketides
Isoflavonoid biosynthesis
Environmental Information Processing
Cytochrome P450
Cyanoamino acid metabolism
Brassinosteroid biosynthesis
Biotin metabolism
Amino acid metabolism
G5.G4.G2.fd
regulation of early endosome to recycling endosome transport
regulation of early endosome to late endosome transport
obsolete regulation of cytoplasmic transport
neutrophil mediated immunity
neutrophil degranulation
neutrophil activation involved in immune response
neutrophil activation
myeloid leukocyte mediated immunity
leukocyte degranulation
early endosome to recycling endosome transport
secretory vesicle
secretory granule membrane
secretory granule
primary lysosome
lytic vacuole membrane
lysosomal membrane
endosome membrane
early endosome membrane
azurophil granule membrane
azurophil granule
telomerase RNA binding
RNA polymerase II cis−regulatory region sequence−specific DNA binding
RNA endonuclease activity
oxidoreductase activity, acting on peroxide as acceptor
macrolide binding
histone deacetylase binding
G−quadruplex RNA binding
G−quadruplex DNA binding
FK506 binding
catalytic activity, acting on RNA
Ubiquitin system
SNARE interactions in vesicular transport
Protein families: genetic information processing
Polyketide biosynthesis proteins
Membrane trafficking
Galactose metabolism
Flavonoid biosynthesis
DNA repair and recombination proteins
Chaperones and folding catalysts
Brite Hierarchies
Ascorbate and aldarate metabolism
G3.G2.G4.fd
secretion by cell
secretion
RNA processing
response to virus
regulation of cellular catabolic process
immune system process
immune effector process
export from cell
defense response to virus
defense response to symbiont
transcription regulatory region nucleic acid binding
transcription cis−regulatory region binding
sequence−specific double−stranded DNA binding
sequence−specific DNA binding
RNA binding
nucleic acid binding
double−stranded DNA binding
catalytic activity, acting on RNA

c

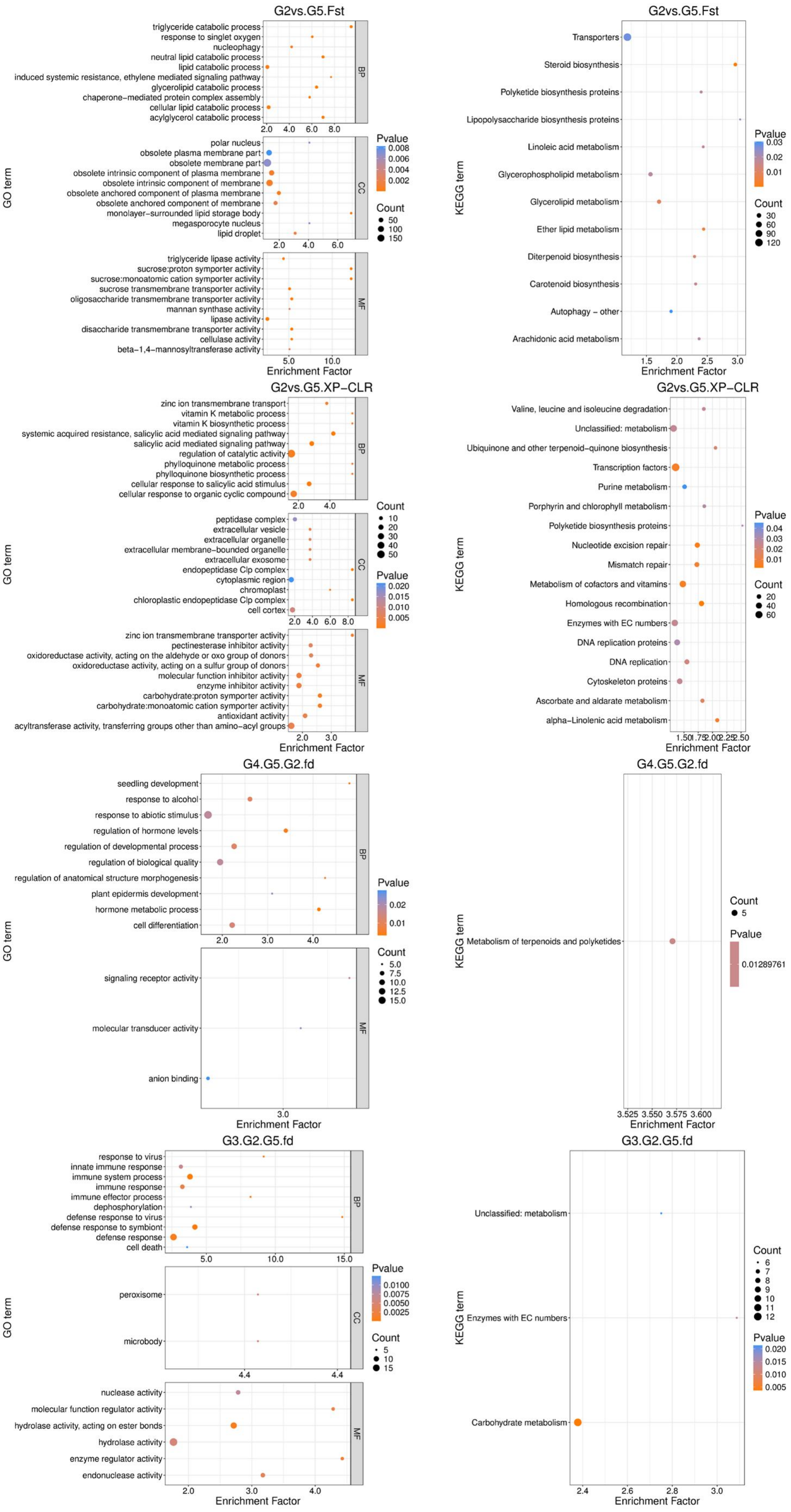
G2vs.G5.Fst
triglyceride catabolic process
response to singlet oxygen
nucleophagy
neutral lipid catabolic process
lipid catabolic process
induced systemic resistance, ethylene mediated signaling pathway
glycerolipid catabolic process
chaperone−mediated protein complex assembly
cellular lipid catabolic process
acylglycerol catabolic process
polar nucleus
obsolete plasma membrane part
obsolete membrane part
obsolete intrinsic component of plasma membrane
obsolete intrinsic component of membrane
obsolete anchored component of plasma membrane
obsolete anchored component of membrane
monolayer−surrounded lipid storage body
megasporocyte nucleus
lipid droplet
triglyceride lipase activity
sucrose:proton symporter activity
sucrose:monoatomic cation symporter activity
sucrose transmembrane transporter activity
oligosaccharide transmembrane transporter activity
mannan synthase activity
lipase activity
disaccharide transmembrane transporter activity
cellulase activity
beta−1,4−mannosyltransferase activity
BP
CC
MF
GO term
Enrichment Factor
Pvalue
Count
Transporters
Steroid biosynthesis
Polyketide biosynthesis proteins
Lipopolysaccharide biosynthesis proteins
Linoleic acid metabolism
Glycerophospholipid metabolism
Glycerolipid metabolism
Ether lipid metabolism
Diterpenoid biosynthesis
Carotenoid biosynthesis
Autophagy − other
Arachidonic acid metabolism
KEGG term
G2vs.G5.XP−CLR
zinc ion transmembrane transport
vitamin K metabolic process
vitamin K biosynthetic process
systemic acquired resistance, salicylic acid mediated signaling pathway
salicylic acid mediated signaling pathway
regulation of catalytic activity
phylloquinone metabolic process
phylloquinone biosynthetic process
cellular response to salicylic acid stimulus
cellular response to organic cyclic compound
peptidase complex
extracellular vesicle
extracellular organelle
extracellular membrane−bounded organelle
extracellular exosome
endopeptidase Clp complex
cytoplasmic region
chromoplast
chloroplastic endopeptidase Clp complex
cell cortex
zinc ion transmembrane transporter activity
pectinesterase inhibitor activity
oxidoreductase activity, acting on the aldehyde or oxo group of donors
oxidoreductase activity, acting on a sulfur group of donors
molecular function inhibitor activity
enzyme inhibitor activity
carbohydrate:proton symporter activity
carbohydrate:monoatomic cation symporter activity
antioxidant activity
acyltransferase activity, transferring groups other than amino−acyl groups
Valine, leucine and isoleucine degradation
Unclassified: metabolism
Ubiquinone and other terpenoid−quinone biosynthesis
Transcription factors
Purine metabolism
Porphyrin and chlorophyll metabolism
Polyketide biosynthesis proteins
Nucleotide excision repair
Mismatch repair
Metabolism of cofactors and vitamins
Homologous recombination
Enzymes with EC numbers
DNA replication proteins
DNA replication
Cytoskeleton proteins
Ascorbate and aldarate metabolism
alpha−Linolenic acid metabolism
G4.G5.G2.fd
seedling development
response to alcohol
response to abiotic stimulus
regulation of hormone levels
regulation of developmental process
regulation of biological quality
regulation of anatomical structure morphogenesis
plant epidermis development
hormone metabolic process
cell differentiation
signaling receptor activity
molecular transducer activity
anion binding
Metabolism of terpenoids and polyketides
0.01289761
G3.G2.G5.fd
response to virus
innate immune response
immune system process
immune response
immune effector process
dephosphorylation
defense response to virus
defense response to symbiont
defense response
cell death
peroxisome
microbody
nuclease activity
molecular function regulator activity
hydrolase activity, acting on ester bonds
hydrolase activity
enzyme regulator activity
endonuclease activity
Unclassified: metabolism
Enzymes with EC numbers
Carbohydrate metabolism

d

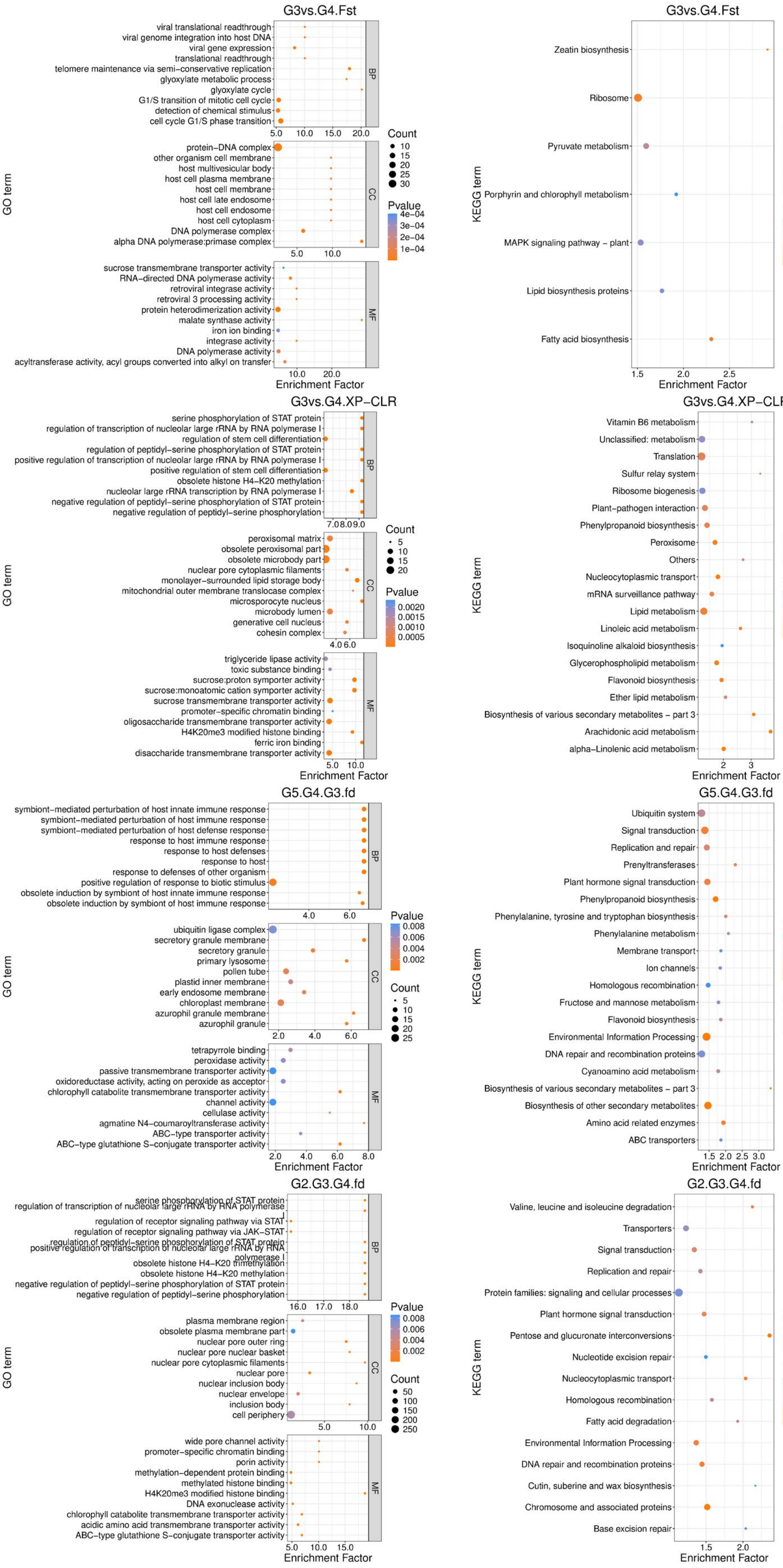
G3vs.G4.Fst
viral translational readthrough
viral genome integration into host DNA
viral gene expression
translational readthrough
telomere maintenance via semi−conservative replication
glyoxylate metabolic process
glyoxylate cycle
G1/S transition of mitotic cell cycle
detection of chemical stimulus
cell cycle G1/S phase transition
protein−DNA complex
other organism cell membrane
host multivesicular body
host cell plasma membrane
host cell membrane
host cell late endosome
host cell endosome
host cell cytoplasm
DNA polymerase complex
alpha DNA polymerase:primase complex
sucrose transmembrane transporter activity
RNA−directed DNA polymerase activity
retroviral integrase activity
retroviral 3 processing activity
protein heterodimerization activity
malate synthase activity
iron ion binding
integrase activity
DNA polymerase activity
acyltransferase activity, acyl groups converted into alkyl on transfer
BP
CC
MF
GO term
Enrichment Factor
Count
Pvalue
Zeatin biosynthesis
Ribosome
Pyruvate metabolism
Porphyrin and chlorophyll metabolism
MAPK signaling pathway − plant
Lipid biosynthesis proteins
Fatty acid biosynthesis
KEGG term
G3vs.G4.XP−CLR
serine phosphorylation of STAT protein
regulation of transcription of nucleolar large rRNA by RNA polymerase I
regulation of stem cell differentiation
regulation of peptidyl−serine phosphorylation of STAT protein
positive regulation of transcription of nucleolar large rRNA by RNA polymerase I
positive regulation of stem cell differentiation
obsolete histone H4−K20 methylation
nucleolar large rRNA transcription by RNA polymerase I
negative regulation of peptidyl−serine phosphorylation of STAT protein
negative regulation of peptidyl−serine phosphorylation
peroxisomal matrix
obsolete peroxisomal part
obsolete microbody part
nuclear pore cytoplasmic filaments
monolayer−surrounded lipid storage body
mitochondrial outer membrane translocase complex
microsporocyte nucleus
microbody lumen
generative cell nucleus
cohesin complex
triglyceride lipase activity
toxic substance binding
sucrose:proton symporter activity
sucrose:monoatomic cation symporter activity
sucrose transmembrane transporter activity
promoter−specific chromatin binding
oligosaccharide transmembrane transporter activity
H4K20me3 modified histone binding
ferric iron binding
disaccharide transmembrane transporter activity
Vitamin B6 metabolism
Unclassified: metabolism
Translation
Sulfur relay system
Ribosome biogenesis
Plant−pathogen interaction
Phenylpropanoid biosynthesis
Peroxisome
Others
Nucleocytoplasmic transport
mRNA surveillance pathway
Lipid metabolism
Linoleic acid metabolism
Isoquinoline alkaloid biosynthesis
Glycerophospholipid metabolism
Flavonoid biosynthesis
Ether lipid metabolism
Biosynthesis of various secondary metabolites − part 3
Arachidonic acid metabolism
alpha−Linolenic acid metabolism
G5.G4.G3.fd
symbiont−mediated perturbation of host innate immune response
symbiont−mediated perturbation of host immune response
symbiont−mediated perturbation of host defense response
response to host immune response
response to host defenses
response to host
response to defenses of other organism
positive regulation of response to biotic stimulus
obsolete induction by symbiont of host innate immune response
obsolete induction by symbiont of host immune response
ubiquitin ligase complex
secretory granule membrane
secretory granule
primary lysosome
pollen tube
plastid inner membrane
early endosome membrane
chloroplast membrane
azurophil granule membrane
azurophil granule
tetrapyrrole binding
peroxidase activity
passive transmembrane transporter activity
oxidoreductase activity, acting on peroxide as acceptor
chlorophyll catabolite transmembrane transporter activity
channel activity
cellulase activity
agmatine N4−coumaroyltransferase activity
ABC−type transporter activity
ABC−type glutathione S−conjugate transporter activity
Ubiquitin system
Signal transduction
Replication and repair
Prenyltransferases
Plant hormone signal transduction
Phenylpropanoid biosynthesis
Phenylalanine, tyrosine and tryptophan biosynthesis
Phenylalanine metabolism
Membrane transport
Ion channels
Homologous recombination
Fructose and mannose metabolism
Flavonoid biosynthesis
Environmental Information Processing
DNA repair and recombination proteins
Cyanoamino acid metabolism
Biosynthesis of various secondary metabolites − part 3
Biosynthesis of other secondary metabolites
Amino acid related enzymes
ABC transporters
G2.G3.G4.fd
serine phosphorylation of STAT protein
regulation of transcription of nucleolar large rRNA by RNA polymerase I
regulation of receptor signaling pathway via STAT
regulation of receptor signaling pathway via JAK−STAT
regulation of peptidyl−serine phosphorylation of STAT protein
positive regulation of transcription of nucleolar large rRNA by RNA polymerase I
obsolete histone H4−K20 trimethylation
obsolete histone H4−K20 methylation
negative regulation of peptidyl−serine phosphorylation of STAT protein
negative regulation of peptidyl−serine phosphorylation
plasma membrane region
obsolete plasma membrane part
nuclear pore outer ring
nuclear pore nuclear basket
nuclear pore cytoplasmic filaments
nuclear pore
nuclear inclusion body
nuclear envelope
inclusion body
cell periphery
wide pore channel activity
promoter−specific chromatin binding
porin activity
methylation−dependent protein binding
methylated histone binding
H4K20me3 modified histone binding
DNA exonuclease activity
chlorophyll catabolite transmembrane transporter activity
acidic amino acid transmembrane transporter activity
ABC−type glutathione S−conjugate transporter activity
Valine, leucine and isoleucine degradation
Transporters
Signal transduction
Replication and repair
Protein families: signaling and cellular processes
Plant hormone signal transduction
Pentose and glucuronate interconversions
Nucleotide excision repair
Nucleocytoplasmic transport
Homologous recombination
Fatty acid degradation
Environmental Information Processing
DNA repair and recombination proteins
Cutin, suberine and wax biosynthesis
Chromosome and associated proteins
Base excision repair

e

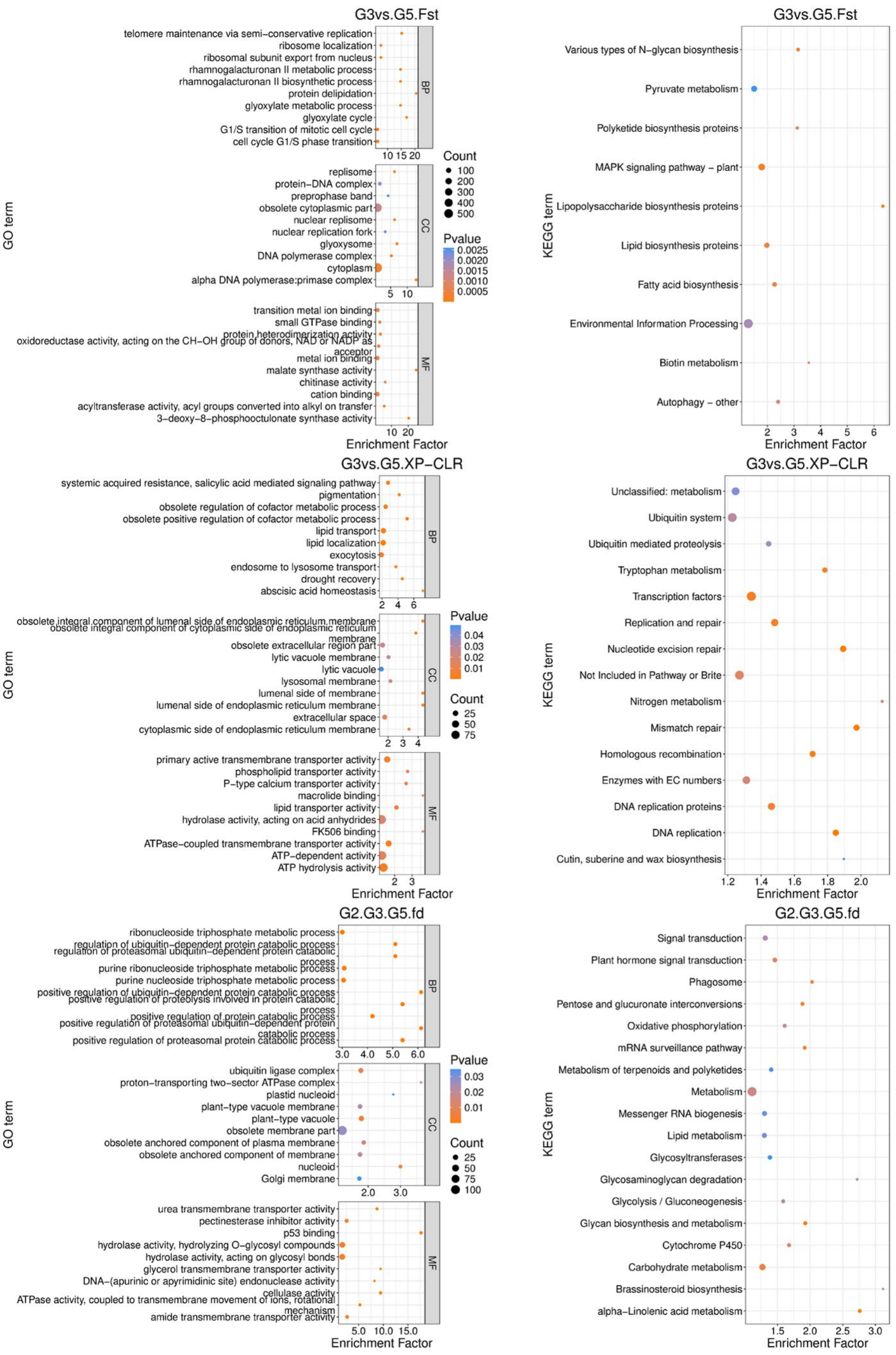

G3vs.G5.Fst
telomere maintenance via semi−conservative replication
ribosome localization
ribosomal subunit export from nucleus
rhamnogalacturonan II metabolic process
rhamnogalacturonan II biosynthetic process
protein delipidation
glyoxylate metabolic process
glyoxylate cycle
G1/S transition of mitotic cell cycle
cell cycle G1/S phase transition
replisome
protein−DNA complex
preprophase band
obsolete cytoplasmic part
nuclear replisome
nuclear replication fork
glyoxysome
DNA polymerase complex
cytoplasm
alpha DNA polymerase:primase complex
transition metal ion binding
small GTPase binding
protein heterodimerization activity
oxidoreductase activity, acting on the CH−OH group of donors, NAD or NADP as acceptor
metal ion binding
malate synthase activity
chitinase activity
cation binding
acyltransferase activity, acyl groups converted into alkyl on transfer
3−deoxy−8−phosphooctulonate synthase activity
BP
CC
MF
GO term
Enrichment Factor
Count
Pvalue
Various types of N−glycan biosynthesis
Pyruvate metabolism
Polyketide biosynthesis proteins
MAPK signaling pathway − plant
Lipopolysaccharide biosynthesis proteins
Lipid biosynthesis proteins
Fatty acid biosynthesis
Environmental Information Processing
Biotin metabolism
Autophagy − other
KEGG term
G3vs.G5.XP−CLR
systemic acquired resistance, salicylic acid mediated signaling pathway
pigmentation
obsolete regulation of cofactor metabolic process
obsolete positive regulation of cofactor metabolic process
lipid transport
lipid localization
exocytosis
endosome to lysosome transport
drought recovery
abscisic acid homeostasis
obsolete integral component of lumenal side of endoplasmic reticulum membrane
obsolete integral component of cytoplasmic side of endoplasmic reticulum membrane
obsolete extracellular region part
lytic vacuole membrane
lytic vacuole
lysosomal membrane
lumenal side of membrane
lumenal side of endoplasmic reticulum membrane
extracellular space
cytoplasmic side of endoplasmic reticulum membrane
primary active transmembrane transporter activity
phospholipid transporter activity
P−type calcium transporter activity
macrolide binding
lipid transporter activity
hydrolase activity, acting on acid anhydrides
FK506 binding
ATPase−coupled transmembrane transporter activity
ATP−dependent activity
ATP hydrolysis activity
Unclassified: metabolism
Ubiquitin system
Ubiquitin mediated proteolysis
Tryptophan metabolism
Transcription factors
Replication and repair
Nucleotide excision repair
Not Included in Pathway or Brite
Nitrogen metabolism
Mismatch repair
Homologous recombination
Enzymes with EC numbers
DNA replication proteins
DNA replication
Cutin, suberine and wax biosynthesis
G2.G3.G5.fd
ribonucleoside triphosphate metabolic process
regulation of ubiquitin−dependent protein catabolic process
regulation of proteasomal ubiquitin−dependent protein catabolic process
purine ribonucleoside triphosphate metabolic process
purine nucleoside triphosphate metabolic process
positive regulation of ubiquitin−dependent protein catabolic process
positive regulation of proteolysis involved in protein catabolic process
positive regulation of protein catabolic process
positive regulation of proteasomal ubiquitin−dependent protein catabolic process
positive regulation of proteasomal protein catabolic process
ubiquitin ligase complex
proton−transporting two−sector ATPase complex
plastid nucleoid
plant−type vacuole membrane
plant−type vacuole
obsolete membrane part
obsolete anchored component of plasma membrane
obsolete anchored component of membrane
nucleoid
Golgi membrane
urea transmembrane transporter activity
pectinesterase inhibitor activity
p53 binding
hydrolase activity, hydrolyzing O−glycosyl compounds
hydrolase activity, acting on glycosyl bonds
glycerol transmembrane transporter activity
DNA−(apurinic or apyrimidinic site) endonuclease activity
cellulase activity
ATPase activity, coupled to transmembrane movement of ions, rotational mechanism
amide transmembrane transporter activity
Signal transduction
Plant hormone signal transduction
Phagosome
Pentose and glucuronate interconversions
Oxidative phosphorylation
mRNA surveillance pathway
Metabolism of terpenoids and polyketides
Metabolism
Messenger RNA biogenesis
Lipid metabolism
Glycosyltransferases
Glycosaminoglycan degradation
Glycolysis / Gluconeogenesis
Glycan biosynthesis and metabolism
Cytochrome P450
Carbohydrate metabolism
Brassinosteroid biosynthesis
alpha−Linolenic acid metabolism

f

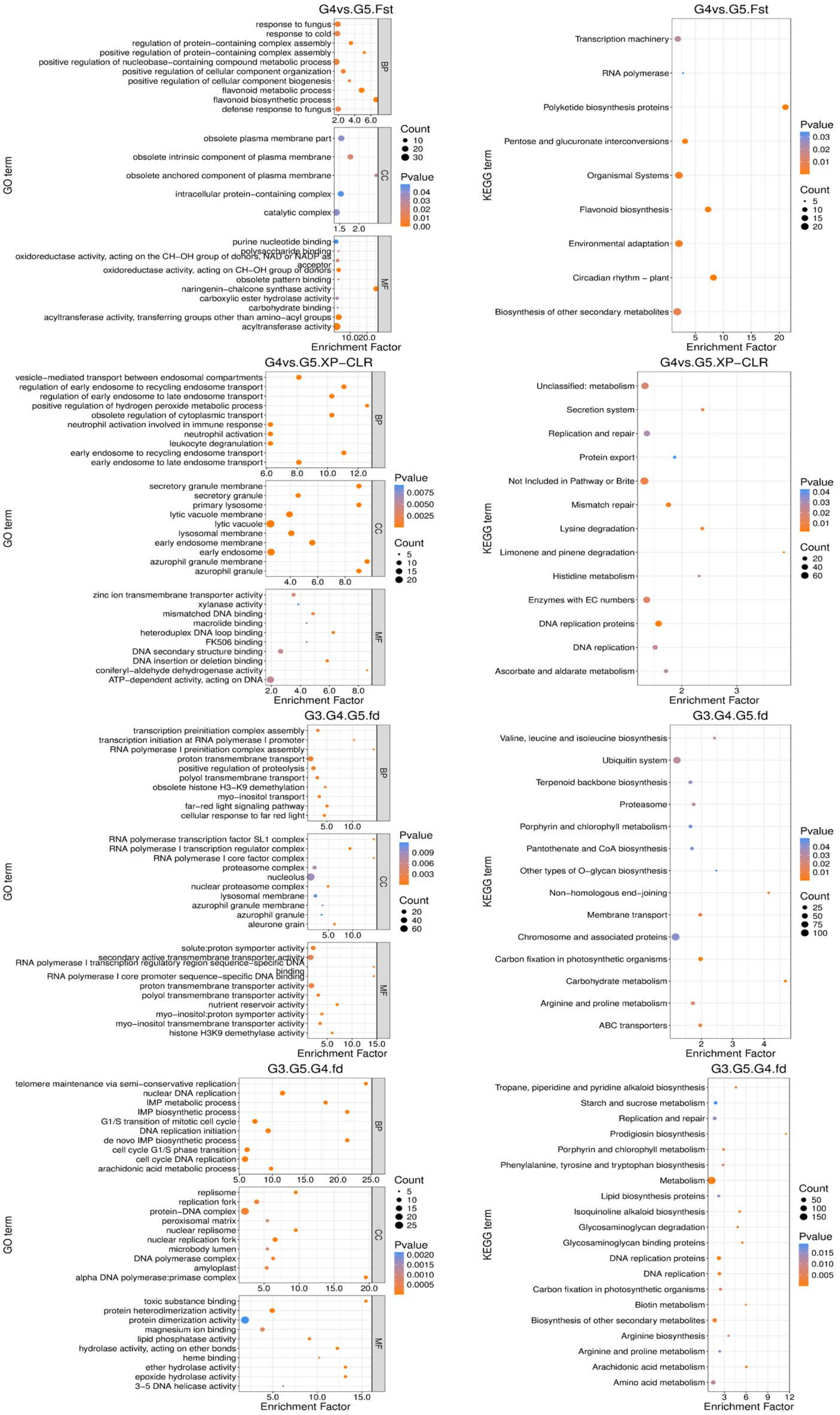

Supplementary Fig. 19 Enrichment analyses of significantly selected and introgression among different populations. Gene Ontology (GO) and Kyoto Encyclopedia of Genes and Genomes (KEGG) enrichment were conducted for genes located in the top 1% of selected and introgression regions among the following groups: **a,** G2-G3; **b,** G2-G4; **c,** G2-G5; **d,** G3-G4; **e,** G3-G5, and **f,** G4-G5. *p-value* < 0.05.

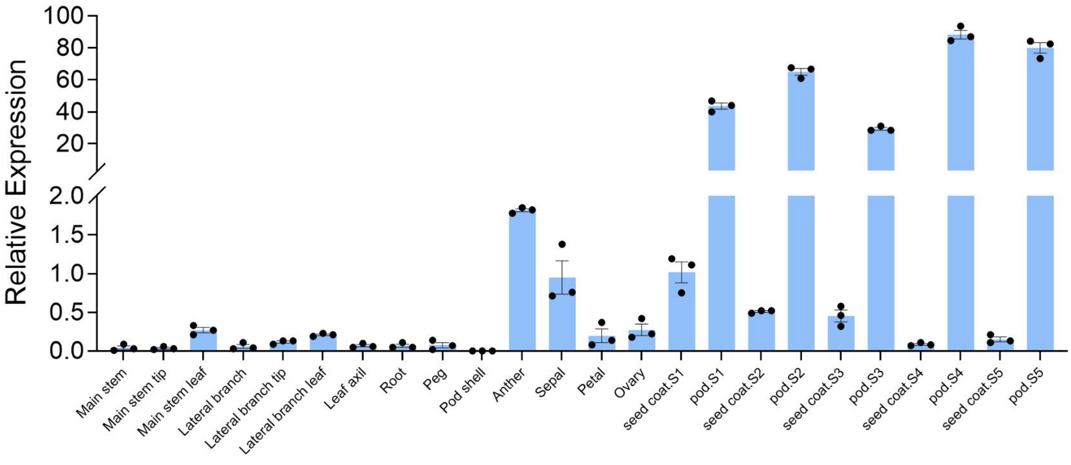

Supplementary Fig. 20 qRTPCR result of tissue-specific expression of *AhWRI1* gene. S1-S5: 15, 30, 45, 60, 75 days after flowering. Quantitative data are mean ± s.e.m. n = 3 biologically independent samples.

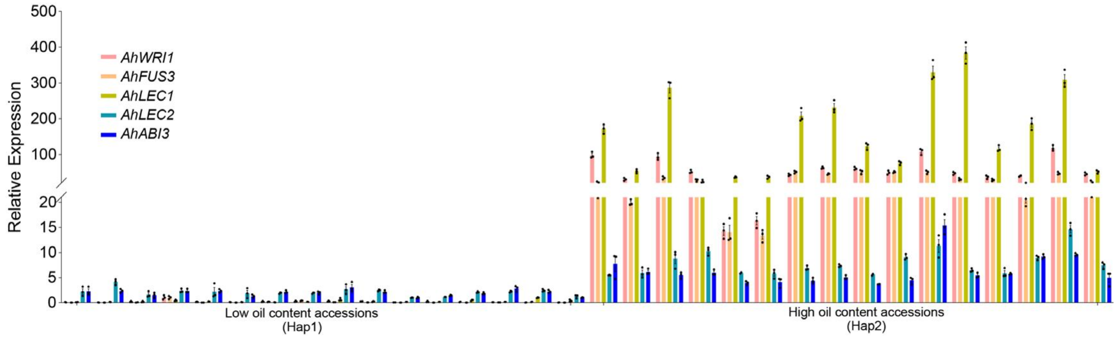

Supplementary Fig. 21 The expression levels of *AhFUS3*, *AhABI3*, *AhLECs*, and their target gene *AhWRI1* in high and low oil content peanut accessions. Quantitative data are mean ± s.e.m. n = 3 biologically independent samples.

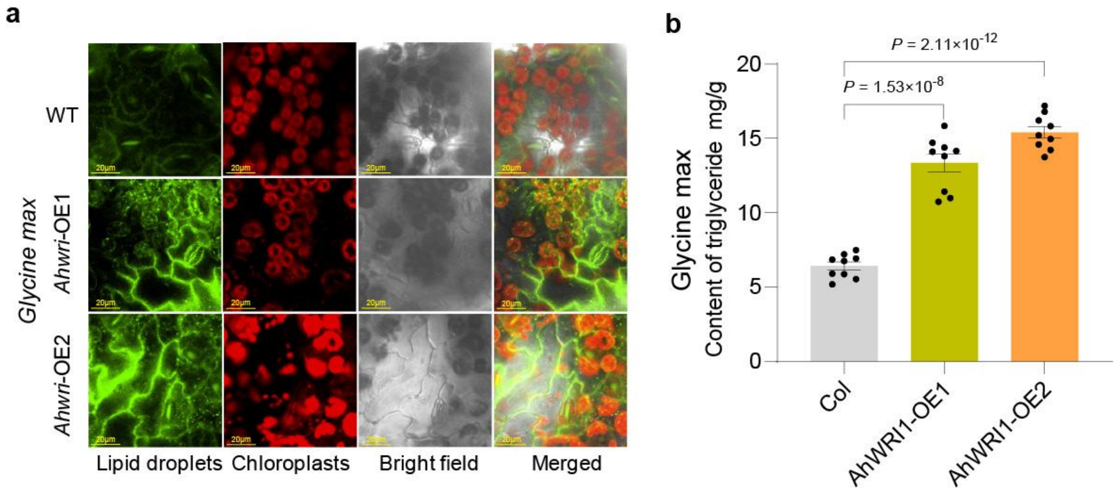

Supplementary Fig. 22 The oil content identification of overexpressing *AhWRI1* in *Glycine max* plant. a, Confocal images of accumulated lipid droplets (LDs) stained with Nile Red (green) in *Glycine max* transgenic plant leaf infected with Agrobacterium. Scale bar corresponds to 20 μm. **b,** Triacylglycerols content of *Glycine max* plant seeds transiently overexpressing *AhWRI1* isoforms. Quantitative data are mean ± s.e.m. n = 9 biologically independent samples. *P* values were calculated by two-tailed Student's t-tests.

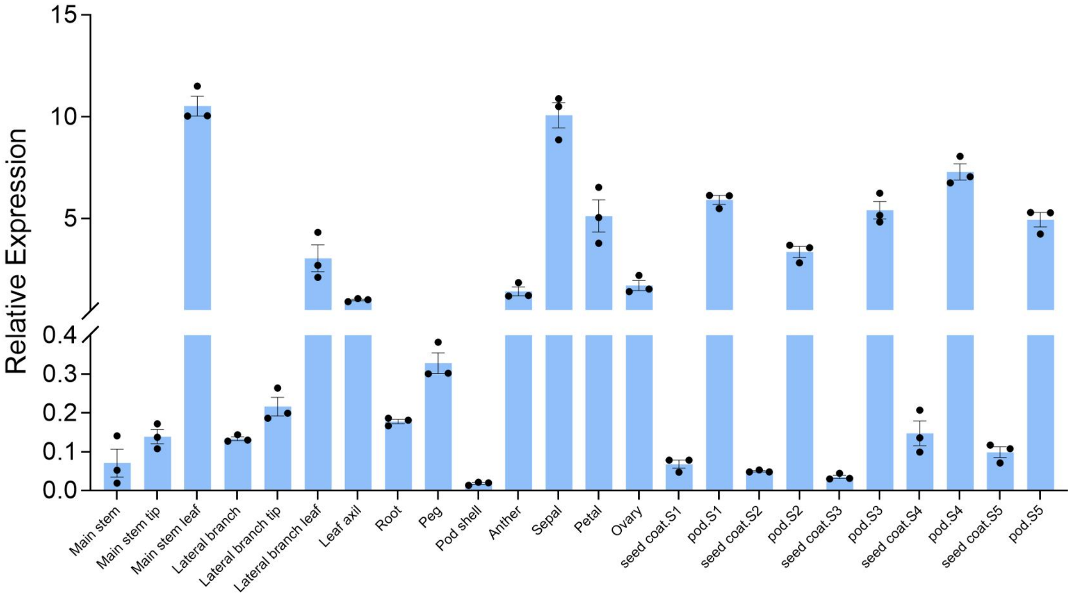

Supplementary Fig. 23 Tissue-specific expression of *AhGSA1* gene. S1-S5: 15, 30, 45, 60, 75 days after flowering. Quantitative data are mean ± s.e.m. n = 3 biologically independent samples.

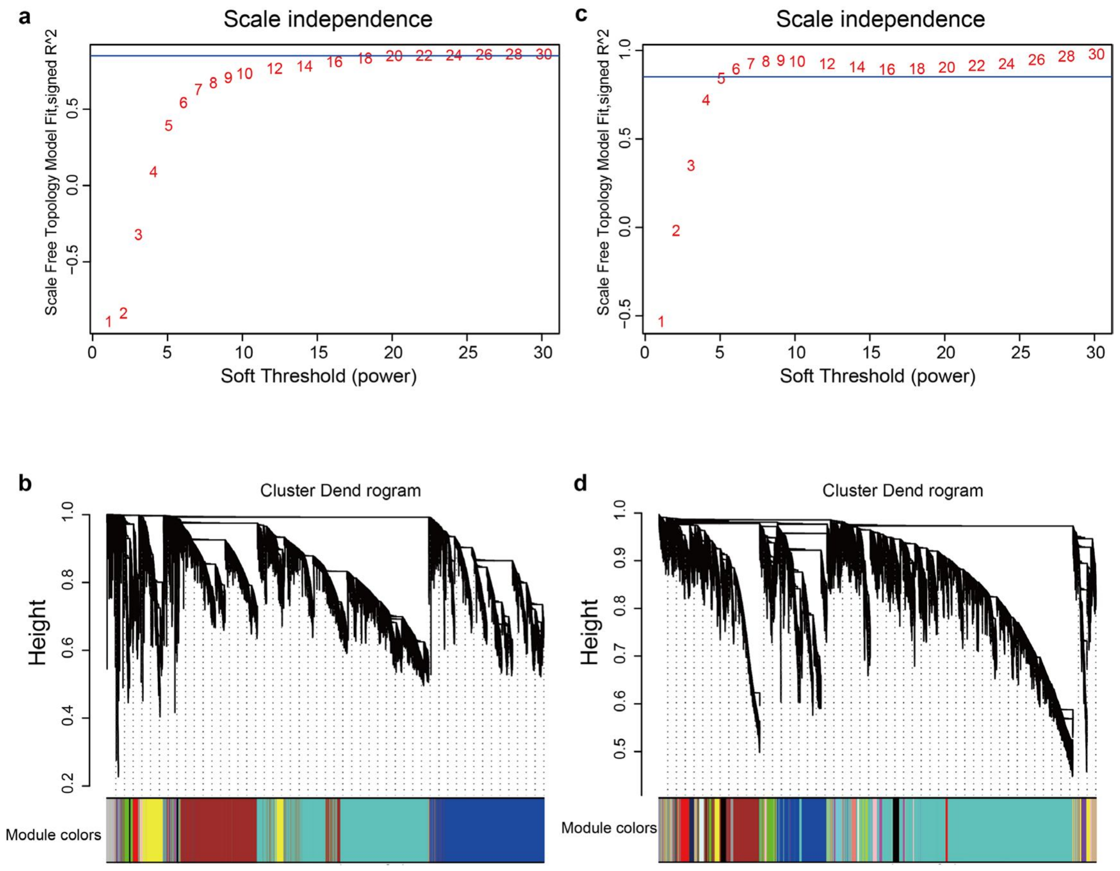

Supplementary Fig. 24 WGCNA analysis and network construction. a-b, Evaluation of scale-free fit index at different soft threshold powers. (**a**) Lipid. (**b**) Anthocyanidin. **c-d,** A hierarchical clustering tree displays the coexpression modules identified by WGCNA, together with the assigned merged module colors. (**c**) Lipid. (**d**) Anthocyanidin.

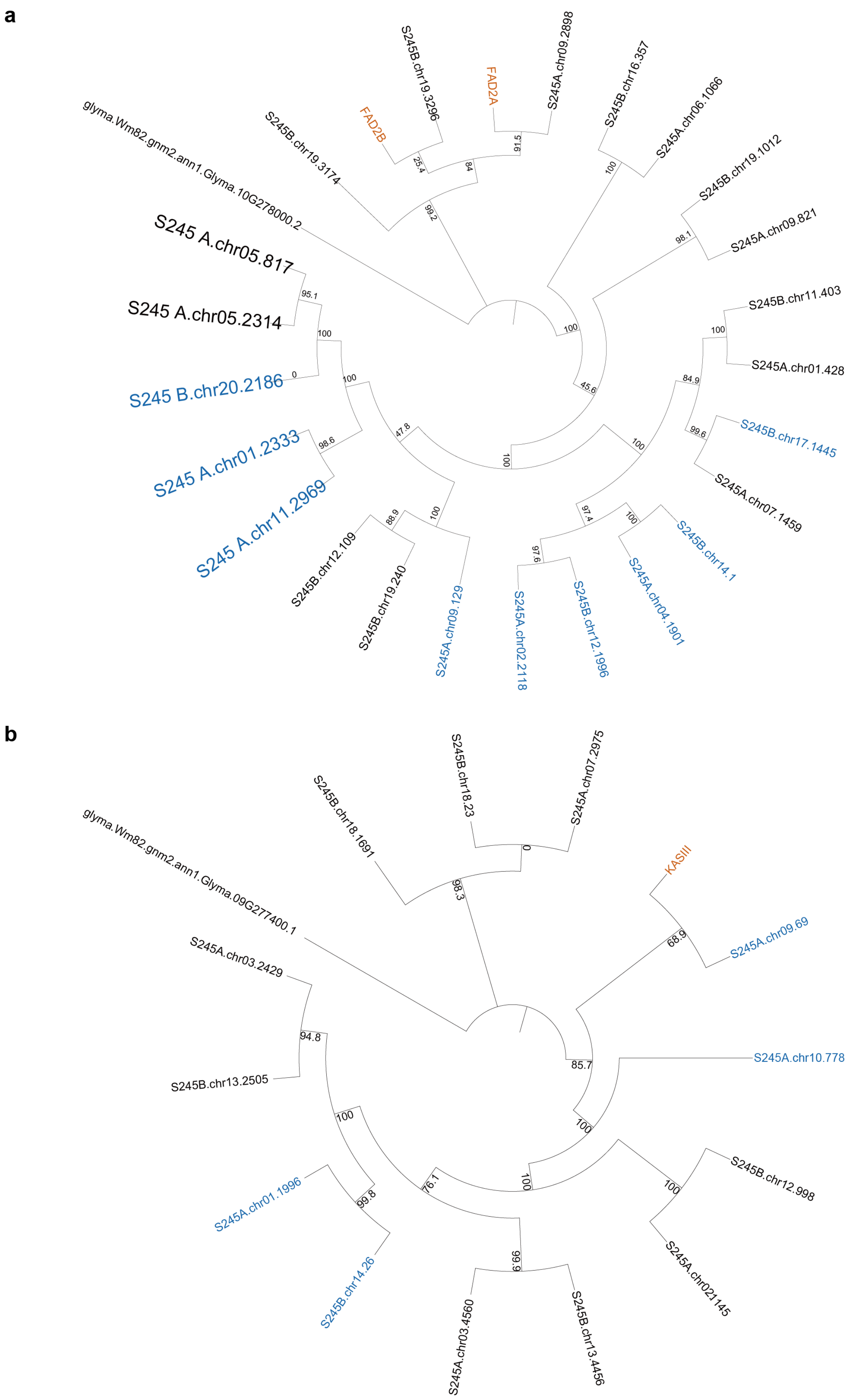
a
S245B.chr19.3296
FAD2B
FAD2A
S245A.chr09.2898
S245B.chr16.357
S245A.chr06.1066
S245B.chr19.1012
S245A.chr09.821
S245B.chr19.3174
glyma.Wm82.gnm2.ann1.Glyma.10G278000.2
S245 A.chr05.817
S245 A.chr05.2314
S245 B.chr20.2186
S245 A.chr01.2333
S245 A.chr11.2969
S245B.chr12.109
S245B.chr19.240
S245A.chr09.129
S245A.chr02.2118
S245B.chr12.1996
S245A.chr04.1901
S245B.chr14.1
S245A.chr07.1459
S245B.chr17.1445
S245B.chr11.403
S245A.chr01.428
b
S245B.chr18.23
S245A.chr07.2975
S245B.chr18.1691
KASIII
S245A.chr09.69
glyma.Wm82.gnm2.ann1.Glyma.09G277400.1
S245A.chr03.2429
S245B.chr13.2505
S245A.chr10.778
S245A.chr01.1996
S245B.chr14.26
S245A.chr03.4560
S245B.chr13.4456
S245A.chr021145
S245B.chr12.998

c

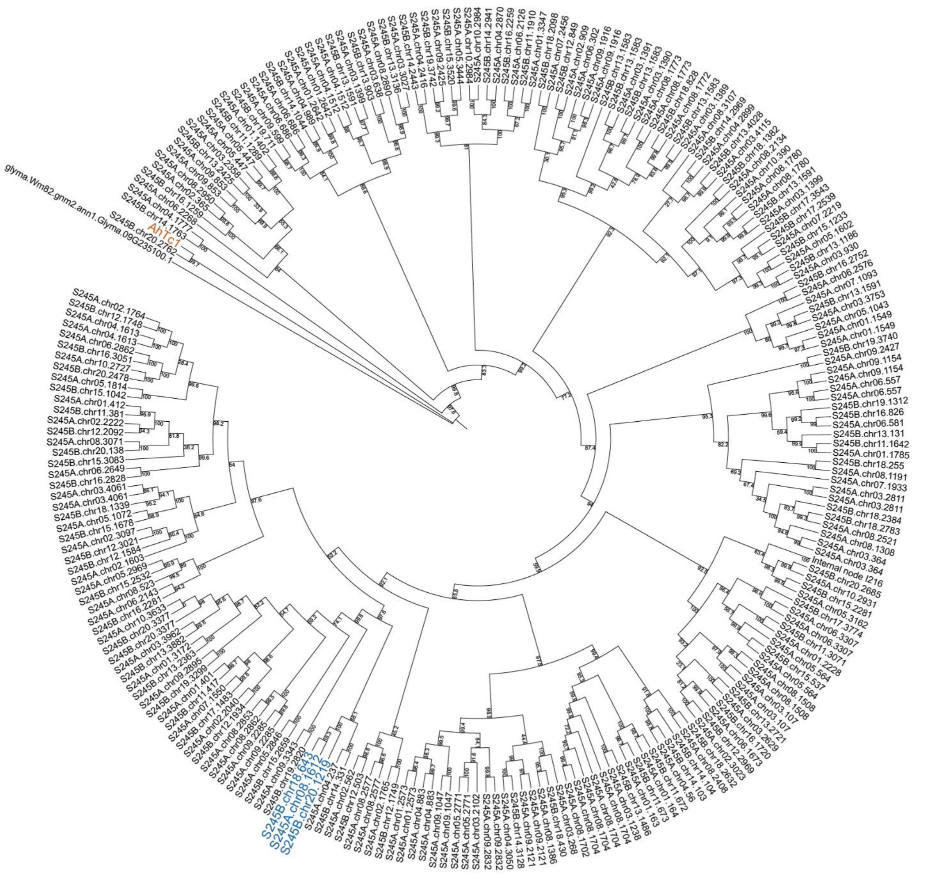

d

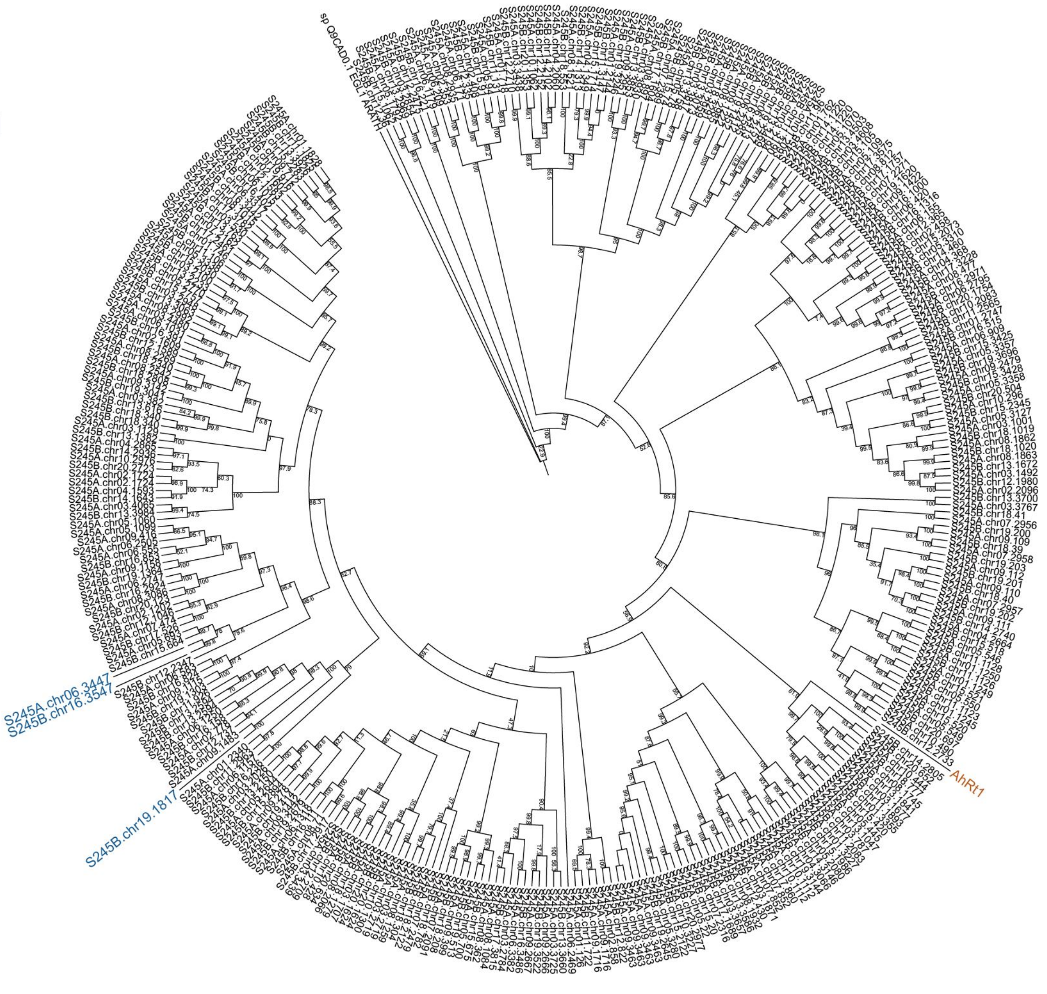

Supplementary Fig. 25 Evolutionary tree of transcription factors and enzymes in the peanut genome. a, The tree of FAD genes. **b,** The tree of KAS genes. **c,** The tree of MYB genes. **d,** The tree of bHLH genes. The outgroup of the bHLH tree is the sequence of *Arabidopsis*, and the outgroups of the other trees are soybean. Orange represents the relevant genes that have been verified in peanuts, and blue represents the genes related to anthocyanins and lipids that we screened out.

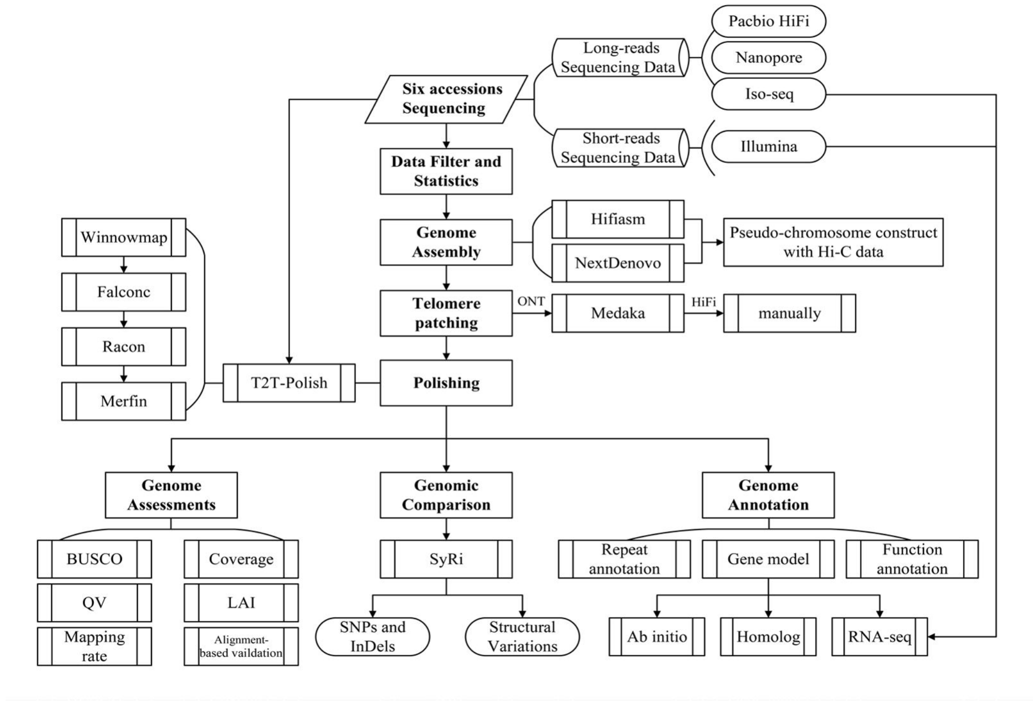

Supplementary Fig. 26 The flowchart of genome assembly.

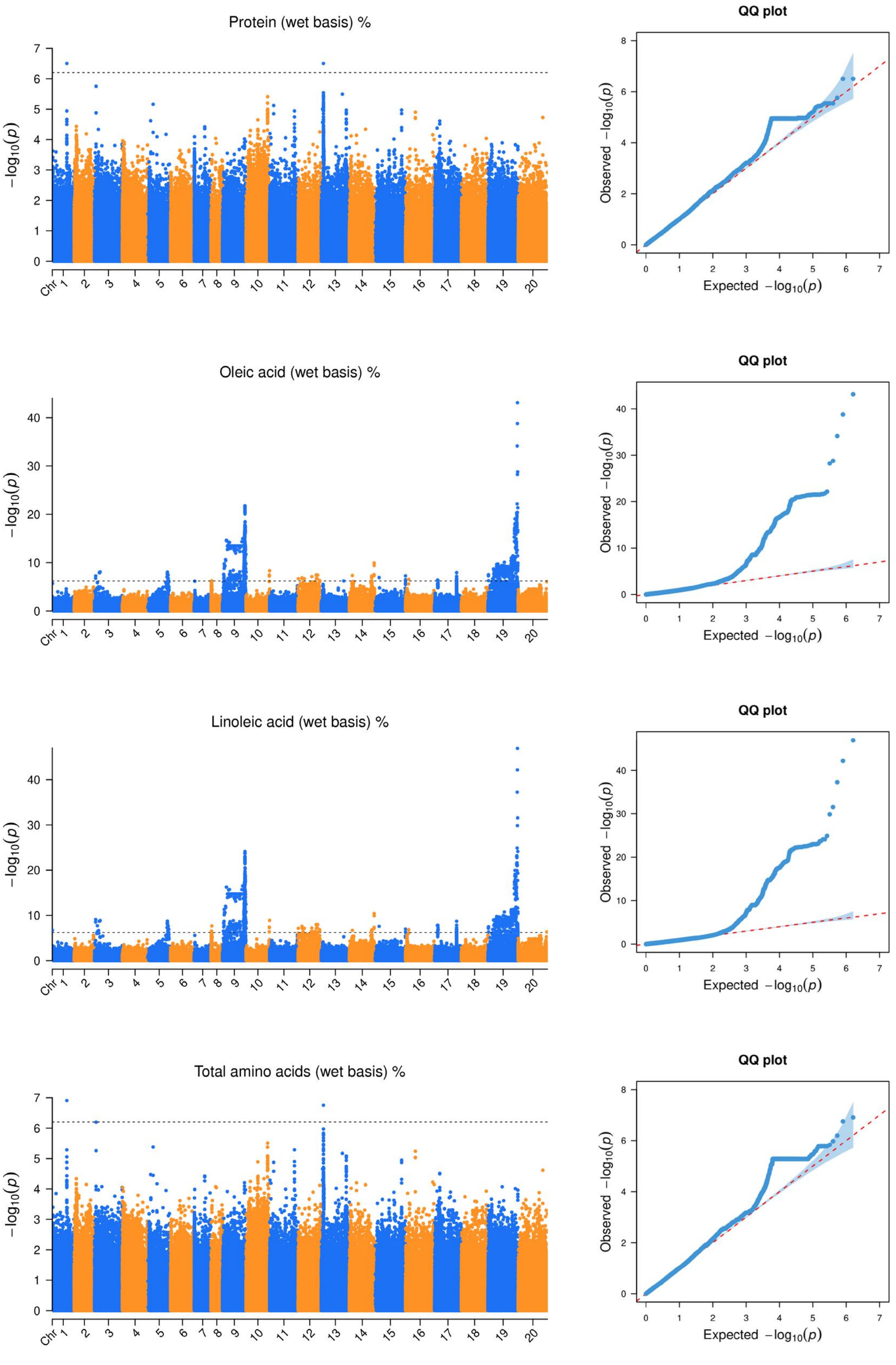

Protein (wet basis) %
Oleic acid (wet basis) %
Linoleic acid (wet basis) %
Total amino acids (wet basis) %
QQ plot
$-\log_{10}(p)$
Chr
Observed $-\log_{10}(p)$
Expected $-\log_{10}(p)$

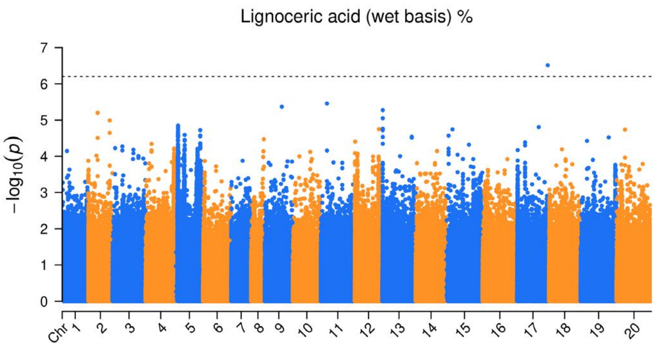
Lignoceric acid (wet basis) %
$-\log_{10}(p)$
Chr 1 2 3 4 5 6 7 8 9 10 11 12 13 14 15 16 17 18 19 20

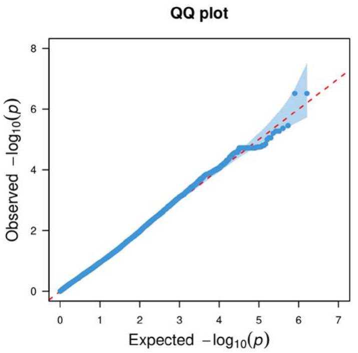
QQ plot
Observed $-\log_{10}(p)$
Expected $-\log_{10}(p)$

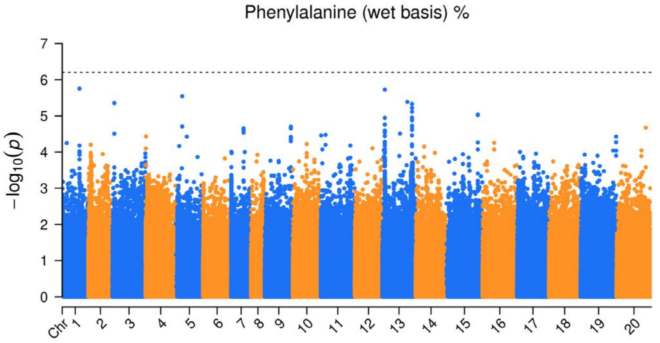
Phenylalanine (wet basis) %
$-\log_{10}(p)$
Chr 1 2 3 4 5 6 7 8 9 10 11 12 13 14 15 16 17 18 19 20

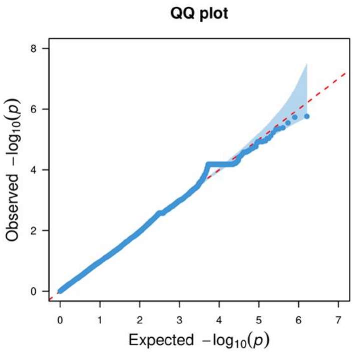
QQ plot
Observed $-\log_{10}(p)$
Expected $-\log_{10}(p)$

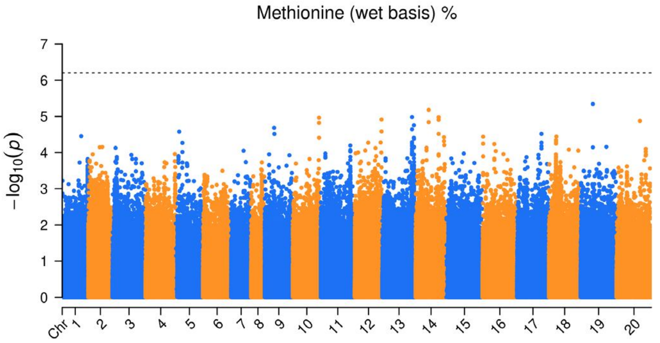
Methionine (wet basis) %
$-\log_{10}(p)$
Chr 1 2 3 4 5 6 7 8 9 10 11 12 13 14 15 16 17 18 19 20

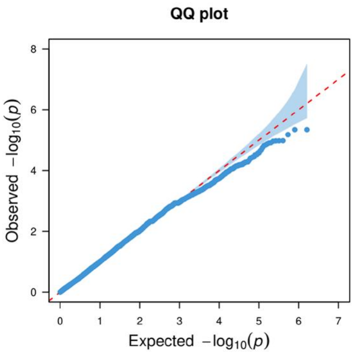
QQ plot
Observed $-\log_{10}(p)$
Expected $-\log_{10}(p)$

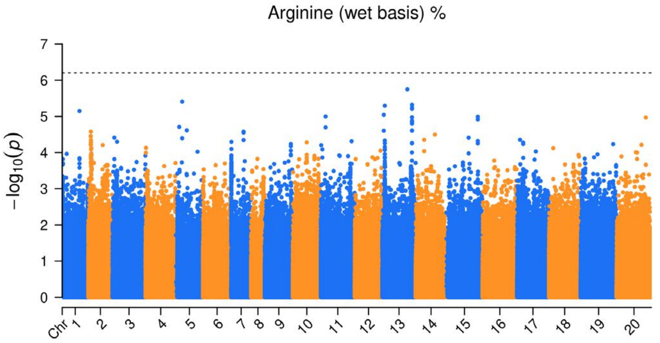
Arginine (wet basis) %
$-\log_{10}(p)$
Chr 1 2 3 4 5 6 7 8 9 10 11 12 13 14 15 16 17 18 19 20

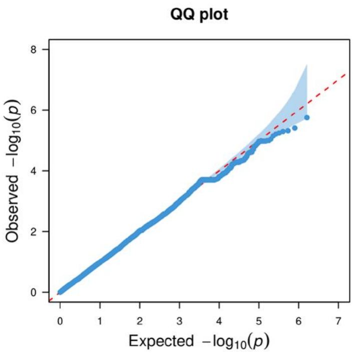
QQ plot
Observed $-\log_{10}(p)$
Expected $-\log_{10}(p)$

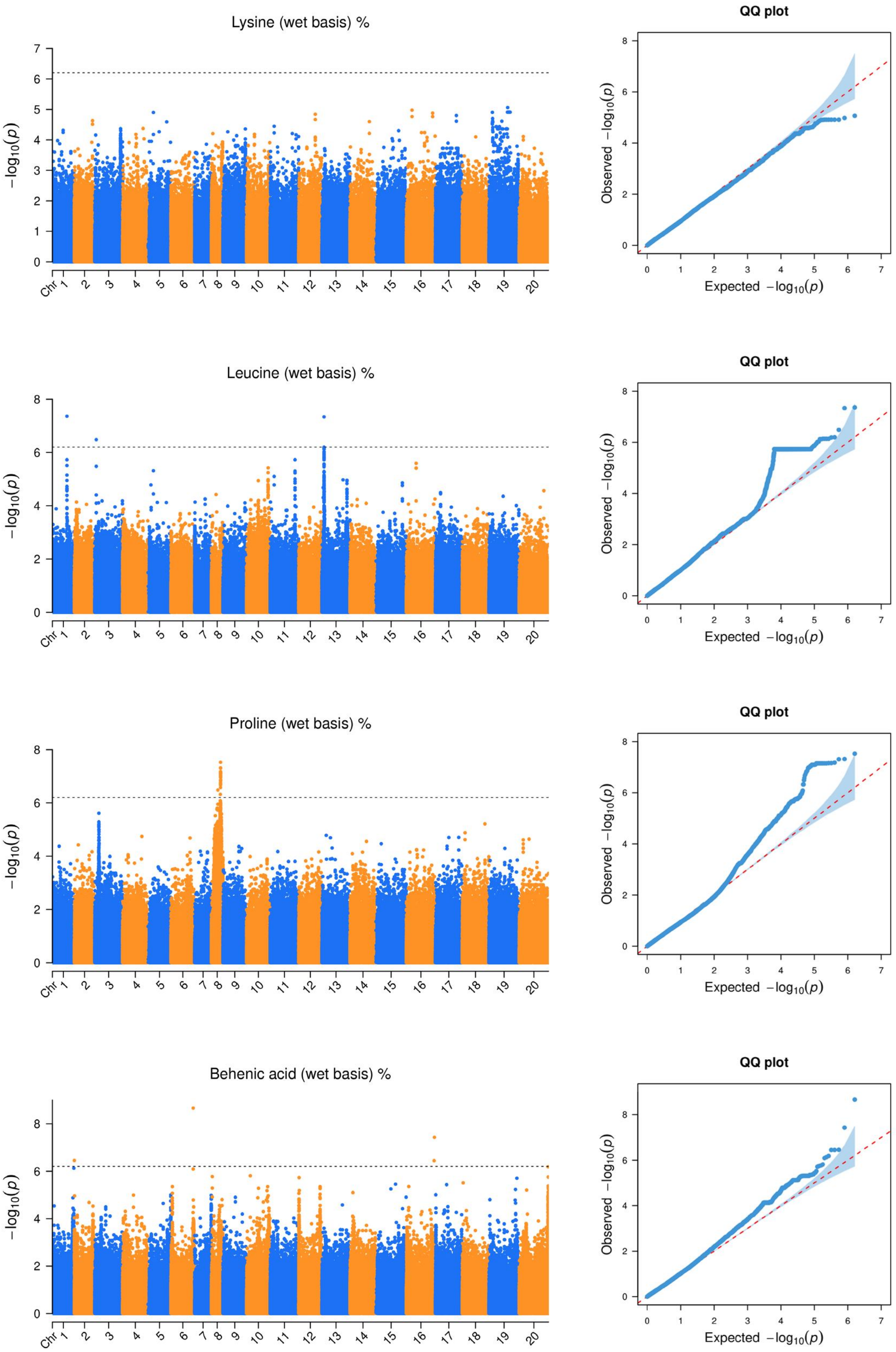

Lysine (wet basis) %
Leucine (wet basis) %
Proline (wet basis) %
Behenic acid (wet basis) %
$-\log_{10}(p)$
Chr 1 2 3 4 5 6 7 8 9 10 11 12 13 14 15 16 17 18 19 20
QQ plot
Observed $-\log_{10}(p)$
Expected $-\log_{10}(p)$

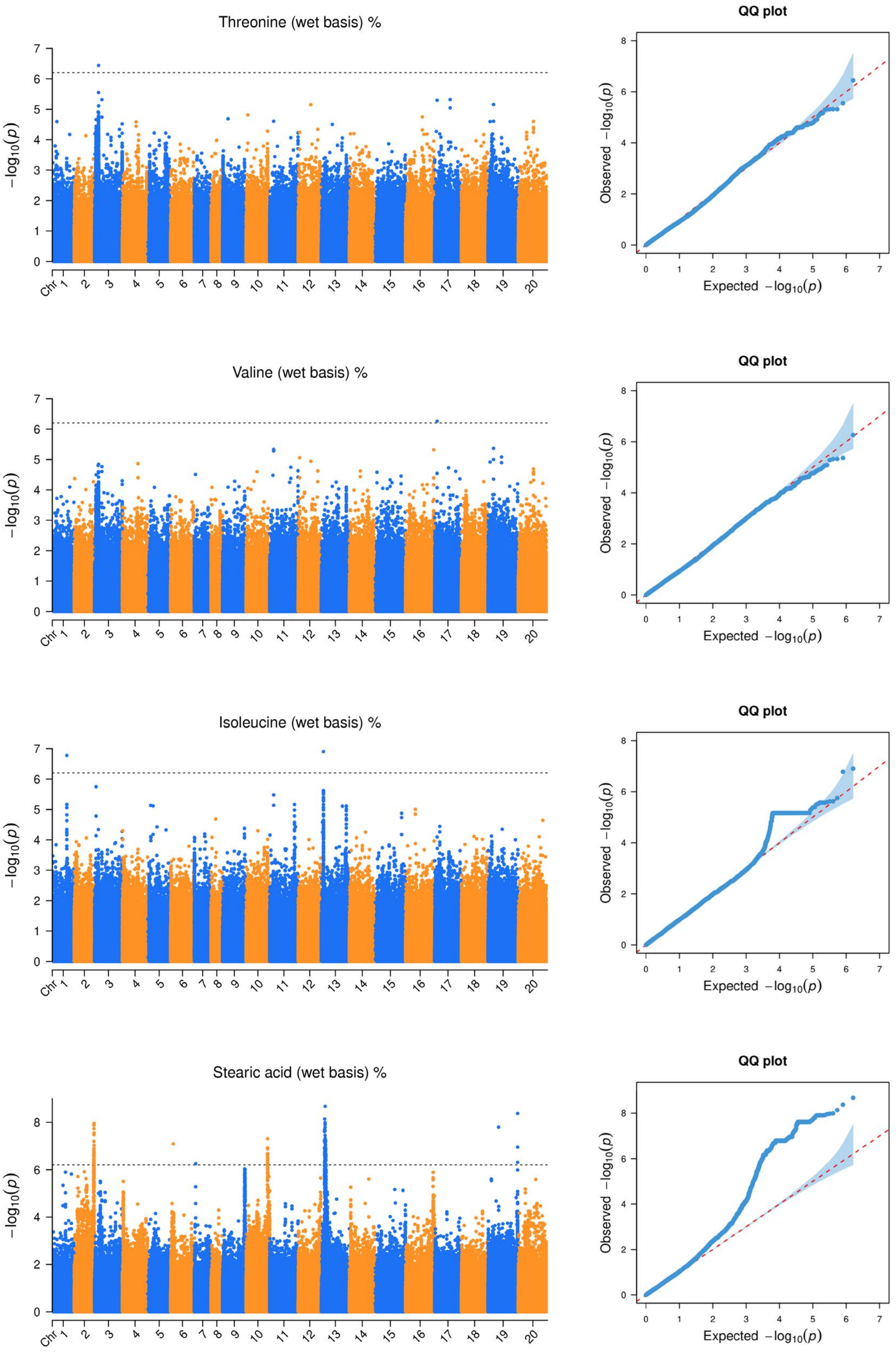
Threonine (wet basis) %
QQ plot
Valine (wet basis) %
QQ plot
Isoleucine (wet basis) %
QQ plot
Stearic acid (wet basis) %
QQ plot
$-\log_{10}(p)$
Chr 1 2 3 4 5 6 7 8 9 10 11 12 13 14 15 16 17 18 19 20
Observed $-\log_{10}(p)$
Expected $-\log_{10}(p)$

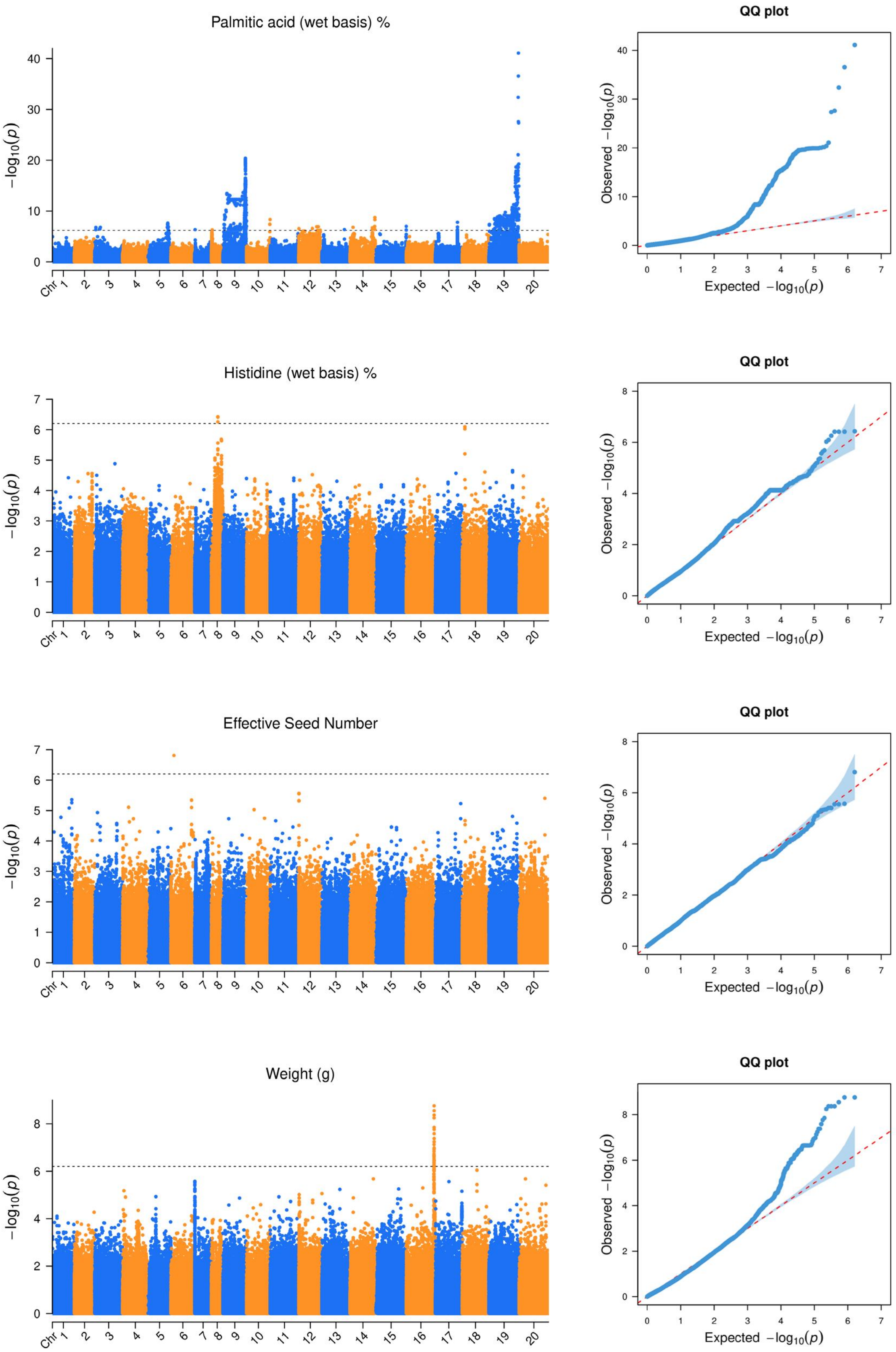
Palmitic acid (wet basis) %
QQ plot
Histidine (wet basis) %
QQ plot
Effective Seed Number
QQ plot
Weight (g)
QQ plot
$-\log_{10}(p)$
Chr
Observed $-\log_{10}(p)$
Expected $-\log_{10}(p)$

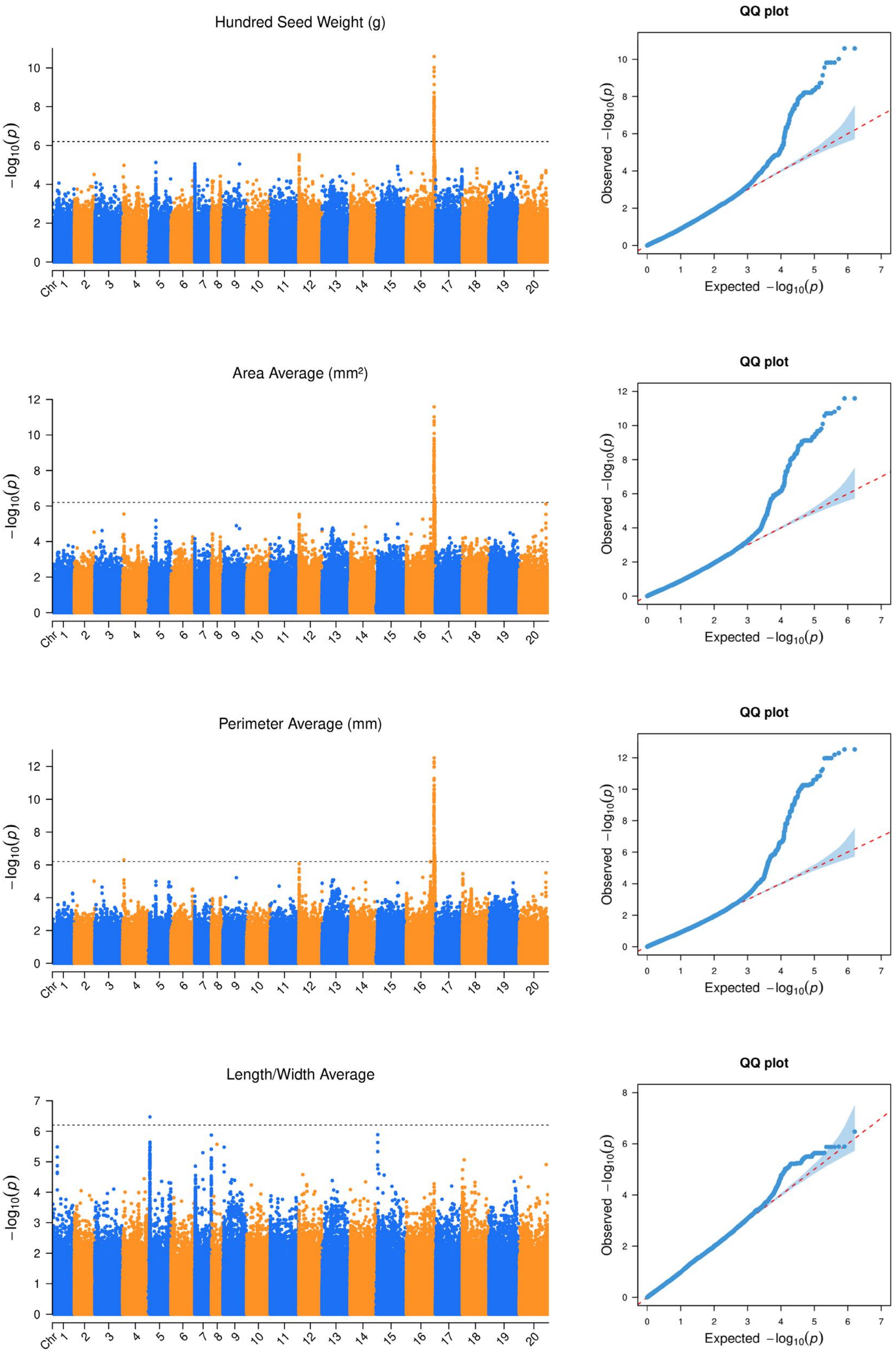

Hundred Seed Weight (g)
Area Average (mm²)
Perimeter Average (mm)
Length/Width Average
$-\log_{10}(p)$
Chr 1 2 3 4 5 6 7 8 9 10 11 12 13 14 15 16 17 18 19 20
QQ plot
Observed $-\log_{10}(p)$
Expected $-\log_{10}(p)$

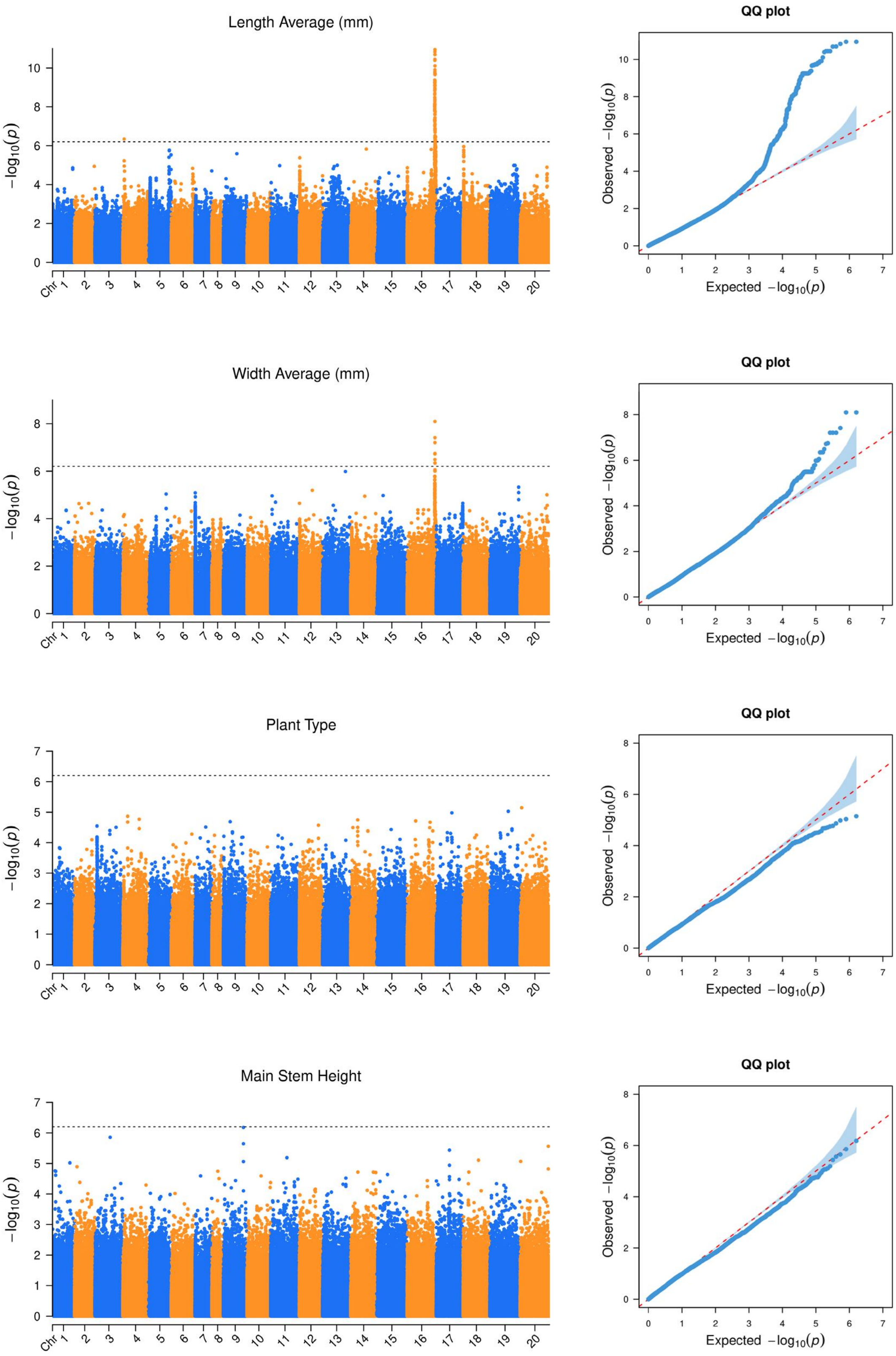

Length Average (mm)
QQ plot
Width Average (mm)
QQ plot
Plant Type
QQ plot
Main Stem Height
QQ plot
$-\log_{10}(p)$
Chr 1 2 3 4 5 6 7 8 9 10 11 12 13 14 15 16 17 18 19 20
Observed $-\log_{10}(p)$
Expected $-\log_{10}(p)$

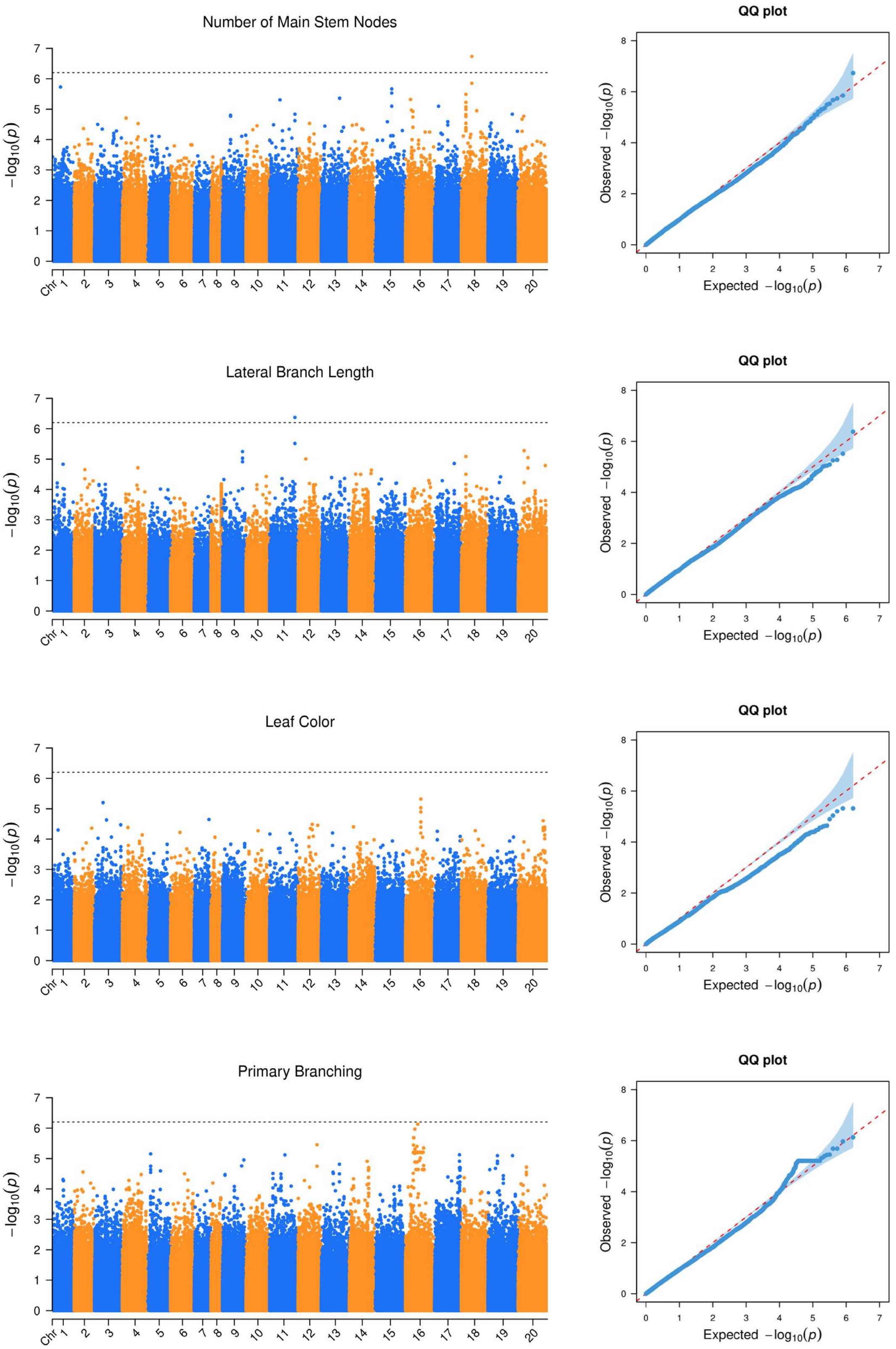
Number of Main Stem Nodes
QQ plot
Lateral Branch Length
QQ plot
Leaf Color
QQ plot
Primary Branching
QQ plot
$-\log_{10}(p)$
Chr
Observed $-\log_{10}(p)$
Expected $-\log_{10}(p)$

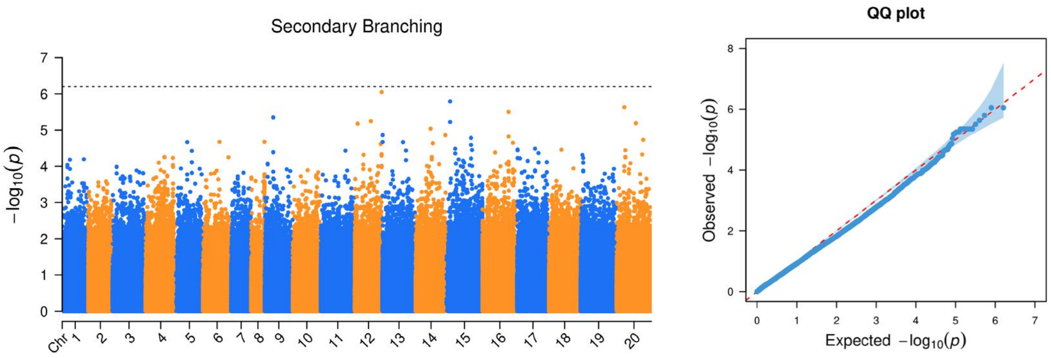

Supplementary Fig. 27 GWAS for 33 traits. In each Fig, the left side depicts the Manhattan plot, while the right side represents the QQ plot. The horizontal dashed line indicates the significant threshold (-log 10(*P*)*; P* = 1/SNPs).

References

1. Bertioli, D. J. *et al.* The genome sequence of segmental allotetraploid peanut *Arachis hypogaea*. *Nat Genet.* **51**, 877-884 (2019).

2. Bertioli, D. J. *et al.* The genome sequences of *Arachis duranensis* and *Arachis ipaensis*, the diploid ancestors of cultivated peanut. *Nat Genet.* **48**, 438-446 (2016).

3. Zhao, K. *et al.* Pangenome analysis reveals structural variation associated with seed size and weight traits in peanut. *Nat Genet.* **57**, 1250-1261 (2025).

4. Wang X, *et al.* A telomere-to-telomere genome assembly of the cultivated peanut. *Mol Plant.* **18**, 5-8 (2025).

Supplementary Notes

Candidate gene for Kernel dehydration rate (KDR) in peanut

Due to the subterranean nature of peanut, its seeds tend to have a high moisture content. Therefore, post-harvest, the seeds must be rapidly dried and maintained at a specific moisture level for proper storage, as excessive moisture may lead to germination or mold formation. This is particularly problematic under warm and humid conditions, which may also promote aflatoxin contamination. Therefore, we conducted a genome-wide association study (GWAS) using seed moisture content as the phenotypic trait. A significant signal was identified at the end of chromosome 08 (chr08: 53,353,397) (Supplementary Notes Fig.1a, b). Functional annotation of genes within a 200kb region surrounding this locus revealed two consecutive genes, chr08.2534 and chr08.2535, which were annotated as Ethylene-insensitive proteins (*EIN2*). These genes were designated as *Ah2534_EIN2* and *Ah2535_EIN2*. Previous studies have demonstrated that *EIN2* plays a critical role in ethylene signaling, influencing various developmental processes and responses to multiple stresses, including nutrient deficiency, metal ion stress, plant immunity, and abiotic stressors[5]. Furthermore, Yu et al. (2025) identified a causal micropeptide, microqKDR1, in maize that regulates kernel dehydration by modulating the expression of *EIL1/EIL3*[6]. Knockout experiments involving *ZmEIL1* and *ZmEIL3* showed that KDR levels were significantly reduced in the knockout lines compared to the wild-type, providing further evidence of the involvement of the ethylene signaling pathway in kernel dehydration. Gene structure analysis shows that the peak is located in the promoter region of the *Ah2534_EIN2* gene, 1,073bp upstream of the ATG, where a population-level variation (G to A substitution) was found. Further analysis of the exonic region reveals another population-level variation (C to T substitution) at the fifth exon, which results in an amino acid change from histidine (His) to tyrosine (Tyr). We performed a haplotype analysis of the gene, dividing it into three haplotypes: Hap1 (GC), Hap2 (GT), and Hap3 (AC) (Supplementary Notes Fig.1c). In the population analysis, these three haplotypes contained 171, 62, and 240 accessions, respectively, with corresponding average moisture

contents of 5.60%, 5.54%, and 5.74%. Significance analysis indicated that the moisture content difference between Hap1 and Hap2 was not significant (P = 0.47), while Hap3 showed a significantly higher moisture content than both Hap1 ($P = 7.63 \times 10^{-3}$) and Hap2 ($P = 3.76 \times 10^{-3}$) (Supplementary Notes Fig.1d). No population-level variation was found in the gene structure of *Ah2535_EIN2*. Based on these results, we infer that the *Ah2534_EIN2* gene plays a crucial role in regulating the dehydration rate (KDR) and moisture content in peanuts. Among the three haplotypes, Hap2, which exhibited lower moisture content, has significant implications for the subsequent breeding of peanuts with high KDR and low moisture content, which may help reduce the risk of aflatoxin contamination.

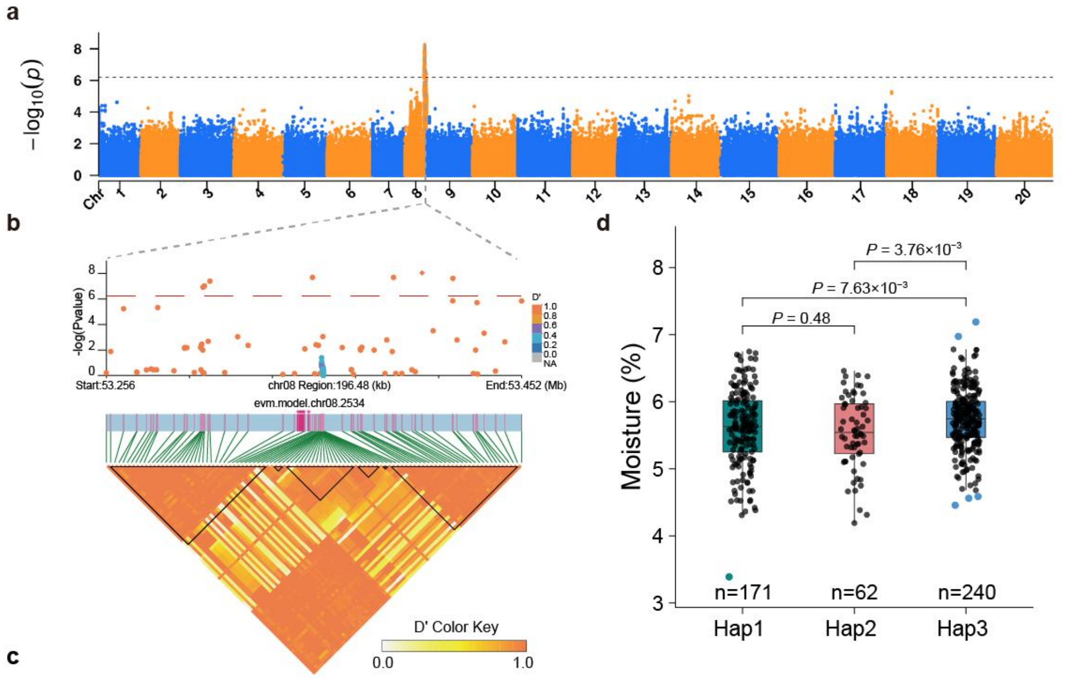

c

Hap	Local 53353397	Local 53349755	Number of samples	Average Moisture(%)	AA
Hap1	0|0 G	0|0 C	171	5.60	
Hap2	0|0 G	1|1 T	62	5.54	5/7 exon; C > T; His > Tyr
Hap3	1|1 A	0|0 C	240	5.74	

Supplementary Notes Fig. 1 | Candidate gene for kernel dehydration rate. a, Manhattan plot for kernel dehydration rate, significant signals at chr08 ($P = 6.26 \times 10^{-7}$; Bonferroni correction). **b,** LD block of a candidate peak within 200-kb interval regions. **c,** Haplotype analysis of Ah2534_EIN2 gene. **d,** Comparison of average kernel dehydration

rate differences between Hap1, Hap2, and Hap3 at population levels. Center line, median; box lower and upper edges, 25% and 75% quartiles, respectively; whiskers, 1.5×IQR; colored dots, outliers. IQR, interquartile range. *P* values were calculated by two-tailed Student's *t*-tests

Candidate gene for arachidic acid content in peanut

Arachidic acid (C20:0), an important component of very long-chain fatty acids (VLCFAs), is associated with the prevalence of atherosclerosis and cardiovascular disease at high levels of VLCFAs[7]. Through GWAS analysis, we identified a significant signal on chromosome 9, with the peak at chr09:115,949,485 (Supplementary Notes Fig.2a, b). Further analysis revealed that a base change from C (Hap1) to G (Hap2) in the second exon of the chr09.2824 gene, annotated as a chaperone protein DNAJ, resulting in an amino acid change (Asp to Glu) (Supplementary Notes Fig.2c). Population analysis showed that 337 samples were of the Hap1 type, with an average arachidic acid content of 1.30%, while 158 samples with the Hap2 type had an average arachidic acid content of 1.17% (Supplementary Notes Fig.2d). It can be speculated that the chr09.2824 gene may have played an important role in regulating the arachidic acid content in peanut seeds.

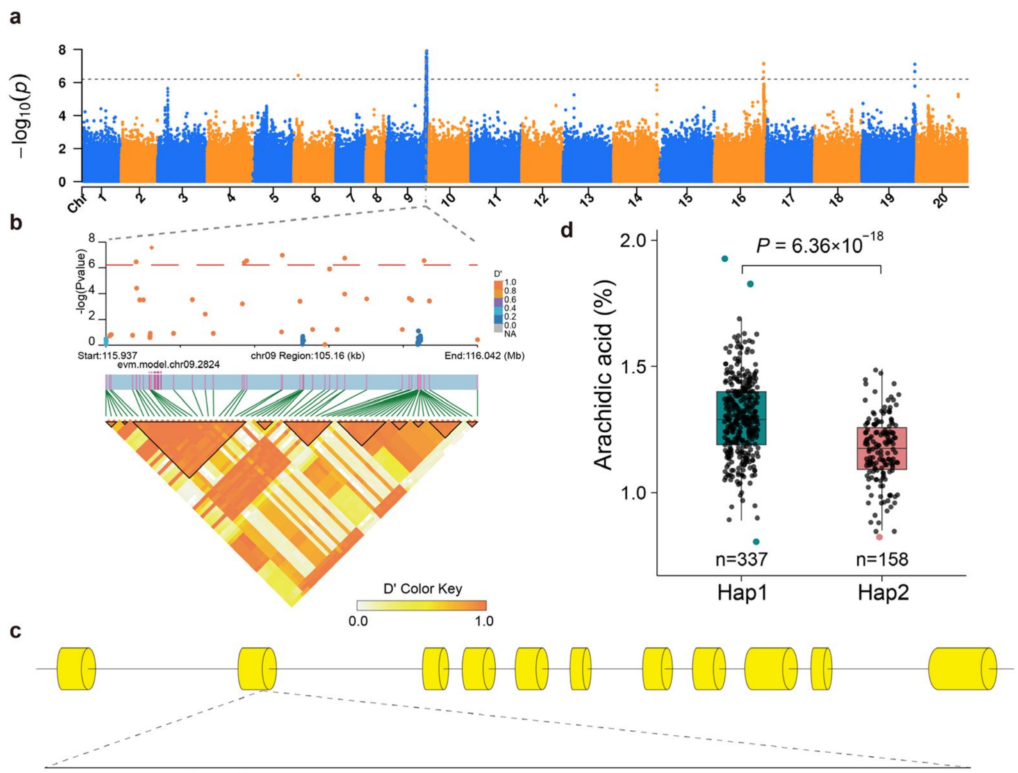

Hap	Local<br>115949485	Number of samples	Average Arachidic acid cotent (%)	AA
Hap1	C	337	1.3	Asp
Hap2	G	158	1.17	Glu

Supplementary Notes Fig. 2 | Candidate gene for arachidic acid content. a, Manhattan plot for arachidic acid content, significant signals at chr09 (P = 6.26 × 10^{-7}; Bonferroni correction). **b,** LD block of a candidate peak within 200-kb interval regions. **c,** Gene structure and haplotype analysis of chr09.2824 gene. **d,** Comparison of average arachidic acid content between Hap1 and Hap2 at population levels. Center line, median; box lower and upper edges, 25% and 75% quartiles, respectively; whiskers, 1.5×IQR; colored dots, outliers. IQR, interquartile range. *P* values were calculated by two-tailed Student's *t*-tests

Significant signals were also identified for other phenotypes (Supplementary Tables 32-34 and Supplementary Fig. 27).

References

5. Su, M. & Hou, S. Ethylene insensitive 2 (EIN2) destiny shaper: The post-translational modification. *J Plant Physiol*. **295**, 154190 (2024).
6. Yu, Y. et al. A Zea genus-specific micropeptide controls kernel dehydration in maize. *Cell*.

188, 44-59 (2025).

7. Huai, D. et al. Enhancing peanut nutritional quality by editing AhKCS genes lacking natural variation. *Plant Biotechnol J*. **22**, 3015–3017 (2024).
