# Supplementary material for: Telomere-to-telomere genome assemblies and population resequencing of diploid and allotetraploid peanut varieties
Source: Nat Genet. 2026 Apr 24;58(5):1151–63. doi: 10.1038/s41588-026-02577-z (PMC13175896; doi:10.1038/s41588-026-02577-z)
Supplement: Supplementary file 2 — Reporting Summary [file 41588_2026_2577_MOESM2_ESM.pdf]

nature portfolio

Corresponding author(s): Rajeev K Varshney; Hang He; Xiaoqin Liu

Last updated by author(s): Feb 1, 2026

Reporting Summary

Nature Portfolio wishes to improve the reproducibility of the work that we publish. This form provides structure for consistency and transparency in reporting. For further information on Nature Portfolio policies, see our Editorial Policies and the Editorial Policy Checklist.

Statistics

For all statistical analyses, confirm that the following items are present in the figure legend, table legend, main text, or Methods section.

n/a	Confirmed	
☐	☒	The exact sample size (*n*) for each experimental group/condition, given as a discrete number and unit of measurement
☐	☒	A statement on whether measurements were taken from distinct samples or whether the same sample was measured repeatedly
☐	☒	The statistical test(s) used AND whether they are one- or two-sided<br>*Only common tests should be described solely by name; describe more complex techniques in the Methods section.*
☒	☐	A description of all covariates tested
☐	☒	A description of any assumptions or corrections, such as tests of normality and adjustment for multiple comparisons
☐	☒	A full description of the statistical parameters including central tendency (e.g. means) or other basic estimates (e.g. regression coefficient) AND variation (e.g. standard deviation) or associated estimates of uncertainty (e.g. confidence intervals)
☐	☒	For null hypothesis testing, the test statistic (e.g. *F*, *t*, *r*) with confidence intervals, effect sizes, degrees of freedom and *P* value noted<br>*Give P values as exact values whenever suitable.*
☒	☐	For Bayesian analysis, information on the choice of priors and Markov chain Monte Carlo settings
☒	☐	For hierarchical and complex designs, identification of the appropriate level for tests and full reporting of outcomes
☒	☐	Estimates of effect sizes (e.g. Cohen's *d*, Pearson's *r*), indicating how they were calculated

Our web collection on statistics for biologists contains articles on many of the points above.

Software and code

Policy information about availability of computer code

Data collection	The resequencing data was collected using the DNBSEQ-T7 platform. HIFI data was collected using the PacBio Sequel II sequencing platform (Pacific Biosciences, CA, USA). ONT data was collected using a PromethION sequencer. Hi-C data was collected using the Illumina NovaSeq 6000 platform. RNA date was collected using Illumina and Sequel II/IIe system (Pacific Biosciences, CA, USA) sequencing platform.
Data analysis	These software include fastp (v.0.22.0); HiC-Pro (v.2.11.1); Hifiasm (v.0.19.5); NUCmer (v.3.1); NextDenovo (v.2.5.0); NextPolish (v.1.4.1); TGS-GapCloser (v.1.2.1); PGA (v.1.0); T2T2-polish (v.1.0); QuarTeT (v.1.0.4); BWA-MEM (v.0.7.17-r1188); minimap2 (v.2.24-r1122); SyRI (v.1.5.3); SVMU (v.4.0.0beta2); Merqury; BUSCO (v.5.4.3); TEsorter (v.1.4.6); MUMmer4(v.4.0.0beta2); Augustus (v.3.5.0); GlimmerHMM (v.3.0.4); Liftoff (v.1.6.3); SAMtools (v.1.16.1); TransDecoder (v.5.5.0); Trinity (v.2.1.1); LTR_FINDER (v1.1); ltrharvest (v.1.6.2); LTR_retriever (v.2.9.0); CD-Hit (v.4.8.1); EVidenceModeler (v.2.1.0); JCVI (v.1.3.4); ParaAT(v.2.0); KaKs_Calculator (v.3.0); TreeBeST (v.1.9.2); PLINK (v.1.9); ADMIXTURE (v.1.3.0); VCFtools (v.0.1.17); PopLDdecay (v.3.40); fastsimcoal2 (v.2.8); XP-CLR (v.1.1.2); R (v.4.3.1); "lme4" package (https://cran.r-project.org/web/packages/lme4/); GAPIT (v.3.5); Stringtie2 (v.2.1.7) ; RepeatMasker(v.4.1.4); HISAT2 (v.2.1.0); HTSeq (v.2.0.3); WGCNA R package (v.1.72); Lastz(v.1.04.22); Genome Analysis Tool Kit (GATK) (v.3.8); Trimmomatic (v.0.36);SVMU (v. 0.4-alpha)

For manuscripts utilizing custom algorithms or software that are central to the research but not yet described in published literature, software must be made available to editors and reviewers. We strongly encourage code deposition in a community repository (e.g. GitHub). See the Nature Portfolio guidelines for submitting code & software for further information.

Data

Policy information about availability of data

All manuscripts must include a data availability statement. This statement should provide the following information, where applicable:

- Accession codes, unique identifiers, or web links for publicly available datasets
- A description of any restrictions on data availability
- For clinical datasets or third party data, please ensure that the statement adheres to our policy

The genome assembly for this project have also been deposited in the National Center for Biotechnology Information (NCBI) database under BioProject number PRJNA1259200 and the National Genomics Data Center, Beijing Institute of Genomics, Chinese Academy of Sciences, under BioProject accession no. PRJCA026588. Additionally, the genome assembly and annotation are also available in the Peanut Genome Database (http://omicsplant.cn/peanut) hosted by the Peking University Institute of Advanced Agricultural Sciences and Figshare (https://figshare.com/articles/dataset/T2T_genome_assembly_in_peanut/28883636). All raw sequencing data for the genome assemblies and annotations, transcriptome and PacBio Iso-Seq reads, and whole-genome sequence data for the population and GWAS analyses have been deposited in the National Center for Biotechnology Information (NCBI) database under BioProject number PRJNA1259747.

Research involving human participants, their data, or biological material

Policy information about studies with human participants or human data. See also policy information about sex, gender (identity/presentation), and sexual orientation and race, ethnicity and racism.

Reporting on sex and gender	N/A
Reporting on race, ethnicity, or other socially relevant groupings	N/A
Population characteristics	N/A
Recruitment	N/A
Ethics oversight	N/A

Note that full information on the approval of the study protocol must also be provided in the manuscript.

Field-specific reporting

Please select the one below that is the best fit for your research. If you are not sure, read the appropriate sections before making your selection.

☒ Life sciences ☐ Behavioural & social sciences ☐ Ecological, evolutionary & environmental sciences

For a reference copy of the document with all sections, see nature.com/documents/nr-reporting-summary-flat.pdf

Life sciences study design

All studies must disclose on these points even when the disclosure is negative.

Sample size	We used 6 peanut accessions for genome assembly and 521 accessions collected worldwide for population genetics analysis.
Data exclusions	No data was exclude.
Replication	Three biologically independent samples were used for transcription and qRT-PCR analysis.
Randomization	A randomized complete block design was used in planting for phenotype data collected in four growing seasons.
Blinding	All accessions were only labeled by numbers when planting and data collection.

Reporting for specific materials, systems and methods

We require information from authors about some types of materials, experimental systems and methods used in many studies. Here, indicate whether each material, system or method listed is relevant to your study. If you are not sure if a list item applies to your research, read the appropriate section before selecting a response.

Materials & experimental systems

n/a	Involved in the study
☒	☐ Antibodies
☒	☐ Eukaryotic cell lines
☒	☐ Palaeontology and archaeology
☒	☐ Animals and other organisms
☒	☐ Clinical data
☒	☐ Dual use research of concern
☐	☒ Plants

Methods

n/a	Involved in the study
☒	☐ ChIP-seq
☒	☐ Flow cytometry
☒	☐ MRI-based neuroimaging

Dual use research of concern

Policy information about dual use research of concern

Hazards

Could the accidental, deliberate or reckless misuse of agents or technologies generated in the work, or the application of information presented in the manuscript, pose a threat to:

No	Yes
☒	☐ Public health
☒	☐ National security
☒	☐ Crops and/or livestock
☒	☐ Ecosystems
☒	☐ Any other significant area

Experiments of concern

Does the work involve any of these experiments of concern:

No	Yes
☒	☐ Demonstrate how to render a vaccine ineffective
☒	☐ Confer resistance to therapeutically useful antibiotics or antiviral agents
☒	☐ Enhance the virulence of a pathogen or render a nonpathogen virulent
☒	☐ Increase transmissibility of a pathogen
☒	☐ Alter the host range of a pathogen
☒	☐ Enable evasion of diagnostic/detection modalities
☒	☐ Enable the weaponization of a biological agent or toxin
☒	☐ Any other potentially harmful combination of experiments and agents

Plants

Seed stocks	*Report on the source of all seed stocks or other plant material used. If applicable, state the seed stock centre and catalogue number. If plant specimens were collected from the field, describe the collection location, date and sampling procedures.*
Novel plant genotypes	*Describe the methods by which all novel plant genotypes were produced. This includes those generated by transgenic approaches, gene editing, chemical/radiation-based mutagenesis and hybridization. For transgenic lines, describe the transformation method, the number of independent lines analyzed and the generation upon which experiments were performed. For gene-edited lines, describe the editor used, the endogenous sequence targeted for editing, the targeting guide RNA sequence (if applicable) and how the editor was applied.*
Authentication	*Describe any authentication procedures for each seed stock used or novel genotype generated. Describe any experiments used to assess the effect of a mutation and, where applicable, how potential secondary effects (e.g. second site T-DNA insertions, mosiacism, off-target gene editing) were examined.*
